# Molecular Weaving of Mixed‐Addenda Polyoxometalates into MOF Nanochannels as Electron‐Buffering Reservoirs for Enhanced Nitrate‐to‐Ammonia Electrocatalysis

**DOI:** 10.1002/advs.202521317

**Published:** 2025-12-25

**Authors:** Gang Li, Xinming Wang, Haijun Pang, Huiyuan Ma

**Affiliations:** ^1^ School of Materials Science and Chemical Engineering Harbin University of Science and Technology Harbin P. R. China

**Keywords:** ammonia synthesis, electrocatalysis, mixed‐addenda, polyoxometalates, POMs‐based metal−organic frameworks

## Abstract

Electrochemical conversion of nitrate pollutants into value‐added ammonia (e‐NO_3_RA) offers a sustainable pathway for nitrogen valorization, yet hinges on the development of efficient electrocatalysts. Herein, we systematically synthesize a series of three V/W mixed‐addenda polyoxometalate ([P_2_W^VI^_17_V^V^_1_O_62_]^7−^, noted as P_2_W_17_V_1_) based metal–organic frameworks [X_9_(DPYB)_9_(OX)_6_(H_2_O)_6_][P_2_W_17_V_1_O_62_] denoted as XMOF‐V_1_ (X = Fe; Co; Zn, DPYB = 1,4‐Di(pyridin‐4‐yl)benzene, OX = oxalic acid). The incorporation of P_2_W_17_V_1_ not only reinforced structural ruggedness and stability, but also promotes multi‐electron transfer, substantially promoting e‐NO_3_RA activity. Among them FeMOF‐V_1_ delivers exceptional electrocatlytic nitrat‐to‐ammonia performance with an achieves over 95% Faradaic efficiency across a wide voltage range of −0.6 to −1.4 V vs RHE. This superiority stems from synergistic interplay: P_2_W_17_V_1_ acts as an electron reservoir enhancing proton‐coupled charge transfer, while the minimal bandgap of FeMOF‐V_1_ facilitates directional electron injection. DFT analyses reveal Fe‐specific mechanisms including strong 3*d*‐orbital hybridization with NO_3_^–^ near Fermi Level (Ef), highly positive Fe sites optimizing adsorption, and transfer weakening N–O bonds, as corroborated by in situ FTIR identification of *NO_3_/*NO/*NH_2_ intermediates. Together with effectively suppressed HER (ΔG_*H_ > ΔG_*NO3_), these attributes collectively enable highly selective NH_3_ synthesis. This study establishes mixed‐addenda POMOFs as a versatile and efficient platform for sustainable nitrate‐to‐ammonia conversion.

## Graphical abstract

Synergistic interaction between P_2_W_17_V_1_ and FeMOF owing to its efficient proton/electron transfer affording high yield and Faradaic efficiency in electrocatalytic nitrate reduction to ammonia.

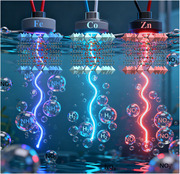

## 1 Introduction

Ammonia (NH_3_), a pivotal inorganic compound supporting global agriculture and industry, is extensively utilized in fertilizer production, pharmaceuticals, refrigeration, and transportation [1, 2]. Its high hydrogen density (17.6 wt.%) and superior transport and storage properties make it an ideal carbon‐free energy carrier [3, 4]. Industrially, NH_3_ production remains dominated by the Haber‐Bosch process, requiring ultrapure N_2_/H_2_ feedstocks under severe conditions (300–500°C, 150–300 atm) [5, 6]. However, this century‐old technology suffers from intrinsic limitations: energy‐intensive operations, complex infrastructure, and significant CO_2_ emissions (accounting for ∼1.8% of global output), collectively hindering sustainable development [7, 8]. The urgent need for economical and eco‐friendly alternatives is therefore imperative amid escalating fossil fuel consumption and environmental pressures.

Electrocatalytic NH_3_ synthesis, powered by renewable electricity with H_2_O as a proton source under ambient conditions, offers a promising sustainable route [9, 10, 11]. While nitrogen reduction reaction (NRR) has attracted broad interest, its practicality is constrained by the inert N≡N bond (941 kJ mol^−1^), low N_2_ solubility, and overwhelming hydrogen evolution competition, leading to unsatisfactory NH_3_ yields (< 337 µg h^−1^ cm^−2^ or 674 µg h^−1^ mg_cat._) [12] and Faradaic efficiencies (< 55%) [13, 14, 15, 16]. Electrocatalytic nitrate (NO_3_^–^) reduction to ammonia (e‐NO_3_RA) presents superior feasibility due to favorable thermodynamics (N═O bond energy: 204 kJ mol^−1^), dual environmental benefits, and high aqueous solubility: (1) valorization of wastewater pollutants (e.g., agricultural/industrial NO_3_^–^) and (2) restoration of disrupted nitrogen cycles [17, 18, 19, 20, 21, 22, 23, 24, 25]. Nevertheless, the eight‐electron transfer process involves intricate pathways with competing byproducts (NO_2_^–^, NO, N_2_O, N_2_), resulting in kinetic bottlenecks and limited NH_3_ selectivity [26, 27, 28, 29]. Designing advanced electrocatalysts to achieve high NH_3_ yield rates and selectivity (> 90% FE) is critically demanded.

Efficiency of electrocatalytic nitrate reduction is fundamentally constrained by mismatched energy barriers between the deoxygenation step (NO_3_^–^ → NO_2_^–^) and subsequent hydrogenation steps (NO_2_^–^ → NH_3_), which promotes toxic intermediate accumulation (e.g., NO_2_^–^) that poisons active sites and compromises product selectivity [30, 31, 32, 33]. To overcome this limitation, strategic integration of electron‐donating groups (EDGs) into catalyst architectures serves as a critical approach for optimizing metal‐site electronic structures. This targeted modulation operates synergistically through enhanced reactant adsorption/activation via elevated electron density at active centers, tuned adsorption energetics from d‐band center downshifting to regulate key intermediate interactions (e.g., *NO, *N), and accelerated charge transfer kinetics [34, 35, 36, 37]. Representative paradigms validate this strategy: Oxygen‐deficient copper nanowires (V‐Cu NAE) employ oxygen vacancies as electron donors to concurrently strengthen NO_3_^–^ adsorption and reduce deoxygenation/hydrogenation barriers [38], FeCo alloys encapsulated in N‐doped carbon nanofibers (FeCo‐NPCNFs) utilize pyridinic‐N‐induced d‐band modulation for optimized *NO_3_/*N adsorption while promoting water‐derived active hydrogen generation [39], BiCu‐MOF/MWCNT hybrids enable formate production at > 100 mA cm^−2^ partial current density through enhanced electron transport [40]. Collectively, these demonstrations confirm that rational EDG engineering precisely tailors active‐site electronic microenvironments, thereby circumventing intrinsic activity‐selectivity trade‐offs in multistep electrocatalytic reductions.

Polyoxometalate‐based metal–organic frameworks (POMOFs) integrate redox‐active POM clusters (e.g., Dawson‐type [P_2_W_18_O_62_]^6–^) as built‐in electron‐donating units within MOF architectures [41, 42, 43]. These hybrid materials intrinsically modulate the electronic states of MOF metal nodes (e.g., Fe, Co, Ni) via direct interfacial electron transfer, bypassing the need for external functionalization [44, 45, 46]. The γ‐CDMOF/GF@S‐based Li–S batteries were experimentally validated, and the introduction of MOF significantly enhanced the stability [47]. The composite material constructed by self‐assembling CoFe‐MOF with PW_9_‐POM exhibits excellent catalytic performance [48]. POMOFs leverage built‐in redox‐active polyanionic clusters as self‐regenerating electron reservoirs to dynamically modulate the electronic structure of MOF metal nodes (Fe/Co/Ni) via interfacial charge transfer. This intrinsic electron donation could downshift d‐band centers, optimizing adsorption energies (*NO_3_^–^, *HNO) to accelerate the rate‐limiting step. Simultaneously, mixed‐addenda POM (e.g., W/V‐POM) harness heterometallic electronic gradients within POM clusters, where higher electronegativity metals donate electrons to adjacent lower electronegativity metals, creating a thermodynamically spontaneous electron flow toward MOF nodes [49]. Furthermore, the high negative charge of POMs creates electrophilic nanodomains that concentrate NO_3_^–^ anions within MOF pores, enhancing mass transfer. This synergistic integration of dynamic electronic modulation, stepwise reaction partitioning, and nanoconfinement effects positions POM@MOFs as paradigm‐shifting catalysts that transcend the limitations of conventional external EDG‐functionalized systems. For example, POM‐porphyrin MOFs achieve > 99% CO Faradaic efficiency in CO_2_RR by shortening electron‐transfer pathways to single‐metal sites [50]. Within monolayer cobalt‐iron hydroxides formed via in situ transformation of CoFe‐MOF@Ni‐POM during electrocatalysis, the Ni‐POM motifs synergistically reduce the oxygen activation energy barrier, establishing a dual stress‐electronic stabilization mechanism [48]. In the polyoxometalate‐based MOF Co_2_(V_4_O_12_)(bpy)_2_, the [V_4_O_12_]^4–^ cluster functions as an electron reservoir that facilitates the activation of peroxymonosulfate (PMS) [51]. These systems establish that directional electron transfer from POM to MOF modulates electronic structures through overcomes kinetic impediments inherent to multi‐proton‐coupled electron transfer processes, thereby enhancing both activity and selectivity in catalytic reductions. Due to their strong electronegativity, POMs attract electrons from the MOFs. This electron withdrawal triggers the transition metals in the MOFs to donate their excited, occupied d‐orbital electrons to the N═O bond, thereby activating the NO_3_^–^ ion. Consequently, integrating POMs with MOFs is expected to provide an ideal platform for the e‐NO_3_RA process. However, the rational design of POMOF electrocatalysts for NO_3_RA remains scarcely explored.

In this work, a series of six catalysts was synthesized to systematically investigate the structure‐activity relationship. On one hand, three Well‐Dawson‐type POMOFs, formulated as [X_9_(DPYB)_9_(OX)_6_·6H_2_O](P_2_W_17_V_1_O_62_) (hereinafter referred to as XMOF‐V_1_, X = Fe, Co, Zn) by the molecular weaving of mixed‐addenda polyoxometalates (P_2_W_17_V_1_O_62_) into MOF nanochannels. This designed structure serves as an efficient electron‐buffering reservoir, establishing a versatile platform for enhanced nitrate‐to‐ammonia electrocatalysis. On the other hand, for mechanistic comparison, three analogous POM‐free frameworks [X(DPYB)(OX)]_2_(DPYB)_0.5_ (hereinafter referred to as XMOF, X = Fe, Co, Zn) were prepared in parallel to serve as critical controls for elucidating the role of the POM unit. These XMOF‐V_1_ architectures leverage the inherent structural ruggedness and exceptional chemical stability of Wells‐Dawson POMs, ensuring robust performance during prolonged e‐NO_3_RA. Critically, the strategic incorporation of vanadium (V^V^) centers within the tungsten (W^VI^)‐dominant POM skeleton significantly enhances catalytic activity by facilitating multi‐electron transfer processes, consistent with the established superiority of V/W mixed‐addenda systems in electrocatalysis. Control analogs XMOF (X = Fe, Co, Zn) exhibited markedly inferior NO_3_RA performance across all metrics compared to their POM‐integrated counterparts. Electrochemical tests under ambient conditions demonstrated FeMOF‐V_1_ delivers good NH_3_ yield (13249 µg h^−1^ mg_cat._^−1^, −1.4 V vs RHE) and highest Faradaic efficiency (98.5%, −0.9 V vs RHE), which are superior not only its mixed‐addenda analogues (CoMOF‐V_1_ and ZnMOF‐V_1_) but also the control MOFs (FeMOF, CoMOF, and ZnMOF) by a twofold improvement. This enhancement is directly attributable to optimized electronic coupling between Fe nodes and W^VI^/V^V^ centers that lowers the potential‐determining step energy barrier.

## 2 Characterization of XMOF‐V1 Catalyst

### 2.1 Characterization of XMOF‐V_1_ Catalyst

**FIGURE 1:**
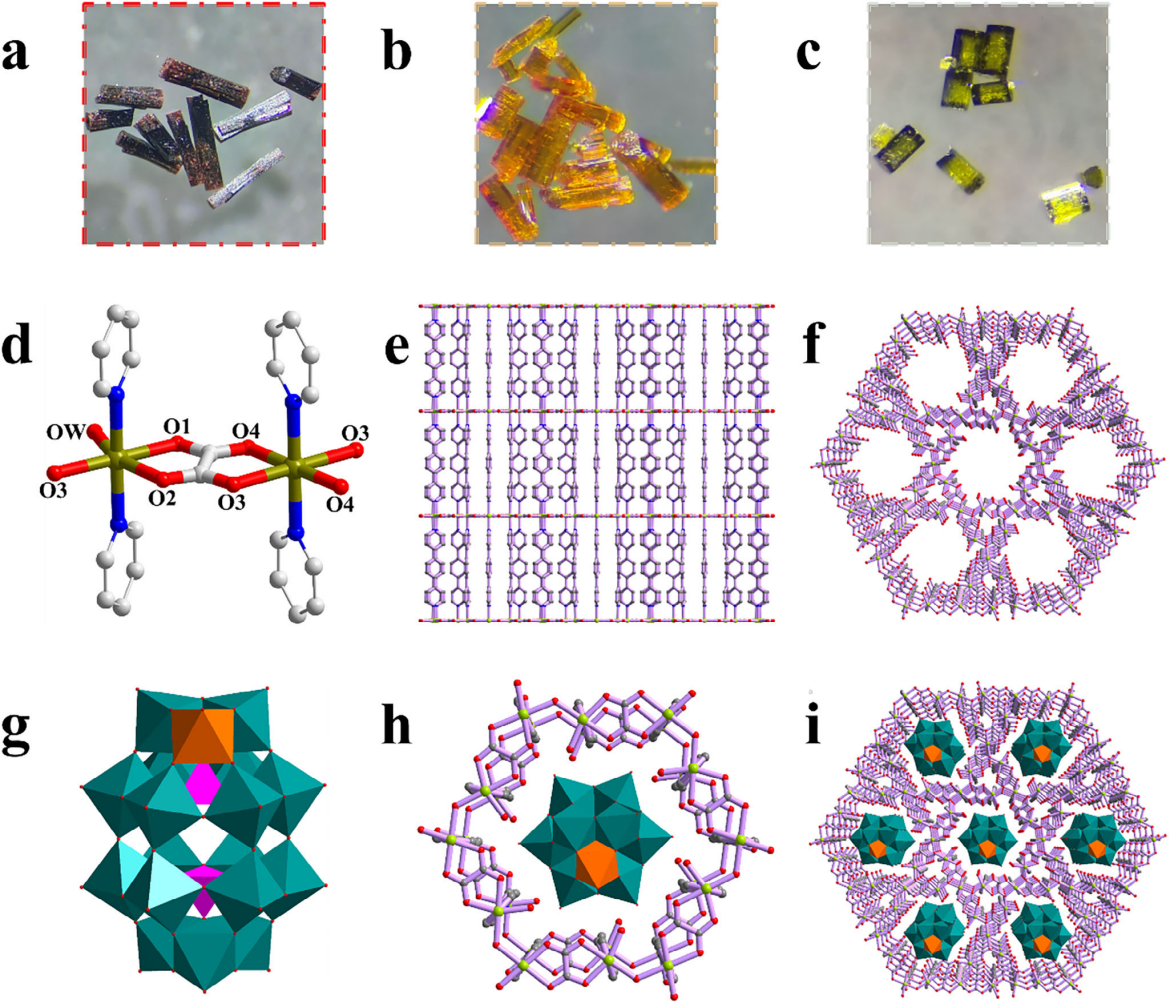
Morphological and structural characterizations of high‐symmetry coordination POMOFs. Macroscopic crystal morphologies of (a) FeMOF‐V_1_, (b) CoMOF‐V_1_, and (c) ZnMOF‐V_1_, respectively. The structural characterizations of high‐symmetry coordination POMOFs. (d) Dinuclear cluster nodes are illustrated. (e), (f) the hexahedral cage structure of the POMOFs. (g) Structure of P_2_W_17_V_1_. (h) View of the asymmetric unit of FeMOF‐V_1_. (i) Overall 3D framework of the POMOFs.

Optical microscopy reveals that all three P_2_W_17_V_1_‐based MOFs (XMOF‐V_1_, X = Fe, Co, Zn) crystallize as well‐defined hexagonal prisms (Figure 1a–c), exhibiting distinct color variations corresponding to their transition metal nodes: dark red (FeMOF‐V_1_), light orange (CoMOF‐V_1_), and dark green (ZnMOF‐V_1_). In contrast, the POM‐free control frameworks display divergent morphologies and hues: FeMOF and ZnMOF form orange‐red and off‐white clavate crystals, respectively (Figures S1 and S2a,c), while CoMOF yields pale‐yellow irregular crystallites (Figure S2b). Scanning electron microscopy (SEM) confirms the hexagonal prismatic habit of XMOF‐V_1_ crystals (Figures S3–S5), with energy‐dispersive X‐ray spectroscopy (EDS) elemental mapping demonstrating that C, N, O, P, W, V, and transition metal X are homogeneously distributed throughout crystal surfaces. Parallel EDS analyses of control MOFs (Figures S6–S14) further validate the exclusive presence of POM‐derived elements (P, W, V) in the XMOF‐V_1_ architectures but absent in the control MOFs, as quantitatively corroborated by elemental ratios in Tables S1–S2.

**FIGURE 2:**
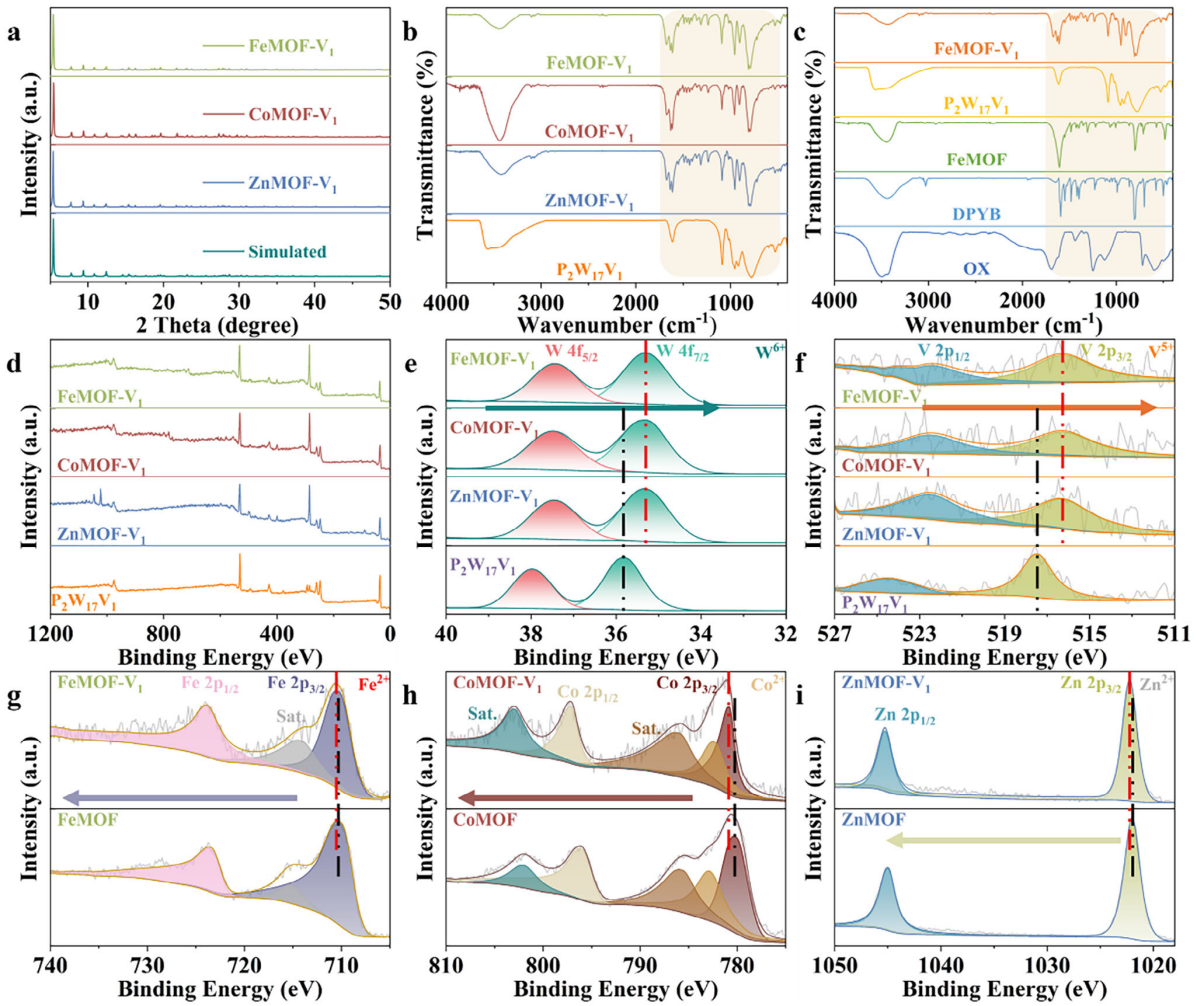
(a) PXRD patterns of XMOF‐V_1_. FTIR spectra of (b) XMOF‐V_1_ and (c) FeMOF‐V_1_, FeMOF, P_2_W_17_V_1_, DPYB, OX. XPS spectra of XMOF‐V_1_: (d) survey, (e) W 4f, (f) V 2p, (g) Fe 2p, (h) Co 2p, (i) Zn 2p.

Single‐crystal X‐ray diffraction confirms that XMOF‐V_1_ (X = Fe, Co, Zn) crystallize in isostructural frameworks [52], differing only in their transition metal nodes (Fe/Co/Zn). Structurally, XMOF‐V_1_ features two crystallographically independent metal centers (Figure 1d). The X1 center adopts a hexa‐coordinated geometry, with two nitrogen atoms from two DPYB ligands and four other atoms completing its coordination sphere. The X2 center likewise adopts a hexa‐coordinated geometry, with its coordination satisfied by two nitrogen atoms from two DPYB ligands, three oxygen atoms from two OX^2^^−^ ligands, and one oxygen atom from a water molecule (OW). In XMOF‐V_1_, metal dimers are initially bridged by oxalate anions. These dimeric units are subsequently interconnected via an oxygen atom, forming a twelve‐membered hexagonal ring composed of Fe, Co, or Zn atoms. These hexagonal twelve‐membered rings are interconnected by DPYB ligands, which are oriented nearly perpendicular to the layer plane. This connectivity generates a framework featuring both hexagonal and triangular channels that extend perpendicularly through the structure (Figure 1e,f). The hexagonal channels are composed of the twelve‐membered rings that surround the P_2_W_17_V_1_ (Figure 2g) polyoxoanions that act as templates (Figure 2h). And a near‐perfect size match exists between the internal diameter of the hexagonal channels (12.293 Å, Figure S15) and the P_2_W_17_V_1_ polyoxoanion (11.0 Å, Figure S16), leading to an effective encapsulation (Figure 2i). Consequently, this optimal fit facilitates a compact structure with an extremely short POM‐MOF center distance of only 4.99 Å (Figure S17), which is in favor of the directional transport of electrons from P_2_W_17_V_1_ to XMOF [53].

Powder X‐ray Diffraction analysis (PXRD) confirms the isostructural nature of XMOF‐V_1_ series, with experimental patterns exhibiting excellent agreement with simulations derived from their respective single‐crystal structures (Figure 2a; Figure S18) [52]. From the Fourier transform infrared spectroscopy (FTIR) spectra of XMOF‐V_1_ (Figure 2b), XMOF (Figure S19), and P_2_W_17_V_1_ (Figure S20). The FTIR spectrum of P_2_W_17_V_1_ shows four characteristic bands at 1088, 953, 899, 809 cm^−1^ that are attributed to *v*_M‐Od_, *v*_P‐Oa_, *v*_M‐Ob‐M_, *v*_M‐Oc‐M_ (M = V and W) vibrations, respectively [53]. The three XMOF‐V_1_ also show similar peak positions (Figure 2c). The FTIR spectrum of XMOF‐V_1_ and XMOF in the 1669–1238 cm^−1^ region, attributed to DPYB and OX^2−^. PXRD and FTIR data conclusively verify phase purity and structural retention across synthesized catalysts.

The surface composition of three XMOF‐V_1_, three XMOF, and P_2_W_17_V_1_ is analyzed by X‐ray photoelectron spectra (XPS) (Figure 2d; Figure S21). The XPS results indicate that high‐resolution W 4f spectra of three XMOF‐V_1_ and P_2_W_17_V_1_ catalysts (Figure 2e) show two peaks, which are attributed to the W 4f_5/2_ and W 4f_7/2_ binding energies for W^6+^ (FeMOF‐V_1_: 35.50 and 37.64 eV, CoMOF‐V_1_: 35.30 and 37.50 eV, ZnMOF‐V_1_: 35.40 and 37.54 eV, P_2_W_17_V_1_: 35.80 and 37.98 eV) [54]. The W 4f_7/2_ peaks of FeMOF‐V_1_ (CoMOF‐V_1_ and ZnMOF‐V_1_) are shifted negatively ∼0.3 eV (0.5 and 0.4 eV) with P_2_W_17_V_1_. High‐resolution V 2p XPS spectra (Figure 2f) reveal characteristic doublets for V^5+^ species across all catalysts, with binding energies at 516.43 and 523.14 eV (FeMOF‐V_1_), 516.25 and 522.50 eV (CoMOF‐V_1_), 516.33 and 522.58 eV (ZnMOF‐V_1_), 517.44 and 524.47 eV (P_2_W_17_V_1_) corresponding to V 2p_3/2_ and V 2p_1/2_ transitions [54]. Crucially, the V 2p_3/2_ peaks in the hybrid frameworks exhibit pronounced negative shifts of ∼1.01 eV (FeMOF‐V_1_), 1.19 eV (CoMOF‐V_1_), and 1.11 eV (ZnMOF‐V_1_) relative to pristine P_2_W_17_V_1_, demonstrating systematic electron density enrichment at vanadium centers upon integration into XMOF matrices. In Fe 2p spectra (Figure 2g), the binding energies at 710.51 and 723.60 eV (FeMOF‐V_1_), 710.38 and 723.58 eV (FeMOF) are assigned to Fe^2+^ [55, 56, 57, 58]. A satellite peak (noted as “sat.”) with binding energies of 714.42 and 715.38 eV can be observed in FeMOF‐V_1_ and FeMOF, assigned to Fe^2+^ [55, 56, 57, 58]. The Fe 2p3/2 peak of FeMOF‐V1 is shifted to higher energies ∼0.13 eV in comparison with FeMOF. In Co 2p spectra (Figure 2h), binding energies at 780.88, 782.58, 797.18 eV (CoMOF‐V_1_) and 780.38, 782.98, 796.18 eV (CoMOF) are assigned to Co^2+^ [55, 56, 57, 58]. A satellite peak with binding energies of 786.38, 803.08 eV and 785.98, 802.18 eV can be observed in CoMOF‐V_1_ and CoMOF, assigned to Co^2+^ [55, 56, 57, 58]. The Co 2p3/2 peak of CoMOF‐V1 is shifted to higher energies ∼0.5 eV in comparison with the CoMOF. In Zn 2p spectra (Figure 2i), binding energies at 1022.18, 1045.28 eV (ZnMOF‐V_1_) and 1021.98, 1045.08 eV (ZnMOF) are assigned to Zn^2+^ [55, 56, 57, 58]. The Zn 2p3/2 peak of ZnMOF‐V_1_ is shifted to higher energies ∼0.2 eV in comparison with ZnMOF. Observed negative binding energy shifts for W^6+^ and V^5+^ in P_2_W_17_V_1_ units, coupled with positive shifts in the Fe/Co/Zn nodes of the MOF framework, collectively demonstrate electron transfer between the POM and MOF components, providing direct evidence for the formation of a built‐in electric field [50].

## 3 Electrocatalytic Nitrate‐to‐Ammonia Performance

**FIGURE 3:**
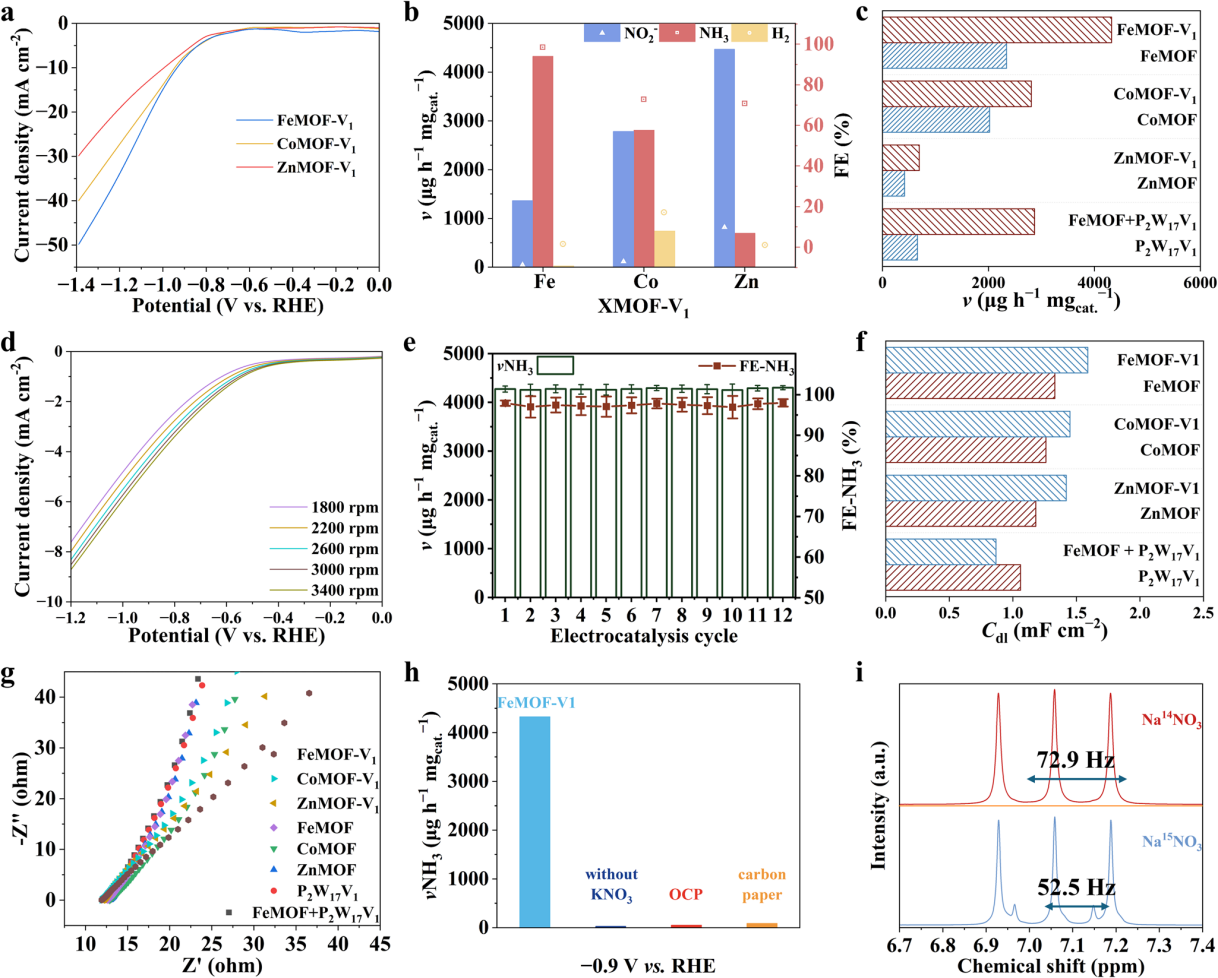
E‐NO_3_RA performance. (a) LSV of XMOF‐V_1_. NH_3_ yield rates and FEs of (b) XMOF‐V_1_, (c) each catalyst at −0.9 V vs RHE. (d) Rotating rate‐dependent LSV curves with FeMOF‐V_1_ deposited on the disk of the RDE working electrode. (e) NH_3_ yield rates and FE‐NH_3_ for 12 cycles of FeMOF‐V_1_. (f) ECSA of all compounds. (g) EIS of all samples. (h) NH_3_ yield of each catalysts. (i) ^1^H NMR spectra of K^14^NO_3_ or K^15^NO_3_ (10atom%) as the feeding nitrate source using FeMOF‐V_1_.

Electrocatalytic nitrate reduction to ammonia (e‐NO_3_RA) performance was evaluated in a H‐type cell (0.05 mol L^−1^ Na_2_SO_4_ with 0.1 mol L^−1^ KNO_3_). The catalysts were deposited onto carbon paper (CP) with a mass loading of 0.33 mg cm^−2^. Comparative linear sweep voltammetry (LSV) measurements of XMOF‐V_1_, pristine XMOF, and P_2_W_17_V_1_ (Figures S22a–S29a) in nitrate‐containing (0.05 mol L^−1^ Na_2_SO_4_ with 0.1 mol L^−1^ KNO_3_) versus nitrate‐free electrolytes (0.05 mol L^−1^ Na_2_SO_4_) reveal density enhancement under nitrate conditions, demonstrating catalytic activity toward nitrate reduction across all materials. Electrochemical benchmarking via LSV (Figure 3a) demonstrates a metal‐dependent activity hierarchy (Fe > Co > Zn) in XMOF‐V_1_ catalysts, with the FeMOF‐V_1_ exhibiting maximal current density, indicating enhanced catalytic proficiency toward e‐NO_3_RA [59].

The e‐NO_3_RA tests were performed at a series of applied potentials (−0.4 to −1.4 V vs reversible hydrogen electrode, RHE) in corresponding electrolytes. With increase of applied potential, FE values of XMOF‐V_1_ showed volcano‐shaped activity curves in 0.05 mol L^−1^ Na_2_SO_4_ with 0.1 mol L^−1^ KNO_3_, reaching their maximum values at −0.9 V vs RHE (Figure 3b). Specifically, FeMOF‐V_1_ electrode demonstrated superior performance, achieving a maximum FE of 98.50% (NH_3_ yield rate: 4323 µg h^−1^ mg_cat._^−1^) at −0.9 V vs RHE, significantly outperforming both CoMOF‐V_1_ (72.88%, 2809 µg h^−1^ mg_cat._^−1^) and ZnMOF‐V_1_ (51.83%, 694 µg h^−1^ mg_cat._^−1^). The amount of nitrite ion (NO_2_^−^) byproduct detected are followed by FeMOF‐V_1_ (1.04%, 493 µg h^−1^ mg_cat._^−1^) < CoMOF‐V_1_ (2.41%, 1004 µg h^−1^ mg_cat._^−1^) < ZnMOF‐V_1_ (16.48%, 1611 µg h^−1^ mg_cat._^−1^). Notably, FeMOF‐V_1_ generated only a trace amount of H_2_ (0.46%; 9.48 µg h^−1^ mg_cat._^−1^), significantly outperforming both CoMOF‐V_1_ (14.75%; 267.52 µg h^−1^ mg_cat._^−1^) and ZnMOF‐V_1_ (2.41%; 17.83 µg h^−1^ mg_cat._^−1^) in suppressing competing hydrogen evolution, thus affirming its superior selectivity toward NH_3_. The corresponding nitrogenous product distributions are provided in Figures S22–S24. As shown in Table S3, FeMOF‐V_1_ outperforms most of the referenced catalysts in terms of balanced FE, NH_3_ yield rate, and low applied potential, which clearly highlights its relative advantage in NO_3_RA. All cited works (including the two recommended ones) have been properly added to the reference list.

To elucidate the critical role of P_2_W_17_V_1_ polyoxometalate in the electrocatalytic system, we evaluated the control samples‐namely, the pristine P_2_W_17_V_1_ and the three XMOF frameworks without POM integration‐for e‐NO_3_RA. In Figure 3c and Figures S25–S27, the activity of the XMOF controls was substantially lower than that of their XMOF‐V_1_ counterparts, unequivocally highlighting the indispensable role of P_2_W_17_V_1_ in achieving high‐performance e‐NO_3_RA. The pristine P_2_W_17_V_1_ alone showed negligible catalytic activity (659 µg h^−1^ mg_cat._^−1^, 58.34%; Figure S28), confirming its inability to drive the reaction independently. Furthermore, as expected, the FeMOF shows a superior NH_3_ yield rate (2341 µg h^−1^ mg_cat._^−1^) and FE (91.05%) compared to CoMOF (2020 µg h^−1^ mg_cat._^−1^ and 49.18%) and ZnMOF (416 µg h^−1^ mg_cat._^−1^ and 27.53%), FeMOF+P_2_W_17_V_1_ (2871 µg h^−1^ mg_cat._^−1^ and 96.48%, Figure S29) through physical blending exhibits lower performance than FeMOF‐V_1_, establishing the Fe center as an active metallic node. Moreover, the conversion of NO_3_^−^ is also an important metric for evaluating the catalytic reactivity in NO_3_RA; as shown in Figure S30 and Table S4, FeMOF‐V_1_ exhibits excellent performance in both selectivity and conversion (FeMOF‐V_1_:98.13% and 15.59%, CoMOF‐V_1_:90.09% and 6.97%, ZnMOF‐V_1_: 51.78% and 2.31%, respectively). Collectively, these comparative results lead to two critical conclusions regarding the FeMOF‐V_1_ catalyst: 1) Fe centers serve as primary catalytic active sites for e‐NO_3_RA, rather than the P_2_W_17_V_1_ units. 2) The incorporated P_2_W_17_V_1_ plays a pivotal role in significantly accelerating and facilitating electron transfer, thereby dramatically enhancing overall efficiency. To clarify the electron transfer pathway and rate‐limiting step of NO_3_RA on FeMOF‐V_1_, linear sweep voltammetry (LSV) curves were recorded at various rotating speeds (Figure 3d). The corresponding Koutecky–Levich (K‐L) plots at −0.8 to −1.2 V vs RHE exhibit excellent linearity and near‐perfect coincidence (Figure S31), indicating a diffusion‐controlled reaction process, consistent with nitrate adsorption on the catalyst surface being the rate‐limiting step (as supported by the referenced Small study). Further analysis via the K‐L equation reveals a 6‐electron transfer process for nitrate reduction over FeMOF‐V_1_. This aligns with the direct reduction of NO_2_^–^ to NH_3_ (N valence state: +3 → −3), confirming the high selectivity for NH_3_ formation, correctly reflecting the NO_3_RA pathway [60, 61].

Catalyst stability is another critical issue for general electrocatalysts under working conditions. After 48‐h immersion in the electrolyte, the three XMOF‐V_1_ catalysts retain their original PXRD and FTIR patterns, indicating their high stability chemical and structural stability (Figures S32 and S33). Consecutive recycling tests were performed at −0.9 V vs RHE for three XMOF‐V_1_ catalysts. Three XMOF‐V_1_ catalysts exhibited stable NH_3_ yield rates and FEs over 12 consecutive cycles (Figure 3e; Figures S34−S36). Furthermore, the FTIR spectra of the catalysts before and after the 12 consecutive cycles test remained virtually unchanged (Figure S37), providing additional evidence for their excellent structural stability. XPS was performed to characterize the post‐catalysis material (Figure S39), aiming to verify the stability of the chemical states of Fe, W, and V. The XPS spectra reveal that the binding energies of Fe, W, and V in the used catalyst remain consistent with those of the fresh FeMOF‐V_1_, without significant shifts. This confirms that the valence states of these key elements (which are closely related to the catalyst's active sites) remain unchanged after extended electrocatalytic cycling, indicating no oxidation‐reduction‐induced structural degradation of the active components or the FeMOF‐V_1_ framework. Furthermore, inductively coupled plasma mass spectrometry (ICP‐MS) was employed to analyze the electrolyte after extended NO_3_RA cycling, aiming to evaluate the leaching behavior of key metal elements (W and V, core components of POM units) and verify the structural integrity of the POMOF framework. As summarized in Table S5, only trace amounts of W and V were detected in the electrolyte for all XMOF‐V_1_ samples: the concentrations of W and V in FeMOF‐V_1_ are as low as 5.68 × 10^−5^ µg/L and 0.71 × 10^−5^ µg/L, respectively, while those in CoMOF‐V_1_ (2.18 × 10^−5^ µg/L for W, 1.16 × 10^−5^ µg/L for V) and ZnMOF‐V_1_ (6.82 × 10^−5^ µg/L for W, 2.44 × 10^−5^ µg/L for V) are similarly negligible. Such ultralow leaching concentrations confirm that POM units are tightly embedded within the MOF framework, with no significant dissociation or structural collapse during electrocatalysis. The negligible loss of active metal components not only reflects the robust interaction between POM and MOF but also directly demonstrates the excellent catalytic stability of XMOF‐V_1_ catalysts, ensuring consistent active site density and catalytic performance over extended reaction cycles.

Electrochemical surface areas (ECSA) were evaluated by determining the double‐layer capacitance (*C*_dl_) from the cyclic voltammetry (CV) measurements [62]. The CV curves are measured in a potential window of 450–550 mV range at scan rates varying from 10 to 100 mV s^−1^ in 0.05 mol L^−1^ Na_2_SO_4_ containing 0.1 mol L^−1^ KNO_3_ (Figure 3f; Figure S41). The derived *C*_dl_ values provide insight into the enhanced e‐NO_3_RA activity: 1) FeMOF‐V1 exhibits the largest *C*_dl_ value (1.59 mF cm^−2^) among the XMOF‐V_1_ series (CoMOF‐V_1_: 1.45 mF cm^−2^, ZnMOF‐V_1_: 1.42 mF cm^−2^); 2) all POM‐incorporated XMOF‐V_1_ exhibit significantly larger *C*_dl_ values than their parent XMOFs (FeMOF: 1.33 mF cm^−2^, CoMOF: 1.26 mF cm^−2^, and ZnMOF: 1.18 mF cm^−2^), and the pure P_2_W_17_V_1_ (1.06 mF cm^−2^). To further elucidate the possible effects, electrochemical impedance spectroscopy (EIS) characterizations were conducted. FeMOF‐V_1_ exhibits the smallest charge‐transfer resistance in the Nyquist plots (Figure 3g; Figure S42 and Table S6), demonstrating the most efficient electron transfer. This trend is consistent across the series, with the XMOF‐V_1_ catalysts demonstrating superior charge‐transfer capability compared to the XMOF controls. FeMOF‐V_1_ presents a smaller charge transfer resistance than the other materials (CoMOF‐V_1_ and ZnMOF‐V_1_), meanwhile the XMOF‐V_1_ catalysts demonstrate superior charge‐transfer capability compared to the XMOF controls. Collectively, these electrochemical analyses confirm that the molecular weaving of P_2_W_17_V_1_ into the MOF framework effectively expands the ECSA and accelerates electron transfer. This synergistic effect is particularly crucial for facilitating the complex multi‐electron/proton transfer processes inherent in e‐NO_3_RA.

Control experiments were carried out to pinpoint the origin of the NH_3_: 1) FeMOF‐V_1_ electrolyte under 0.05 mol L^−1^ Na_2_SO_4_ without 0.1 mol L^−1^ KNO_3_ electrolyte, 2) 0.05 mol L^−1^ Na_2_SO_4_ with 0.1 mol L^−1^ KNO_3_ electrolyte at the open circuit potential (OCP), and 3) CP as the working electrode with 0.05 mol L^−1^ Na_2_SO_4_ containing 0.1 mol L^−1^ KNO_3_ electrolyte. As expected, there was almost no detection of NH_3_ production (Figure 3h; Figure S43). The above experiments revealed that NH_3_ is obtained by the electro‐reduction reaction of NO_3_^−^ instead of other ways (such as NH_3_ contamination from the glassware or atmosphere). In order to further verify the source of NH_3_, we have performed N‐isotope labeling experiments. When K^15^NO_3_ was used as the electrolyte, the^1^H nuclear magnetic resonance (NMR) spectrum after potentiostatic polarization and concentration revealed a characteristic doublet (*J* = 72.9 Hz, Figure 3i; Figure S44) of equal intensity. This signal is attributable to ^15^NH_4_^+^, in contrast to the triplet (*J* = 52.5 Hz) characteristic of ^14^NH_4_^+^ [63, 64, 65]. These results confirm that the produced NH_3_ originates exclusively from the electrochemical reduction of NO_3_^−^ on the FeMOF‐V_1_ electrocatalyst.

## 4 Understanding the Possible Mechanism in Catalytic Activity

**FIGURE 4:**
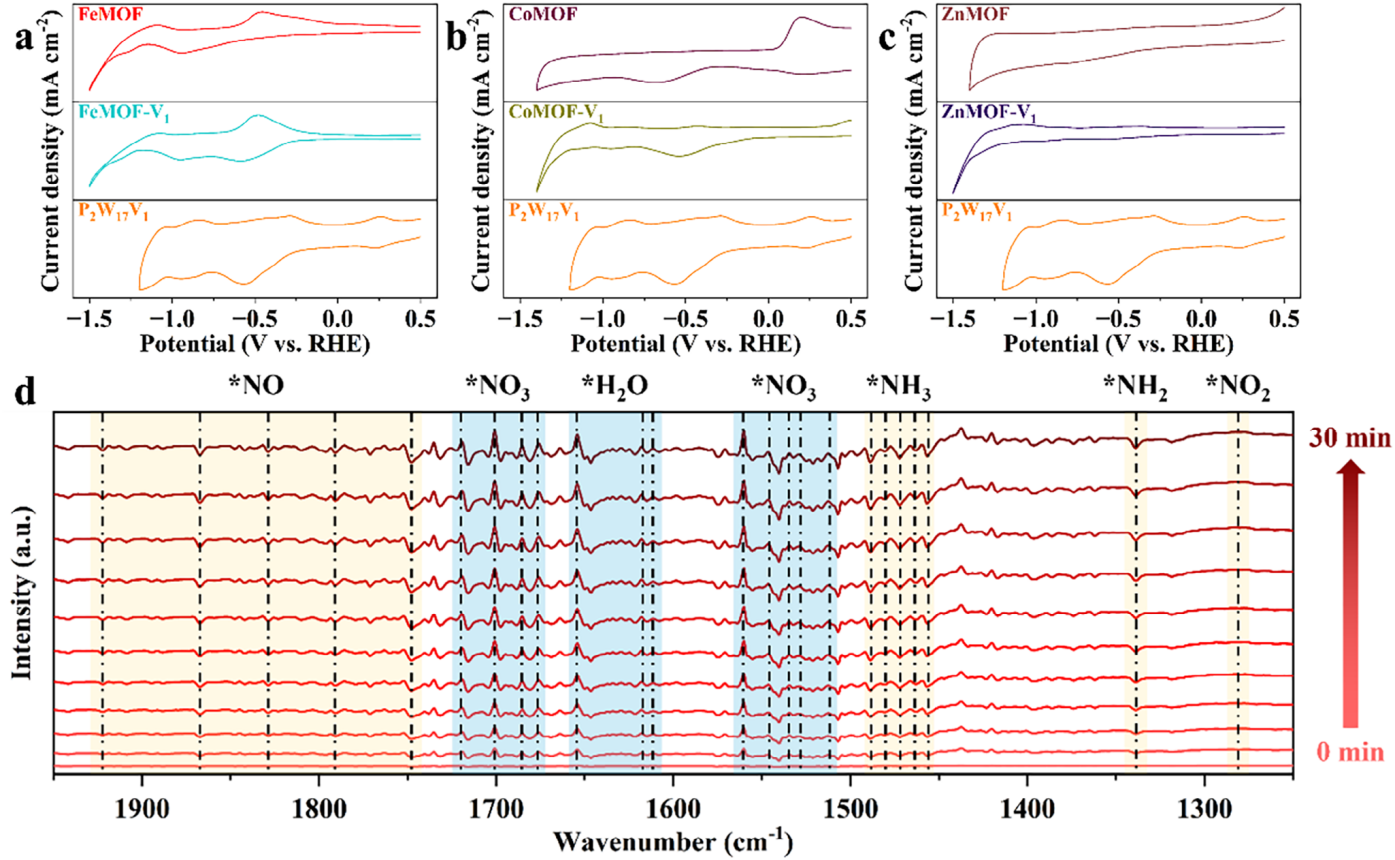
CVs for (a) FeMOF, FeMOF‐V_1_, P_2_W_17_V_1_, (b) CoMOF, CoMOF‐V_1_, P_2_W_17_V_1_, (c) ZnMOF, ZnMOF‐V_1_, P_2_W_17_V_1_. (d) Time‐dependent in situexternal reflection FTIR spectra. Spectra on FeMOF‐V_1_ electrodes from 0 to 30 min.

To elucidate the roles of P_2_W_17_V_1_ and metal sites in e‐NO_3_RA efficiency, cyclic voltammetry (CV) was conducted. Comparative analysis of pure P_2_W_17_V_1_ (tetrabutylammonium salt), XMOF, and XMOF‐V_1_ (X = Fe, Co, Zn) revealed distinct redox signatures. XMOF samples exhibit two reduction peaks (FeMOF: −0.942 and −1.273 V, CoMOF: 0.181 and −0.693 V, ZnMOF: −0.563 and −0.813 V vs RHE), assigned to metal‐centered reduction (Figure 4a–c). In contrast, the pristine P_2_W_17_V_1_ displays four well‐defined reduction peaks at 0.227, −0.421, −0.569, and −0.923 V vs RHE. Notably, reduction events of P_2_W_17_V_1_ occur at more positive potentials than those of the XMOF frameworks, underscoring its superior electron affinity and suggesting its role as a preferential electron acceptor within the integrated catalytic system.

To gain insights into the intrinsic electrocatalysis mechanism during the e‐NO_3_RA process, we performed in situ external reflection FTIR to monitor the evolution of adsorbed intermediates on the electrode surfaces of FeMOF‐V_1_ during the electroreduction of NO_3_^–^ to NH_3_. As depicted in Figure 4d, the upward adsorption bands in the ranges of 1500−1565 cm^−1^ and 1662−1726 cm^−1^ correspond to adsorbed *NO_3_ species [66, 67], indicating continuous NO_3_^−^ consumption over time. Concurrently, the upward bands in 1610−1660 cm^−1^ are attributed to the HOH bending (δHOH) mode of H_2_O [68], reflecting water electrolysis as the proton source. Meanwhile, downward bands observed at 1735−1942 cm^−1^ are characteristic of *NO adsorbates on Fe sites [66, 69], while those at 1292 and 1330 cm^−1^ are assigned to *NO_2_ and *NH_2_ intermediates, respectively [66, 70]. Furthermore, the appearance of downward adsorption bands in 1420−1490 cm^−1^ confirms the generation of NH_3_ [71].

**FIGURE 5:**
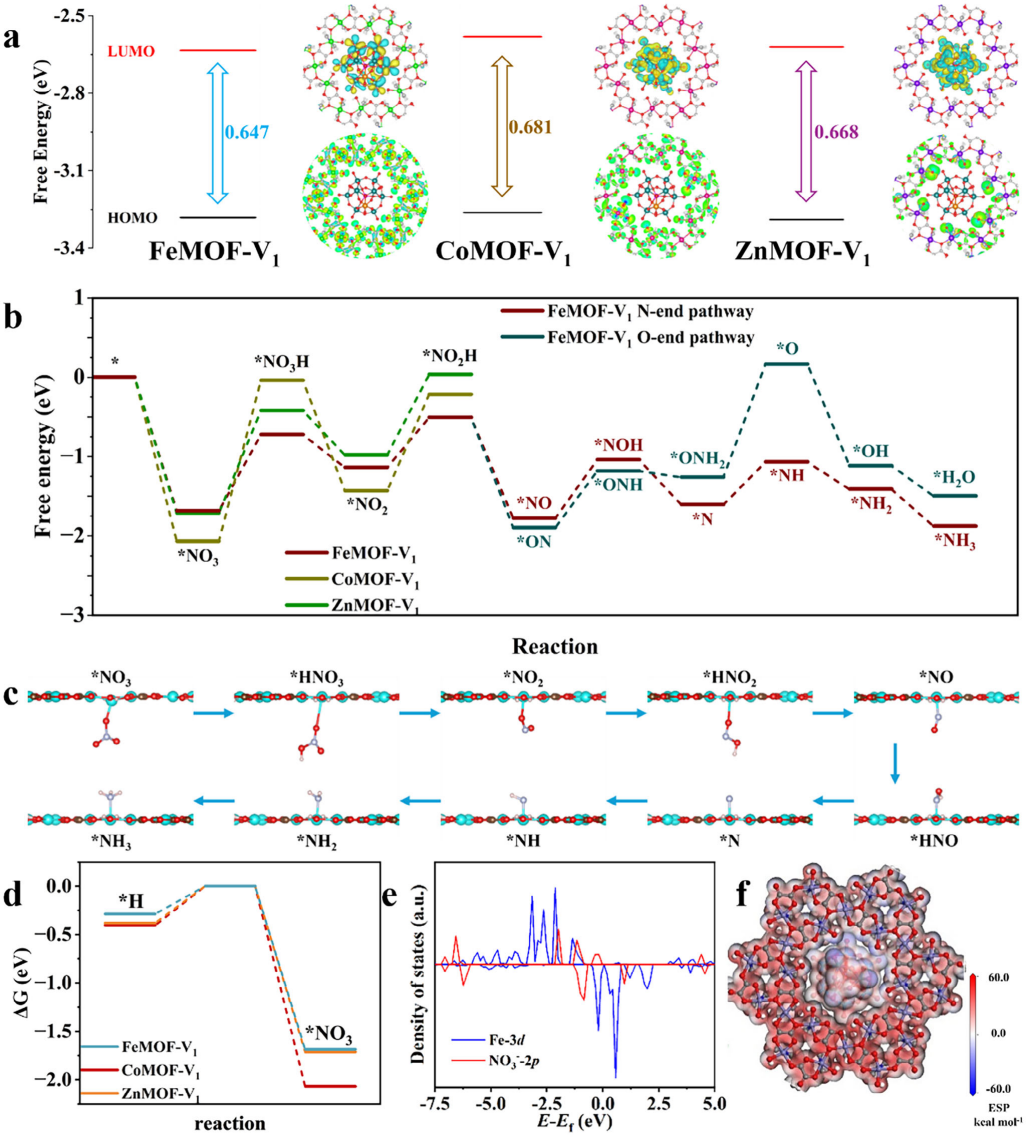
Theoretical calculations. (a) Computed frontier orbitals for FeMOF‐V_1_, CoMOF‐V_1_, ZnMOF‐V_1_. (b) Free energy diagram of electrochemical conversion from NO_3_^–^ to NH_3_ in XMOF‐V_1_ systems and the corresponding intermediate structures are shown in (c) and (d) HER for XMOF‐V_1_ systems. (e) PDOS analysis of Fe‐3*d* and NO_3_^−^‐2*p* adsorbed on the FeMOF‐V_1_ model. (f) Electrostatic potential (ESP) surface of the FeMOF‐V_1_ model. Color scheme for balls: Fe (blue), C (brown), N (wathet), O (red), H (pink) respectively.

To probe the electron storage and transfer behavior of XMOF‐V_1_ materials during electrocatalysis, we performed systematic Kohn‐Sham density functional theory (DFT) calculations employing the standard B3LYP functional. DFT calculations reveal FeMOF‐V_1_ exhibits the narrowest HOMO‐LUMO gap (0.647 eV) among the series, followed by ZnMOF‐V_1_ (0.668 eV) and CoMOF‐V_1_ (0.681 eV), with corresponding HOMO/LUMO energies of –2.634/–3.281 eV (FeMOF‐V_1_), –2.621/–3.289 eV (ZnMOF‐V_1_), and –2.581/–3.262 eV (CoMOF‐V_1_). Solid‐state UV–vis results confirm the same trend: FeMOF‐V_1_ has the smallest optical bandgap (1.90 eV), compared to 1.94 eV (ZnMOF‐V_1_) and 1.96 eV (CoMOF‐V_1_) (Figure S45). Notably, the orbital distributions of XMOF‐V_1_ materials show high similarity in overall configuration, while the HOMO and LUMO exhibit distinct spatial distributions (central part of Figure 5b), a feature that modulates electron transfer dynamics. Combined with higher HOMO energy of FeMOF‐V_1_ and narrower gaps, this electronic structure configuration enhances its reducing capability, providing a rational explanation for its superior electrocatalytic performance in nitrate reduction [72].

The Gibbs free energy diagrams for possible e‐NO_3_RA pathways were constructed based on DFT computations (Figure 5b–f). Evaluation of the Gibbs free energy profiles for various e‐NO_3_RA pathways on the FeMOF‐V_1_ model (Figure 5b) reveals that the process initiates with NO_3_^−^ adsorption at the metal site—a crucial step governing subsequent reaction sequences. The adsorbed NO_3_^−^ undergoes protonation to form *HNO_3_ intermediate, with the potential‐determining step (PDS) exhibiting progressively higher energy barriers from FeMOF‐V_1_ (ΔG = 0.962 eV) to CoMOF‐V_1_ (ΔG = 2.026 eV) and ZnMOF‐V_1_ (ΔG = 1.291 eV), consistent with the experimental activity trend FeMOF‐V_1_ > CoMOF‐V_1_ > ZnMOF‐V_1_. The superior performance of FeMOF‐V_1_ originates from its reduced energy requirement for *NO_3_H hydrogenation. Subsequent *NO_2_ to *HNO_2_ conversion further confirms this trend with energy increases of ∼0.63 eV (FeMOF‐V_1_) < 1.214 eV (CoMOF‐V_1_) < 1.016 eV (ZnMOF‐V_1_), while CoMOF‐V_1_ and ZnMOF‐V_1_ demonstrate relatively better performance in NO_2_^−^ generation due to their higher energy demands for NO_2_H hydrogenation. We monitored the time evolution of NO_2_^−^ under a constant applied potential of −0.9 V vs RHE. We monitored the time evolution of NO_2_^−^ under a constant applied potential of −0.9 V vs RHE (Figure S46). As the reaction proceeded, the yield of NH_3_ increased steadily, while the concentration of NO_2_^−^ remained consistently very low. This result indicates the suppression of NO_2_^−^ formation.

Further investigation of possible e‐NO_3_RA pathways examined adsorbed NO intermediates with N‐end and O‐end configurations, with optimized geometries shown in Figure 5b–c and Figures S47–S49. The O‐end pathway presents a significant energetic challenge during *ONH_2_ hydrogenation to *O intermediate (ΔG ≈ 1.424 eV), thereby rendering the N‐end pathway preferential due to its lower energy barrier. The *N intermediate subsequently undergoes exothermic reduction to NH_3_ through three proton‐electron transfer steps at metal sites, followed by facile NH_3_ desorption to complete the catalytic cycle. Importantly, computed hydrogen binding strength (ΔG_*H_) remains weaker than NO_3_^−^ adsorption across all three XMOF‐V_1_ structures (Figure 5d), confirming the thermodynamic preference for e‐NO_3_RA over the HER. The initial NO_3_^−^ adsorption thus represents a critical determinant for the overall catalytic efficiency.

Projected density of states (PDOS) analysis of *NO_3_ adsorption on the FeMOF‐V_1_ model revealed strong hybridization between the Fe *3d* orbitals and NO_3_^−^ species, with Fe *3d* orbitals positioned proximate to the Fermi level (Ef), indicating enhanced catalytic capability (Figure 5e). The significant orbital overlap between Fe *3d* and NO_3_^−^ orbitals ensures stable nitrate adsorption and facilitates subsequent electrocatalytic reduction steps. Further electrostatic potential (ESP) analysis demonstrates highly positive ESP values (10.69 kcal mol^−1^) localized at Fe sites, which thermodynamically favor the adsorption of nucleophilic NO_3_^−^ anions (Figure 5f). Charge transfer analysis confirms a substantial electron donation of 0.40 e^−^ from the Fe site to the NO_3_^−^ moiety, collectively demonstrating that the Fe site serves as an active center for the electrocatalytic nitrate reduction process (Figure S50) [73].

## 5 Conclusions

In summary, we have successfully designed and constructed a series of mixed‐addenda polyoxometalate‐based metal–organic frameworks (XMOF‐V_1_, X = Fe, Co, Zn) via a molecular weaving strategy. Among them, FeMOF‐V_1_ delivers exceptional electrocatalytic nitrate‐to‐ammonia performance, achieving over 95% Faradaic efficiency across a wide voltage range of −0.6 to −1.4 V vs RHE The maximum NH_3_ yield rate of 13249 µg h^−1^ mg_cat._^−1^ (−1.4 V vs RHE) and 98.5% FE (−0.9 V vs RHE) This superior activity of FeMOF‐V_1_ stems from a synergistic interplay between the integrated P_2_W_17_V_1_ polyoxometalate, owing to its efficient proton/electron transfer, enabled by the unique electronic structure of FeMOF‐V_1_: DFT calculations reveal that the compound exhibits superior reducing ability, which is attributed to its high HOMO energy, the smallest HOMO‐LUMO energy gap and bandgap, the directional electron transfer from reduced P_2_W_17_V_1_ endow it with superior reducing ability, while PDOS and ESP analyses confirm strong Fe 3*d*–NO_3_^–^ hybridization near Ef and highly positive Fe sites (+10.69 kcal mol^−1^) facilitating NO_3_^−^ adsorption. Critical N–O bond weakening via 0.40 e^−^ charge transfer (validated by in situ FTIR detection of NO_3_/NO/*NH_2_ intermediates) and an optimized N‐end pathway (0.814 eV lower barrier than O‐end) collectively minimize energy demands, evidenced by a 0.962 eV barrier for the rate‐determining *NO_3_ → *HNO_3_ step, substantially below CoMOF‐V_1_ and ZnMOF‐V_1_ (2.026/1.291 eV). Coupled with weak H adsorption (ΔG_*H_ > ΔG_*NO3_) suppressing HER, these features enable high‐selectivity NH_3_ production. This work demonstrates the feasibility of precisely engineered mixed‐addenda POMOFs for efficient e‐NO_3_RA electrocatalysts. Our work opens the door to a vast family of high‐performance electrocatalysts, whose properties can be finely tuned through the V/W ratio in POMOFs for future applications.

## Conflicts of Interest

The authors declare no conflicts of interest.

## Supporting information




**Supporting File**: advs73570‐sup‐0001‐SuppMat.docx.

## Data Availability

The data that support the findings of this study are available from the corresponding author upon reasonable request.

## References

[1] C. Yang , Y. Zhu , J. Liu , et al., “Defect Engineering For Electrochemical Nitrogen Reduction Reaction To Ammonia,” Nano Energy 77 (2020): 105126.

[2] C. Ma , Y. Zhang , S. Yan , and B. Liu , “Carbon‐Doped Boron Nitride Nanosheets: A High‐Efficient Electrocatalyst For Ambient Nitrogen Reduction,” Applied Catalysis B: Environmental 315 (2022): 121574.

[3] Z. Gong , W. Zhong , Z. He , et al., “Regulating Surface Oxygen Species On Copper (I) Oxides Via Plasma Treatment For Effective Reduction Of Nitrate To Ammonia,” Applied Catalysis B: Environmental 305 (2022): 121021.

[4] Y. Gao , Q. Xia , L. Hao , A. W. Robertson , and Z. Sun , “Design Of Porous Core–Shell Manganese Oxides To Boost Electrocatalytic Dinitrogen Reduction,” ACS Sustainable Chemistry & Engineering 10 (2022): 1316–1322.

[5] Z. Wu , M. Karamad , X. Yong , et al., “Electrochemical Ammonia Synthesis Via Nitrate Reduction On Fe Single Atom Catalyst,” Nature Communications 12 (2021): 2870. 10.1038/s41467-021-23115-x PMC8128876 34001869

[6] M. Jiang , A. Tao , Y. Hu , et al., “Crystalline Modulation Engineering of Ru Nanoclusters for Boosting Ammonia Electrosynthesis from Dinitrogen or Nitrate,” ACS Applied Materials & Interfaces 14 (2022): 17470–17478. 35394763 10.1021/acsami.2c02048

[7] C. Wang , F. Ye , J. Shen , K. H. Xue , Y. Zhu , and C. Li , “In Situ Loading of Cu 2 O Active Sites on Island‐like Copper for Efficient Electrochemical Reduction of Nitrate to Ammonia,” ACS Applied Materials & Interfaces 14 (2022): 6680–6688. 35076198 10.1021/acsami.1c21691

[8] C. Yang , B. Huang , S. Bai , Y. Feng , Q. Shao , and X. Huang , “A Generalized Surface Chalcogenation Strategy for Boosting the Electrochemical N 2 Fixation of Metal Nanocrystals,” Advanced Materials 32 (2020): 2001267. 10.1002/adma.202001267 32390237

[9] G. Zhang , H. Xu , Y. Li , et al., “Interfacial Engineering of SeO Ligands on Tellurium Featuring Synergistic Functionalities of Bond Activation and Chemical States Buffering toward Electrocatalytic Conversion of Nitrogen to Ammonia,” Advanced Science 6 (2019): 1901627. 31637176 10.1002/advs.201901627 PMC6794632

[10] Y. Wang , H. Li , W. Zhou , X. Zhang , B. Zhang , and Y. Yu , “Structurally Disordered RuO 2 Nanosheets with Rich Oxygen Vacancies for Enhanced Nitrate Electroreduction to Ammonia,” Angewandte Chemie International Edition 61 (2022): 202202604. 10.1002/anie.202202604 35231157

[11] H. Liu , N. Guijarro , and J. Luo , “The Pitfalls In Electrocatalytic Nitrogen Reduction For Ammonia Synthesis,” Journal of Energy Chemistry 61 (2021): 149–154.

[12] Y. Yu , Q. Zhang , S. Wei , et al., “O‐Induced Diatomic Fe–Mo Mimetic Enzyme for Efficient Electrocatalytic Nitrogen Reduction at Universal pH,” Journal of the American Chemical Society 147 (2025): 28024–28034. 40719654 10.1021/jacs.5c07762

[13] S. Liu , T. Qian , M. Wang , et al., “Proton‐Filtering Covalent Organic Frameworks With Superior Nitrogen Penetration Flux Promote Ambient Ammonia Synthesis,” Nature Catalysis 4 (2021): 322–331.

[14] Z. Deng , J. Liang , Q. Liu , et al., “High‐Efficiency Ammonia Electrosynthesis On Self‐Supported Co2alo4 Nanoarray In Neutral Media By Selective Reduction Of Nitrate,” Chemical Engineering Journal 435 (2022): 135104.

[15] I. E. Khalil , C. Xue , W. Liu , et al., “The Role of Defects in Metal–Organic Frameworks for Nitrogen Reduction Reaction: When Defects Switch to Features,” Advanced Functional Materials 31 (2021): 2010052.

[16] H. Liu , S. Han , Y. Zhao , et al., “Surfactant‐Free Atomically Ultrathin Rhodium Nanosheet Nanoassemblies For Efficient Nitrogen Electroreduction,” Journal of Materials Chemistry A 6 (2018): 3211–3217.

[17] F. Chen , Z. Wu , S. Gupta , et al., “Efficient Conversion Of Low‐Concentration Nitrate Sources Into Ammonia On A Ru‐Dispersed Cu Nanowire Electrocatalyst,” Nature Nanotechnology 17 (2022): 1748–3395. 10.1038/s41565-022-01121-4 35501378

[18] Q. Yao , J. Chen , S. Xiao , Y. Zhang , and X. Zhou , “Selective Electrocatalytic Reduction of Nitrate to Ammonia with Nickel Phosphide,” ACS Applied Materials & Interfaces 13 (2021): 30458–30467. 34159788 10.1021/acsami.0c22338

[19] H. Niu , Z. Zhang , X. Wang , X. Wan , C. Shao , and Y. Gu , “Theoretical Insights into the Mechanism of Selective Nitrate‐to‐Ammonia Electroreduction on Single‐Atom Catalysts,” Advanced Functional Materials 31 (2020): 2008533.

[20] X. Zhao , G. Hu , F. Tan , et al., “Copper Confined In Vesicle‐Like Bcn Cavities Promotes Electrochemical Reduction Of Nitrate To Ammonia In Water,” Journal of Materials Chemistry A 9 (2021): 23675–23686.

[21] P. Gao , Z. Xue , S. Zhang , et al., “Schottky Barrier‐Induced Surface Electric Field Boosts Universal Reduction of NO x− in Water to Ammonia,” Angewandte Chemie International Edition 60 (2021): 20711–20716. 34313361 10.1002/anie.202107858

[22] Y. Wang , L. Zhang , Y. Niu , et al., “Boosting Nh 3 Production From Nitrate Electroreduction Via Electronic Structure Engineering Of Fe 3 C Nanoflakes,” Green Chemistry 23 (2021): 7594–7608.

[23] Y. Wang , Y. Yu , R. Jia , C. Zhang , and B. Zhang , “Electrochemical Synthesis Of Nitric Acid From Air And Ammonia Through Waste Utilization,” National Science Review 6 (2019): 730–738. 34691928 10.1093/nsr/nwz019 PMC8291439

[24] X. Wei , X. Wen , Y. Liu , et al., “Oxygen Vacancy‐Mediated Selective C–N Coupling toward Electrocatalytic Urea Synthesis,” Journal of the American Chemical Society 144 (2022): 11530–11535. 35748598 10.1021/jacs.2c03452

[25] X. Deng , Y. Yang , L. Wang , X. Fu , and J. Luo , “Metallic Co Nanoarray Catalyzes Selective Nh3 Production From Electrochemical Nitrate Reduction At Current Densities Exceeding 2 A Cm− 2,” Advanced Science 3 (2021): 2004523. 10.1002/advs.202004523 PMC8025016 33854903

[26] Q. Hu , Y. Qin , X. Wang , et al., “Accelerating the Activation of NOx− on Ru Nanoparticles for Ammonia Production by Tuning Their Electron Deficiency,” CCS Chemistry 3 (2021): 2092–2103.

[27] J. Yuan , Z. Xing , Y. Tang , and C. Liu , “Tuning the Oxidation State of Cu Electrodes for Selective Electrosynthesis of Ammonia from Nitrate,” ACS Applied Materials & Interfaces 13 (2021): 52469–52478. 34723479 10.1021/acsami.1c10946

[28] Z. Ge , T. Wang , Y. Ding , et al., “Interfacial Engineering Enhances the Electroactivity of Frame‐Like Concave RhCu Bimetallic Nanocubes for Nitrate Reduction,” Advanced Energy Materials 12: 2103916.

[29] M. Jiang , J. Su , X. Song , et al., “Interfacial Reduction Nucleation Of Noble Metal Nanodots On Redox‐Active Metal–Organic Frameworks For High‐Efficiency Electrocatalytic Conversion Of Nitrate To Ammonia,” Nano Letters 22 (2022): 2529–2537. 35266387 10.1021/acs.nanolett.2c00446

[30] Z. Yan , W. Gao , C. Zhong , Q. Jiao , S. Tian , and J. Liu , “Regulating Spin State Of Fe(Iii) By The Mo Single Atom Anchored In The (001) Crystal Face Of Α‐Fe2o3 To Achieve Efficient Electrocatalytic Nitrate To Synthesize Ammonia,” Applied Catalysis B: Environment and Energy 366 (2025): 125008.

[31] S. Yin , R. Cao , Y. Han , et al., “Electrocatalysts With Atomic‐Level Site For Nitrate Reduction To Ammonia,” Journal of Energy Chemistry 96 (2024): 642–668.

[32] J. Mu , D. Wang , S. Zhou , et al., “Max‐Derived B‐Doped Mo 1.33 C Mxene For Ambient Electrocatalytic Conversion Of Nitrate To Ammonia,” Journal of Materials Chemistry A 12 (2024): 18082.

[33] G. Zhu , W. Bao , M. Xie , et al., “Accelerating Tandem Electroreduction of Nitrate to Ammonia via Multi‐Site Synergy in Mesoporous Carbon‐Supported High‐Entropy Intermetallics,” Advanced Materials 37 (2025): 2413560. 10.1002/adma.202413560 39648538

[34] J. Li , R. Valenza , and S. Haussener , “In Situ Synthesis of Cu x O/N Doped Graphdiyne with Pyridine N Configuration for Ammonia Production via Nitrate Reduction,” Small 20 (2024): 2310467. 10.1002/smll.202310467 38552223

[35] J. Ni , J. Yan , F. Li , et al., “Atomic Co─P Catalytic Pair Drives Efficient Electrochemical Nitrate Reduction to Ammonia,” Advanced Energy Materials 14 (2024): 2400065.

[36] P. Zhai , C. Wang , Y. Li , et al., “Molecular Engineering of Hydrogen‐Bonded Organic Framework for Enhanced Nitrate Electroreduction to Ammonia,” Nano Letters 24 (2024): 8687–8695. 38973752 10.1021/acs.nanolett.4c02030

[37] J. Hu , H. Huang , M. Yu , S. Wang , and J. Li , “Electron Engineering Of Nickel Phosphide For Niδ+ In Electrochemical Nitrate Reduction To Ammonia,” Nano Research 17 (2024): 4864–4871.

[38] B. Zhang , Z. Dai , Y. Chen , et al., “Defect‐Induced Triple Synergistic Modulation In Copper For Superior Electrochemical Ammonia Production Across Broad Nitrate Concentrations,” Nature Communications 15 (2024): 2816. 10.1038/s41467-024-47025-w PMC10984973 38561364

[39] H. Luo , S. Li , Z. Wu , et al., “Relay Catalysis of Fe and Co with Multi‐Active Sites for Specialized Division of Labor in Electrocatalytic Nitrate Reduction Reaction,” Advanced Functional Materials 34 (2024): 2403838.

[40] Y. Zou , X. Wang , F. Ning , J. Yi , and Y. Liu , “Implanting MWCNTs in BiCu‐MOFs to enhance electrocatalytic CO2 reduction to formate,” Separation and Purification Technology 317 (2023): 123806.

[41] V. Day and W. Klemperer , “Metal Oxide Chemistry in Solution: The Early Transition Metal Polyoxoanions,” Science 228 (1985): 533–541. 17736064 10.1126/science.228.4699.533

[42] M. Pope , in “Heteropoly and Isopoly Oxometalates,” (Berlin: Springer‐Verlag, 1983), 46.

[43] S. Wang and G. Yang , “Recent Advances in Polyoxometalate‐Catalyzed Reactions,” Chemical Reviews 115 (2015): 4893–4962. 25965251 10.1021/cr500390v

[44] K. Moltved and K. Kepp , “The Chemical Bond between Transition Metals and Oxygen: Electronegativity, d‐Orbital Effects, and Oxophilicity as Descriptors of Metal–Oxygen Interactions,” The Journal of Physical Chemistry C 123 (2019): 18432–18444.

[45] M. Lu , M. Zhang , J. Liu , et al., “Confining and Highly Dispersing Single Polyoxometalate Clusters in Covalent Organic Frameworks by Covalent Linkages for CO 2 Photoreduction,” Journal of the American Chemical Society 144 (2022): 1861–1871. 35050618 10.1021/jacs.1c11987

[46] J. Li , M. Yang , A. Tian , X. Cao , J. Ying , and X. Wang , “Direct Electron Transfer From Electron‐Reservoir Sandwich‐Type Polyoxometalate To Co2ni2‐Cluster Via Water‐Assisted Proton Channels And Tandem Effects For Efficient Nitrate‐To‐Ammonia Electrocatalysis,” Chemical Engineering Journal 521 (2025): 166862.

[47] B. Sun , D. Wang , Y. Jiang , et al., “Cyclodextrin Metal–Organic Framework Functionalized Carbon Materials with Optimized Interface Electronics and Selective Supramolecular Channels for High‐Performance Lithium–Sulfur Batteries,” Advanced Materials 36 (2024): 2415633. 10.1002/adma.202415633 39501988

[48] K. Yue , R. Lu , M. Gao , et al., “Polyoxometalated Metal‐Organic Framework Superstructure For Stable Water Oxidation,” Science 388 (2025): 430–436. 40273253 10.1126/science.ads1466

[49] C. Cheng , X. Yi , W. Chen , R. Sang , and L. Xu , “Electron‐Rich MoIV3‐Polyoxomolybdates Resembling the Paratungstic Archetype,” Chemistry–A European Journal 29 (2023): 202300043. 10.1002/chem.202300043 37062700

[50] M. Sun , Y. Wang , W. He , et al., “Efficient Electron Transfer from Electron‐Sponge Polyoxometalate to Single‐Metal Site Metal–Organic Frameworks for Highly Selective Electroreduction of Carbon Dioxide,” Small 17 (2021): 2100762. 10.1002/smll.202100762 33817965

[51] M. Lan , Y. Li , C. Wang , et al., “Multi‐Channel Electron Transfer Induced By Polyvanadate In Metal‐Organic Framework For Boosted Peroxymonosulfate Activation,” Nature Communications 15 (2024): 7208. 10.1038/s41467-024-51525-0 PMC11341957 39174565

[52] M. Yang , X. Wang , C. Gómez‐García , et al., “Efficient Electron Transfer from an Electron‐Reservoir Polyoxometalate to Dual‐Metal‐Site Metal‐Organic Frameworks for Highly Efficient Electroreduction of Nitrogen,” Advanced Functional Materials 33 (2023): 2214495.

[53] X. Yang , T. Liu , R. Li , et al., “Host–Guest Molecular Interaction Enabled Separation of Large‐Diameter Semiconducting Single‐Walled Carbon Nanotubes,” Journal of the American Chemical Society 143 (2021): 10120–10130. 34105955 10.1021/jacs.1c02245

[54] Y. Tong , H. Guo , D. Liu , et al., “Vacancy Engineering of Iron‐Doped W 18 O 49 Nanoreactors for Low‐Barrier Electrochemical Nitrogen Reduction,” Angewandte Chemie International Edition 59 (2020): 7356–7361. 32084292 10.1002/anie.202002029

[55] F. Dhifallah , M. Belkhiria , L. Parent , N. Leclerc , and E. Cadot , “A Series of Octahedral First‐Row Transition‐Metal Ion Complexes Templated by Wells–Dawson Polyoxometalates: Synthesis, Crystal Structure, Spectroscopic, and Thermal Characterizations, and Electrochemical Properties,” Inorganic Chemistry 57 (2018): 11909–11919. 30198715 10.1021/acs.inorgchem.8b01207

[56] H. Fan , H. Li , K. Huang , et al., “Metastable Marcasite‐FeS 2 as a New Anode Material for Lithium Ion Batteries: CNFs‐Improved Lithiation/Delithiation Reversibility and Li‐Storage Properties,” ACS Applied Materials & Interfaces 9 (2017): 10708–10716. 28263060 10.1021/acsami.7b00578

[57] B. Hou , Y. Wang , J. Guo , et al., “A Scalable Strategy To Develop Advanced Anode for Sodium‐Ion Batteries: Commercial Fe 3 O 4 ‐Derived Fe 3 O 4 @FeS with Superior Full‐Cell Performance,” ACS Applied Materials & Interfaces 10 (2018): 3581–3589. 29303243 10.1021/acsami.7b16580

[58] Q. Wang , W. Zhang , C. Guo , Y. Liu , C. Wang , and Z. Guo , “In Situ Construction of 3D Interconnected FeS@Fe 3 C@Graphitic Carbon Networks for High‐Performance Sodium‐Ion Batteries,” Advanced Functional Materials 27 (2017): 1703390.

[59] G. Chen , Y. Yuan , H. Jiang , et al., “Electrochemical Reduction Of Nitrate To Ammonia Via Direct Eight‐Electron Transfer Using A Copper–Molecular Solid Catalyst,” Nature Energy 5 (2020): 605–613.

[60] D. Garg , L. Mallick , A. Kundu , and B. Chakraborty , “In Situ Spectroscopic Probing of the Hydroxylamine Pathway of Electrocatalytic Nitrate Reduction on Iron‐Oxy‐Hydroxide,” Small 21 (2025): 2412606. 10.1002/smll.202412606 39807688

[61] Z. Tang , Z. Bai , X. Li , L. Ding , B. Zhang , and X. Chang , “Chloride‐Derived Bimetallic Cu‐Fe Nanoparticles For High‐Selective Nitrate‐To‐Ammonia Electrochemical Catalysis,” Processes 10 (2022): 751.

[62] X. Wang , L. Dong , M. Qiao , et al., “Exploring the Performance Improvement of the Oxygen Evolution Reaction in a Stable Bimetal–Organic Framework System,” Angewandte Chemie International Edition 57 (2018): 9660–9664. 29660248 10.1002/anie.201803587

[63] K. Chu , Y. Cheng , Q. Li , Y. Liu , and Y. Tian , “Fe‐Doping Induced Morphological Changes, Oxygen Vacancies And Ce 3+ –Ce 3+ Pairs In Ceo 2 For Promoting Electrocatalytic Nitrogen Fixation,” Journal of Materials Chemistry A 8 (2020): 5865–5873.

[64] X. Lv , X. Liu , L. Gao , Y. Liu , and Z. Yuan , “Iron‐Doped Titanium Dioxide Hollow Nanospheres For Efficient Nitrogen Fixation And Zn–N 2 Aqueous Batteries,” Journal of Materials Chemistry A 9 (2021): 4026–4035.

[65] Q. Li , Y. Zhang , X. Wang , and Y. Yang , “Dual Interface‐Engineered Tin Heterostructure for Enhanced Ambient Ammonia Electrosynthesis,” ACS Applied Materials & Interfaces 13 (2021): 15270–15278. 33769776 10.1021/acsami.1c01160

[66] J. Szanyi , J. Kwak , H. Zhu , and C. Peden , “Characterization Of Cu‐Ssz‐13 Nh3 Scr Catalysts: An In Situ Ftir Study,” Physical Chemistry Chemical Physics 15 (2013): 2368–2380. 23301245 10.1039/c2cp43467a

[67] A. Kunov‐Kruse , P. Thomassen , A. Riisager , S. Mossin , and R. Fehrmann , “Absorption and Oxidation of Nitrogen Oxide in Ionic Liquids,” Chemistry—A European Journal 22 (2016): 11745–11755. 27384885 10.1002/chem.201601166

[68] K. Ataka , T. Yotsuyanagi , and M. Osawa , “Potential‐Dependent Reorientation of Water Molecules at an Electrode/Electrolyte Interface Studied by Surface‐Enhanced Infrared Absorption Spectroscopy,” The Journal of Physical Chemistry 100 (1996): 10664–10672.

[69] M. Mihaylov and K. Hadjiivanov , “FTIR Study of CO and NO Adsorption and Coadsorption on Ni‐ZSM‐5 and Ni/SiO 2,” Langmuir 18 (2002): 4376–4383.

[70] J. Ren , C. Wan , T. Pei , X. Lv , and Z. Yuan , “Promotion Of Electrocatalytic Nitrogen Reduction Reaction On N‐Doped Porous Carbon With Secondary Heteroatoms,” Applied Catalysis B: Environmental 266 (2020): 118633.

[71] P. Song , L. Kang , H. Wang , R. Guo , and R. Wang , “Nitrogen (N), Phosphorus (P)‐Codoped Porous Carbon as a Metal‐Free Electrocatalyst for N 2 Reduction under Ambient Conditions,” ACS Applied Materials & Interfaces 11 (2019): 12408–12414. 30859808 10.1021/acsami.8b20472

[72] Y. Zhao , Z. Shang , M. Feng , et al., “Oriented Assembly of 2D Metal‐Pyridylporphyrinic Framework to Regulate the Redox Kinetics in Li−S Batteries,” Advanced Materials 37 (2025): 2501869. 10.1002/adma.202501869 40099643

[73] M. Wang , Y. Meng , W. Xu , et al., “Square‐Planar Tetranuclear Cluster‐Based High‐Symmetry Coordination Metal–Organic Polymers for Efficient Electrochemical Nitrate Reduction to Ammonia,” Journal of the American Chemical Society 147 (2025): 18327–18337. 40367342 10.1021/jacs.5c06650

