# Supplementary material for: Molecular Weaving of Mixed‐Addenda Polyoxometalates into MOF Nanochannels as Electron‐Buffering Reservoirs for Enhanced Nitrate‐to‐Ammonia Electrocatalysis
Source: Adv Sci (Weinh). 2025 Dec 25;13(14):e21317. doi: 10.1002/advs.202521317 (PMC12970160; doi:10.1002/advs.202521317)
Supplement: Supplementary file 1 — Supporting File: advs73570‐sup‐0001‐SuppMat.docx. [file ADVS-13-e21317-s001.docx]

**Supporting Information**

**Molecular Weaving of Mixed-Addenda Polyoxometalates into MOF Nanochannels as Electron-Buffering Reservoirs for Enhanced Nitrate-to-Ammonia Electrocatalysis**

*Gang Li, Xinming Wang,* Haijun Pang, Huiyuan Ma**

G. Li, X. Wang, H. Pang, Prof. H. Ma

School of Materials Science and Chemical Engineering, Harbin University of Science and Technology, Harbin, 150040, P. R. China

*Corresponding authors: [wangxinming20@126.com](mailto:wangxinming20@126.com) (X. M. Wang); mahy017@163.com (H. Y. Ma)

**Experimental Section**

**1. Materials.**

The precursor K_7_[P_2_W_17_V_1_O_62_]·18H_2_O (P_2_W_17_V_1_) were prepared in accordance with methods [1]. 1,4-Di(pyridin-4-yl)benzene (C_16_H_12_N_2_) was obtained from Macklin. Nafion per-fluorinated resin solution, Hydrogen peroxide (H_2_O_2_, AR), Sulfuric acid (H_2_SO_4_, AR), Hydrochloric acid (HCl, AR), Potassium sodium tartrate tetrahydrate (C_4_H_12_KNaO_10_, AR), Ammonium chloride (NH_4_Cl, AR), Iron(III) chloride hexahydrate (FeCl_3_·6H_2_O, AR), Cobalt(II) chloride hexahydrate (CoCl_2_·6H_2_O, AR), Zinc(II) chloride (ZnCl_2_, AR), Oxalic acid (H_2_C_2_O_4_, AR), Nitric acid (HNO_3_, AR), Sodium hydroxide (NaOH, AR), Ethanol (C_2_H_5_OH, AR), Sodium citrate dihydrate (C_6_H_5_Na_3_O_7_·2H_2_O), Salicylic acid (C_7_H_6_O_3_), Sodium penta(cyano)nitroferrate(III) dihydrate (Na_2_[Fe(CN)_5_NO]·2H_2_O), Sodium hypochlorite solution (NaClO, ρCl≥5.0%), Acetylene black (shorted as AB) was obtained from Beijing Chemical Works, China. Dimethyl sulfoxide-*d_6_* (DMSO-*d_6_*, deuterium for 99.9%) was obtained from Alfa Aesar, USA. Argon (Ar, high-purity, 99.9999%) was purchased from Qing Hua Gas Co., China. The K^15^NO_3_ isotope with the N enrichment of 10% was obtained from aladdin. Ultrapure water (18.2 MΩ cm^−1^) was used in all experiments. All these chemicals were used as received without further purification.

**2. Characterization**

The Fourier Transform Infrared Spectroscopy (FTIR) spectra were recorded for KBr pellets in the range of 4000-400 cm^−1^ with a Bruker OPTIK GmbH-Tensor II spectrometer (KBr disk). The powder X-ray diffraction (PXRD) patterns were obtained with a Rigaku D/max 2500 V PC diffractometer with Cu-Kα radiation, and the scanning rate is 5° s^–1^, 2θ ranging from 5 to 50°. The X-ray photoelectron spectroscopy (XPS) measurements were performed by a Thermo ESCALAB 250Xi spectrometer with monochromated Al Kα radiation (hγ = 1486.6 eV). All XPS spectra were calibrated with respect to the C 1s peak at 284.6 eV. The morphology and microstructure of the samples were characterized by field emission scanning electron microscopy (SEM) (Hitachi, SU8000). Energy dispersive spectroscopy (EDS) data was collected with an ensemble measurement in the FE-SEM. The content of W, V, and Fe/Co/Zn in the electrolyte were determined by inductively coupled plasma optical emission spectrometer (ICP-OES, Aglient 5110). ^1^H nuclear magnetic resonance (^1^H NMR) experiments were carried out at 303 K for 5% w/v sample solution in DMSO-*d*_6_ using Bruker Avance NEO 300. The spectral windows were set to 12.5 kHz (25 ppm), a total of 16 scans were recorded, a π/2 pulse length of 11.6 μs and 64 K data points with 3 s recycle delay for each sample. After e-NO_3_RA test, the electrolyte was subjected to distillation treatment, and the steam including NH_3_ and H_2_O was condensed into a container. Solid product was then obtained for NMR examination by freeze-drying of above solution. UV-visible spectra were measured on a U-3900 UV–vis spectrophotometer (Hitachi, Japan). All the electrochemical tests were carried out in a three-electrode testing system (CHI 760E electrochemical workstation, Chenhua, Shanghai). A conventional three-electrode system was used, the catalyst-modified carbon paper electrode (denoted as CPE, 1 cm × 1 cm) as a working electrode, a commercial Ag/AgCl as reference electrode and a graphite rod as counter electrode. Rotating Disk Electrode (RDE) Experiment: RDE experiments were performed in 0.05 mol L^–1^ Na_2_SO_4_ and 0.1 mol L^–1^ KNO_3_. The electrochemical rate constants (K) and kinetic current (*i*_k_) were determined using a Koutecky–Levich (K–L) plot using Equations (1) and (2)

1/*i*_c_ = 1/*i*_k_ + [1/0.62*nFAD*^2/3^υ^–1/6^*C*_0_)]ω^–1/2^ (1)

*i*_k_ = *nFAkC*_0_ (2)

where *n* is the number of transferred electrons for nitrate to ammonia; *F* is the Faraday constant (96485 C mol^–1^); *A* is the surface area of the electrode (1 cm^2^); *D* is the diffusion coefficient of [NO_3_]^–^ (2 × 10^–5^ cm^2^ s^–1^); *v* is the kinematic viscosity (0.0088 cm^2^ s^–1^); *i*_C_ is the current at a defined working potential (V vs. RHE); *i*_k_ is the kinetic current; *C*_0_ is the initial concentration of electrolyte, and *k* is the electrochemical rate constant. Steady-state voltammograms were recordedat different rotating rates (ω) (rotating rates: 1800, 2200, 2600, 3000, and 3400 rpm) using the cell setup mentioned above and FeMOF-V_1_ deposited on the working electrode. The reciprocal of kinetic current (*i*_k_) can be derived from the intercept with the y-axis, as shown by Eqs.1 and 2. Equation (2) was used to obtain the electrochemical rate constant once the kinetic current was determined.

**3. Synthesis of catalysts.**

**3.1 Synthesis of [Fe_9_(DPYB)_9_(OX)_6_(H_2_O)_6_][P_2_W_17_V_1_O_62_] (FeMOF-V_1_)**

A mixture of K_7_[P_2_W_17_V_1_O_62_]·18H_2_O (100 mg, 0.02 mmol), DPYB (20 mg, 0.1 mmol), FeCl_3_·6H_2_O (108 mg, 0.4 mmol) and oxalic acid (OX) (50 mg, 0.56 mmol) were stirred for 1 h in air at room temperature. The pH value was adjusted to about 3.8 with 1.0 M mol HCl or NaOH and then the suspension was transferred to a 25 mL Teflon lined autoclave and kept at 160 °C for 4 days. After slow cooling to room temperature, hexahedron crystals of FeMOF-V_1_ were obtained (yield 57% based on W).

**3.2 Synthesis of [Co_9_(DPYB)_9_(OX)_6_(H_2_O)_6_][P_2_W_17_V_1_O_62_] (CoMOF-V_1_)**

CoMOF-V_1_ was prepared by an analogous method to FeMOF-V_1_ but with CoCl_2_·6H_2_O in place of FeCl_3_·6H_2_O. Hexahedron crystals of CoMOF-V_1_ were obtained in a yield of 60% (based on W).

**3.3 Synthesis of [Zn_9_(DPYB)_9_(OX)_6_(H_2_O)_6_][P_2_W_17_V_1_O_62_] (ZnMOF-V_1_)**

ZnMOF-V_1_ was prepared following the procedure for FeMOF-V_1_ but with ZnCl_2_ replacing FeCl_3_·6H_2_O. The reaction afforded hexahedral crystals of ZnMOF-V_1_ in a yield of 60% (based on W).

**3.4 Synthesis of {[Fe(DPYB)(OX)]_2_(DPYB)_0.5_} (FeMOF).**

FeMOF was prepared as FeMOF-V1 without adding P_2_W_17_V_1_. Orange-red crystals of FeMOF were obtained (yield 21 % based on Fe).

**3.5 Synthesis of {[Co(DPYB)(OX)]_2_(DPYB)_0.5_} (CoMOF).**

CoMOF was prepared as FeMOF adding CoCl_2_·6H_2_O instead of FeCl_3_·6H_2_O. Pale-yellow irregular crystals of CoMOF-P_2_W_17_V_1_ were obtained (yield 17 % based on Fe).

**3.6 Synthesis of {[Zn(DPYB)(OX)]_2_(DPYB)_0.5_} (ZnMOF).**

ZnMOF was prepared as FeMOF adding ZnCl_2_ instead of FeCl_3_·6H_2_O. Light-white crystals of ZnMOF were obtained (yield 29 % based on Zn).

**3.7 Preparations of P_2_W_17_V_1_@NBu_4_Br sample.**

A solution of 0.1 mmol of P_2_W_17_V_1_ in 20 mL of distilled water was added, under stirring, to a solution containing 0.24 mmol of tetrabutylammonium bromide (NBu_4_Br) in 5 mL of distilled water under stirring. The resulting cloudy suspension was filtrated, and the precipitate was collected, washed with distilled water three times, and dried in the oven at 60 °C for 2 h to obtain P_2_W_17_V_1_@NBu_4_Br.

**4. Electrochemical Measurements**

e-NO_3_RA experiments were performed in a typical H-cell arrangement separated by a Nafion 211 membrane at room temperature. Before e-NO_3_RA test, Nafion membrane was protonated by first boiling in water for 1 h, then in H_2_O_2_ for 1 h, then in water for another hour, followed by 3 h in 0.5 M H_2_SO_4_, and finally for 6 h in water. All steps were performed at 80 °C. The electrochemical measurements were conducted by a CHI 760 electrochemical analyzer (Shanghai, Chenhua Co., China) in a three-S5 electrode cell containing electrolyte. Typically, XMOF-V_1_ as working electrode, an Ag/AgCl (in saturated KCl electrolyte) electrode was used as the reference electrode and a graphite rod as counter electrode. The potentials reported in this work were converted to RHE scale via calibration with the following equation: E (νs. RHE) = E (νs. Ag/AgCl) + 0.197 + 0.059 × pH, and the polarization curves were the steady-state ones after several cycles. The presented current density referred to the geometrical area of the CP. Before all measurements, high-purity Ar was first bubbled into the cathode compartment for at least 30 min with a constant flow rate of 150 mL min^–1^ to remove air in electrolyte and reactor. For e-NO_3_RA tests, a potentiostatic test was conducted for 1 h in a 0.05 M Na_2_SO_4_ (pH = 7) with 0.1 M KNO_3_ electrolytes (50 mL) at the ambient conditions of 298 K and 1 bar. Linear sweep voltammetry (LSV) measurements of the catalysts were conducted in 0.05 M Na_2_SO_4_ with and without 0.1 M KNO_3_ electrolytes, with a scan rate of 5.0 mV s^–1^. All polarization curves were obtained without current resistance (iR) compensation. Electrochemical impedance spectroscopy (EIS) measurements were carried out from 0.1 Hz to 1000 kHz with an amplitude of 10 mV at the open-circuit voltage. A volume of 5 mg electrocatalyst, 5 mg acetylene black (AB), 490 µL isopropyl alcohol, 1000 µL ethanol and 10 µL Nafion solution (0.1 wt% water solution) are grounded to form uniform catalyst ink. After sonication for 30 min, the catalyst ink was dropped directly onto a carbon paper (1 cm × 1 cm) with a catalyst loading density of ~0.33 mg cm^−2^ and dried.

**4.1 Procedures for the Determination of NH_3_ or NO_2_**

**4.1.1 The indophenol blue method.**

2 mL electrolyte was removed from the cathodic chamber and mixed with oxidizing solution containing 2 mL NaOH solution (1.0 M) containing salicylic acid (5 wt%), sodium citrate (5 wt%), 1 mL of NaClO (0.05 M), and 0.2 mL of 1 wt % Na_2_[Fe(NO)(CN)_5_] for 2 h. The solution was measured with UV–vis absorption spectrum at 655 nm. Calibration curve of NH_3_ in electrolyte solution was plotted using a series of different concentration of standard ammonia stock solution diluted by 0.05 M H_2_SO_4_. The linear relationship is y = 0.425x – 0.01226, R^2^ = 0.999 in acid electrolyte, respectively.


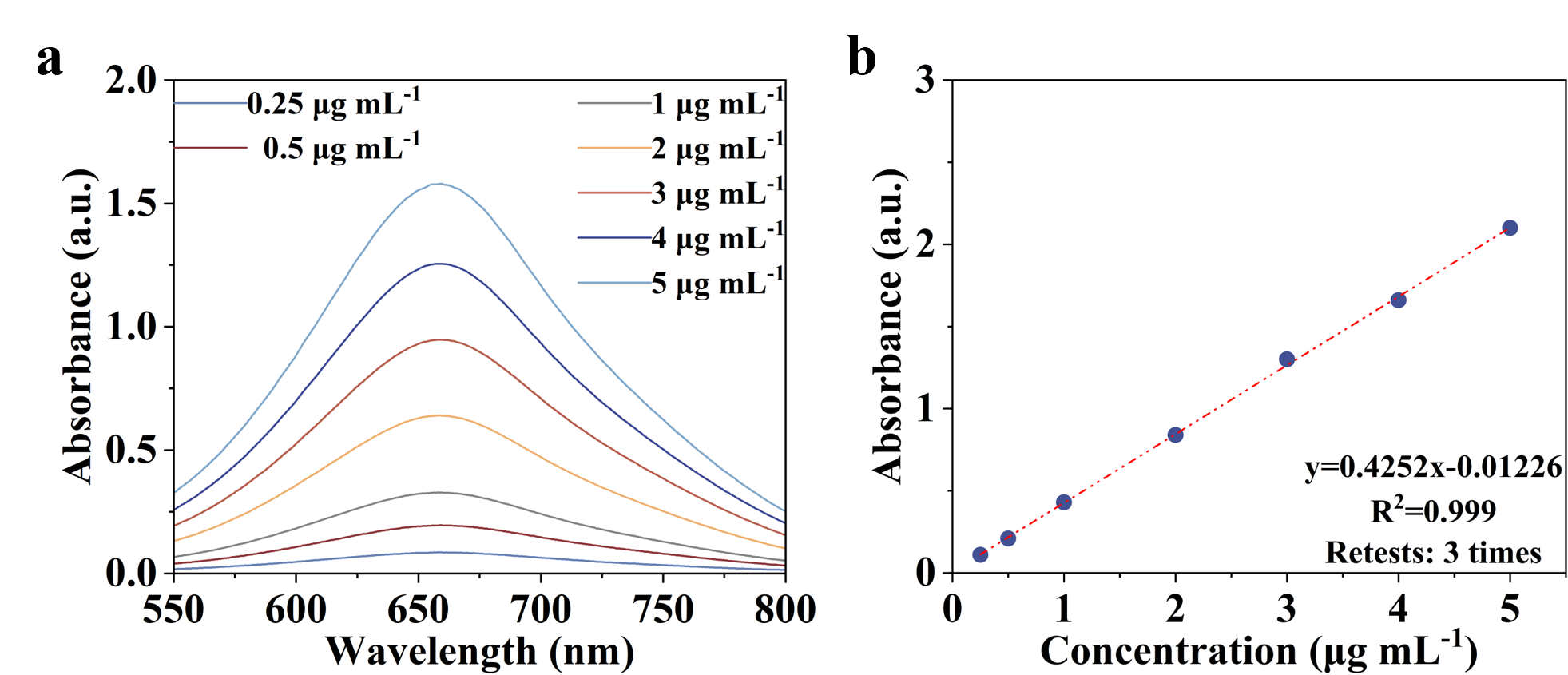


Figure S31. (a) UV-vis spectra of NH_3_ solution with different concentrations in 0.1 M KNO_3_ and (b) corresponding liner fitting between absorbance and NH_3_ concentration.

**4.1.2 Determination of NO_2_^–^**

The NO_2_^–^ concentration was analyzed using the Griess test.[52] The Griess reagent was prepared by dissolving 0.1 g of N-(1naphthyl)ethyldiamine dihydrochloride, 1.0 g of sulfonamide and 2.94 mL of H_3_PO_4_ in 50 mL of deionized H_2_O. In a typical colorimetric assay, the nitrite-containing solution (1.0 mL) was mixed with the Griess reagent (1.0 mL) and H_2_O (2 mL), and allowed to react at room temperature for 10 min, in which the sulfonamide reacts with the NO_2_^–^ to form a diazonium salt and then further reacts with the amine to form an azodye (magenta). The absorbance at ca. λ = 540 nm was measured by UV-Vis spectroscopy and the NO_2_^–^ concentration was calculated. The linear relationship is y = 0.475x – 0.08167, R^2^ = 0.999 in acid electrolyte, respectively.


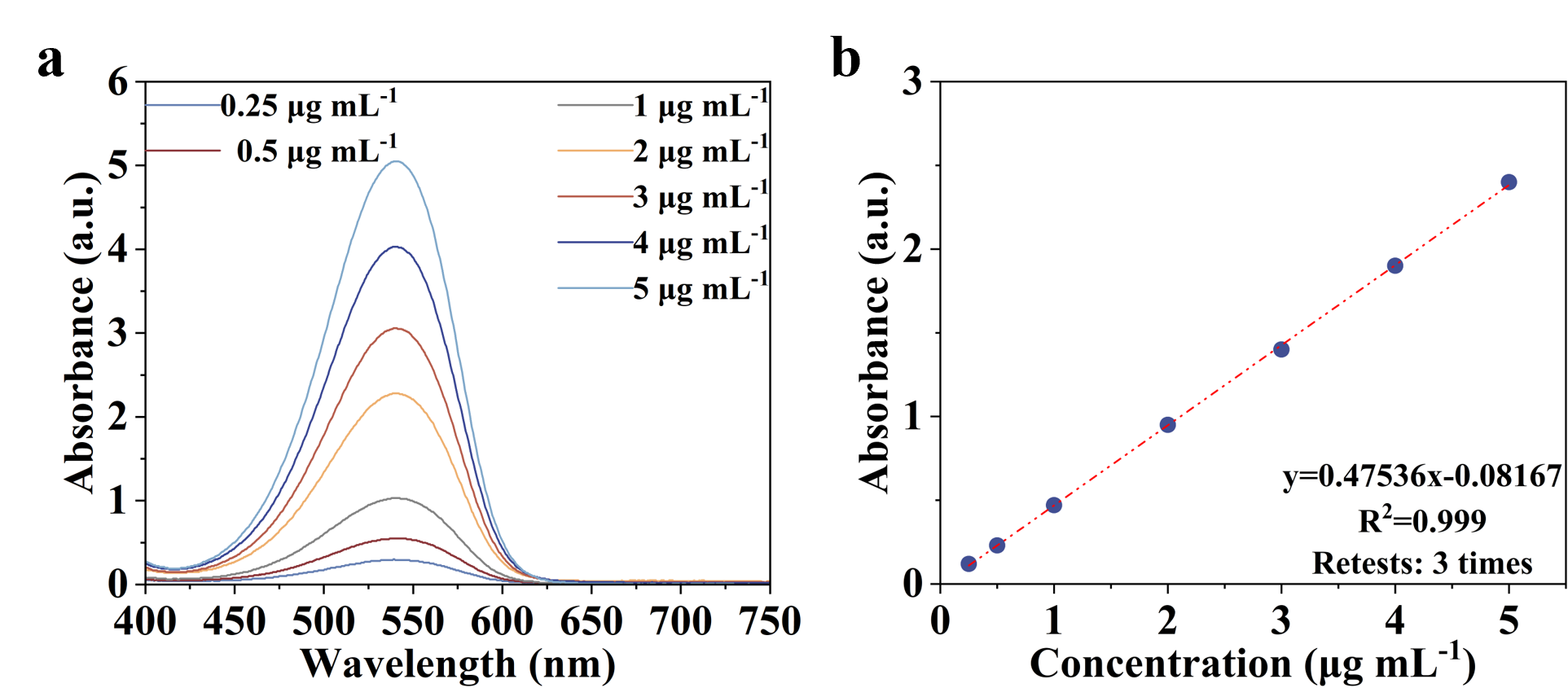


Figure S31. (a) UV-vis spectra of NO_2_^–^ solution with different concentrations in 0.1 M KNO_3_ and (b) corresponding liner fitting between absorbance and NO_2_^–^ concentration.

**4.2 Determination of FE and NH_3_ yield rate**

The total amount of NH_3_ produced was measured using colorimetric methods.

Assuming three electrons were needed to produce one NH_3_ molecule, the Faradaic efficiency could be calculated as follows:

Faradaic efficiency (FE) = 8 F × (c(NH_3_) × V)/(17 × Q)

where F is the Faraday constant, Q is the charge accumulated *via* 2 h electrocatalysis, and V is the volume of the electrolyte (50 mL).

The ammonia yield rate was calculated using the following equation:

Ammonia yield rate = (c(NH_3_) × V)/(t × m_cat._)

where c(NH_3_) is the concentration of measured NH_3_, V is the volume of the electrolyte (50 mL), t is the reduction reaction time (2 h) and m_cat._ is the loaded quality of catalyst.

**4.3 The control experiments of the K^15^NO_3_ isotopic measurements to prove the nitrogen source**

The control experiments of the K^15^NO_3_ isotopic measurements to prove the nitrogen source The ^15^N isotopic measurements were performed using the K^15^NO_3_ isotope with the ^15^N (enrichment of ＞ 99%) to clarify the nitrogen origination of ammonia. Before the electrochemical reduction procedure, the electrolyte (pH = 7 0.1 M KNO_3_) was purged with high-purity Ar to remove the N_2_ from solution. After 12 h electrolysis at –0.9 V νs. RHE, the 10 mL of the electrolyte was taken out and adjusted to pH = 2. The analysis of ^15^NH_3_ product was conducted by the ^1^H nuclear magnetic resonance with water suppression (^1^H NMR, Bruker Avance NEO 300).

**5. Computational Details.**

**First principles calculations.**

DFT calculations were performed with projector-augmented wave (PAW) methods [2]. The exchange andcorrelation energies were described by the generalized gradient approximation (GGA) with Perdew-Burke-Ernzerhof (PBE) functional [3, 4]. A planewave, cut-off energy of 450 eV was set. During geometric optimization, the total energy and the force on eachrelaxed atom were converged to 10^–5^ eV and –0.02 eV/Å, respectively. A 15 Å vacuum layer in the c-axis was set to obviateinteractions between structural periods.

ΔG = ΔE + ZPE

where ΔE is the single-point energy, ZPE is the zero-point energy.

DFT calculations were performed using the DMOL3 code with numerical atomic orbital basis sets. The exchange and correlation energies were treated within the generalized gradient approximation (GGA) using the Perdew-Burke-Ernzerhof (PBE) functional. A double numerical basis set with polarization functions (DNP) was employed, and the self-consistent field (SCF) tolerance was set to 10^–5^ eV. The electrostatic potential (ESP) was computed from the converged electron density.

**Supplementary Figures and Tables**


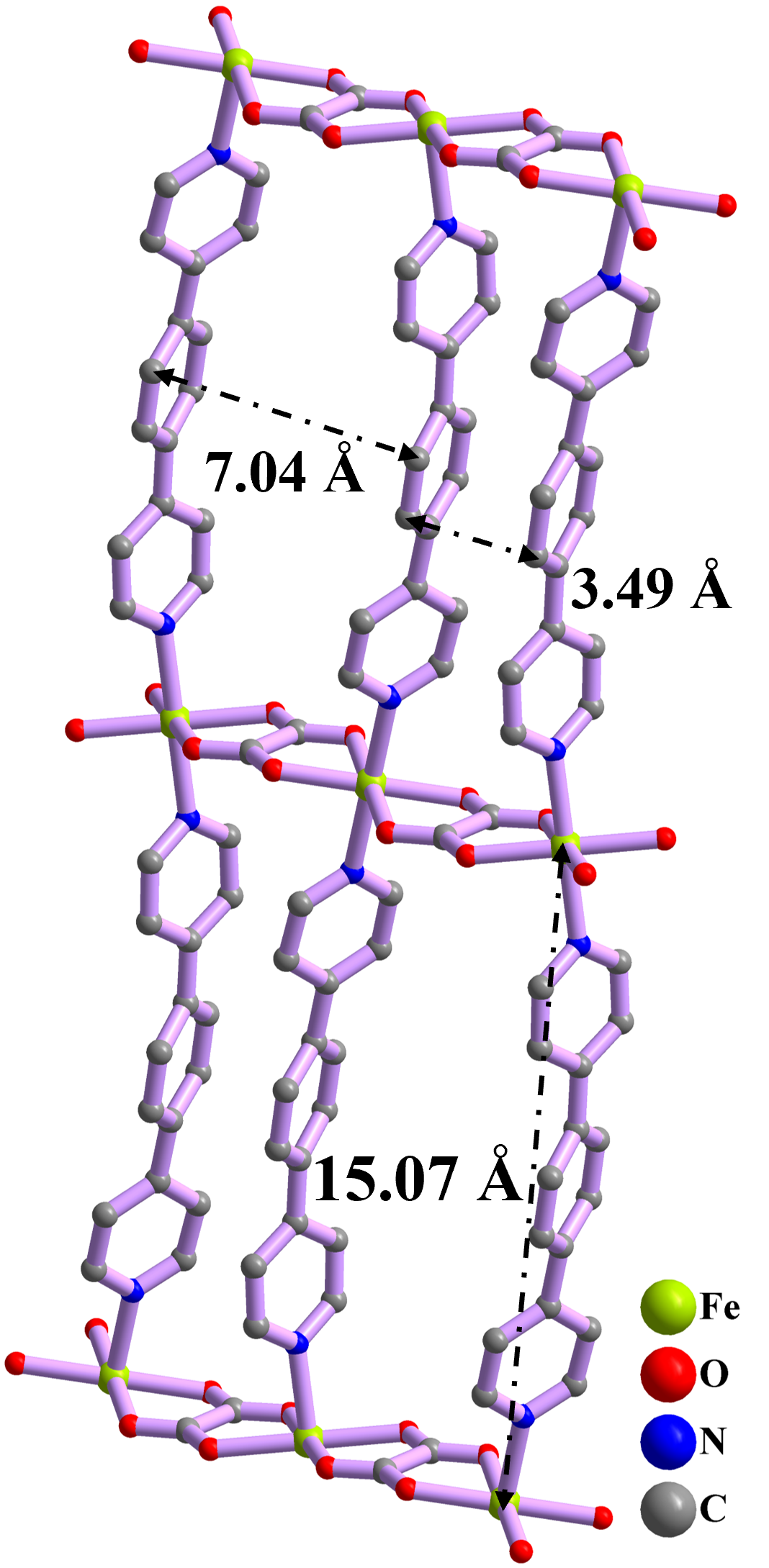


Figure S1. View of FeMOF, showing the dimensions of the rectangular channels.


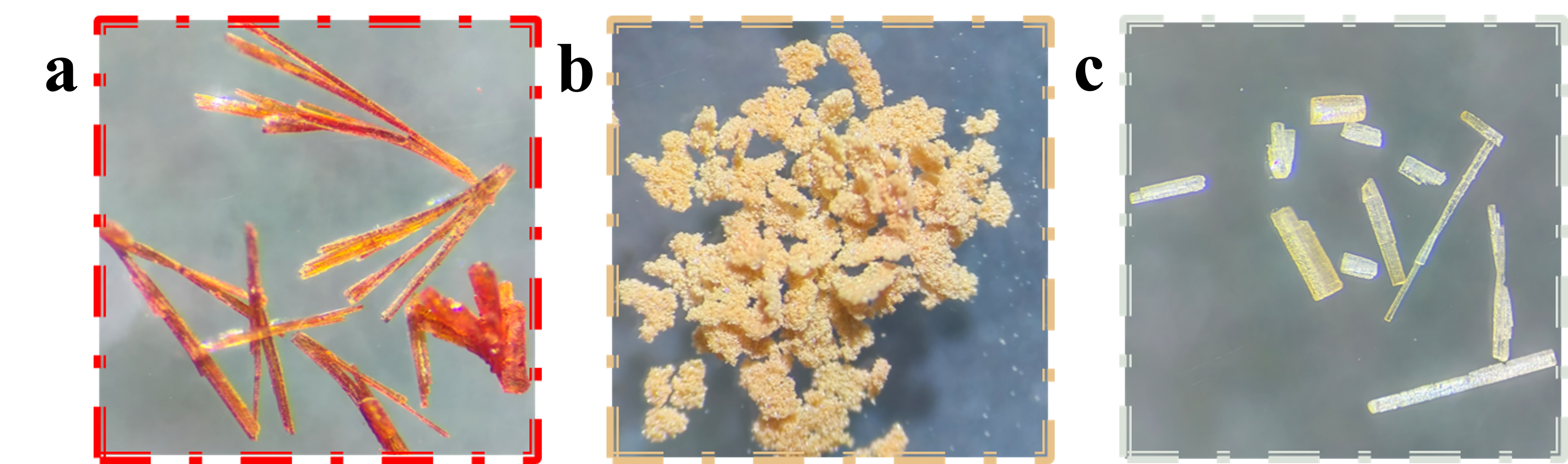


Figure S2. Macroscopic morphologies of (a) FeMOF, (b) CoMOF, and (c) ZnMOF, respectively.


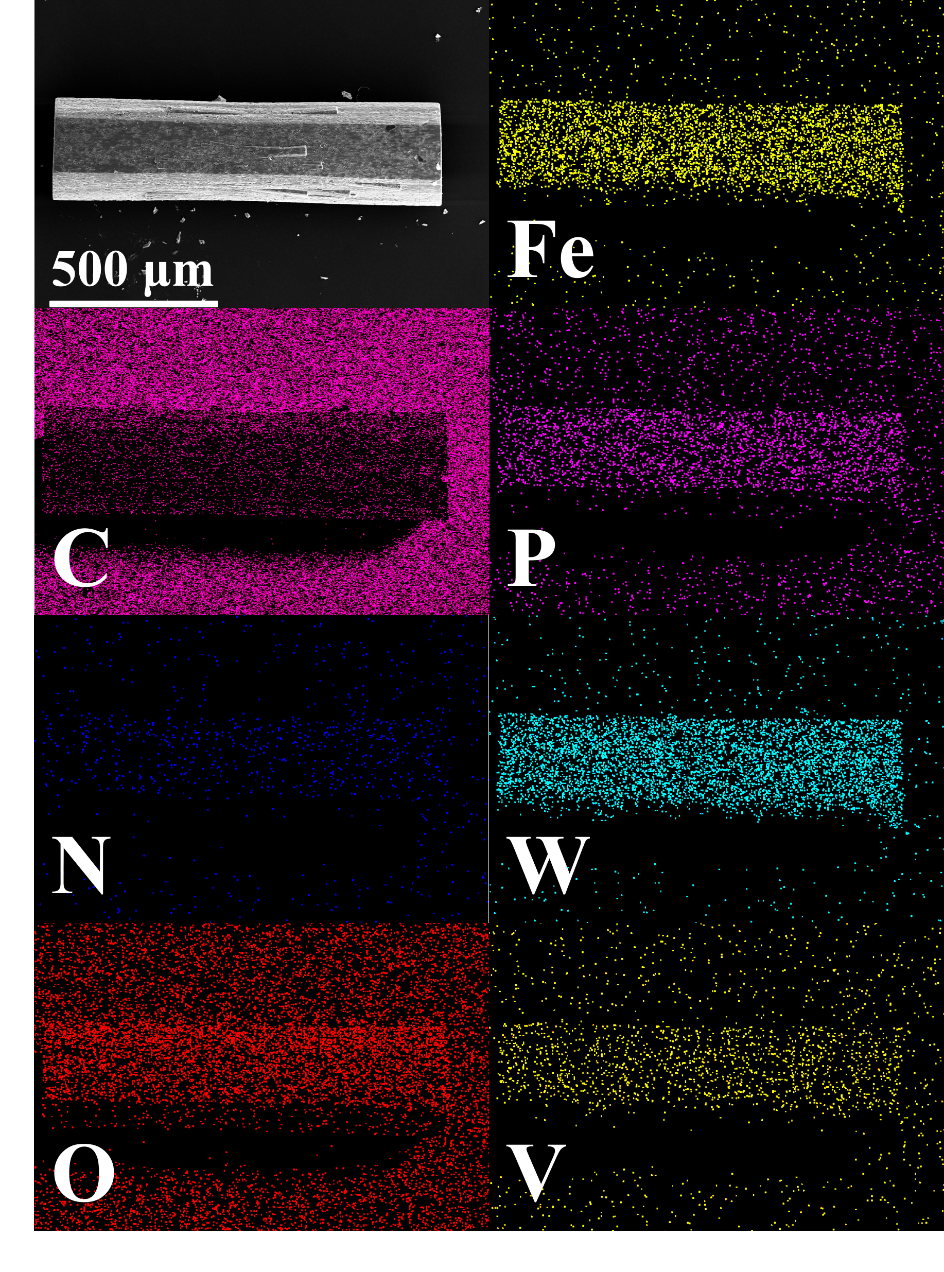


Figure S3. EDS elemental mapping images of Fe, C, N, O, P, W, and V in FeMOF-V_1_.


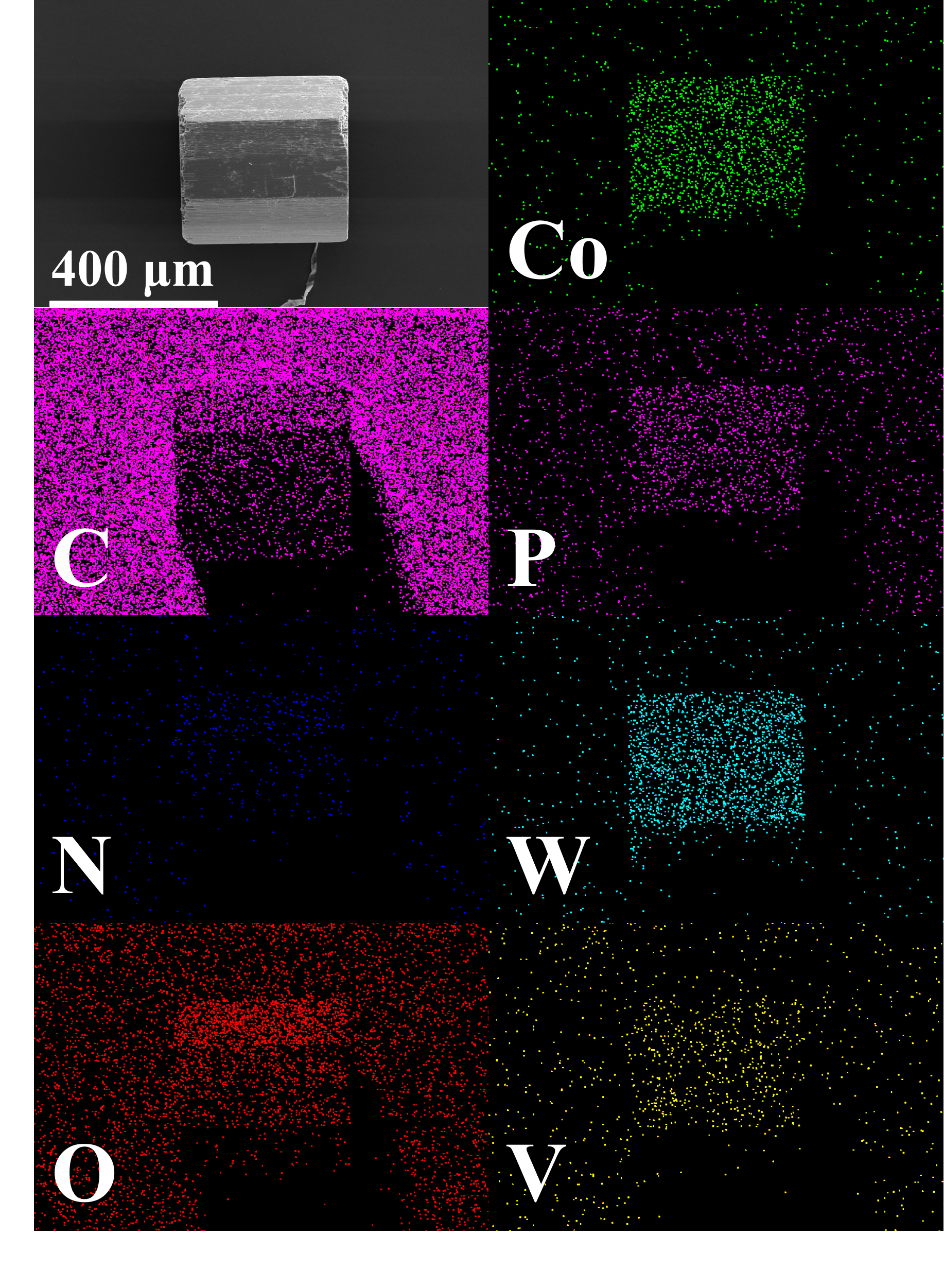


Figure S4. EDS elemental mapping images of Co, C, N, O, P, W, and V in CoMOF-V_1_.


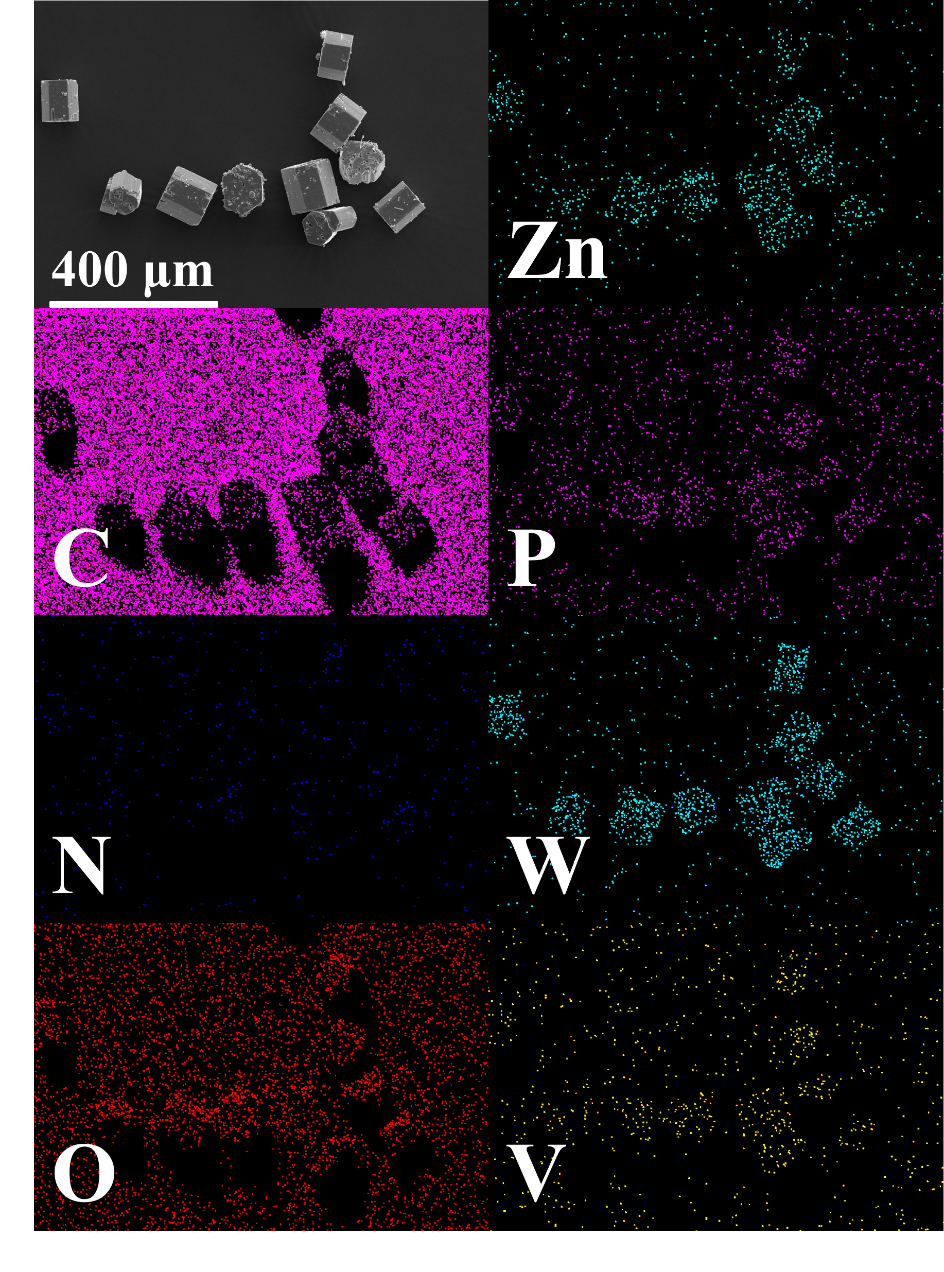


Figure S5. EDS elemental mapping images of Zn, C, N, O, P, W, and V in ZnMOF-V_1_.


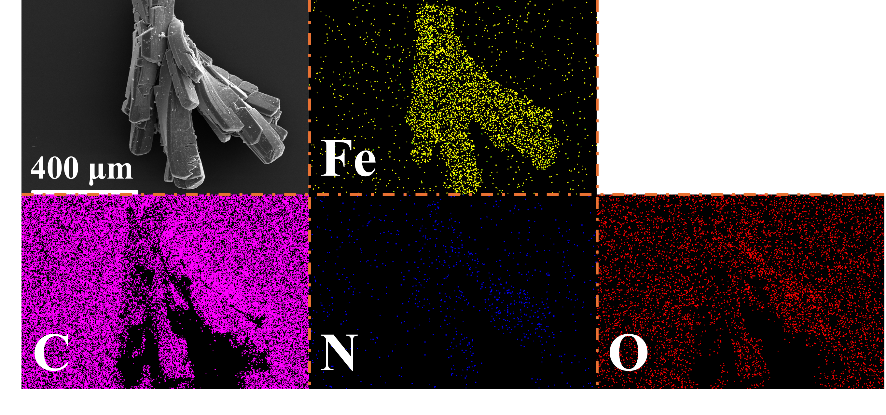


Figure S6. EDS elemental mapping images of Fe, C, N, and O in FeMOF.


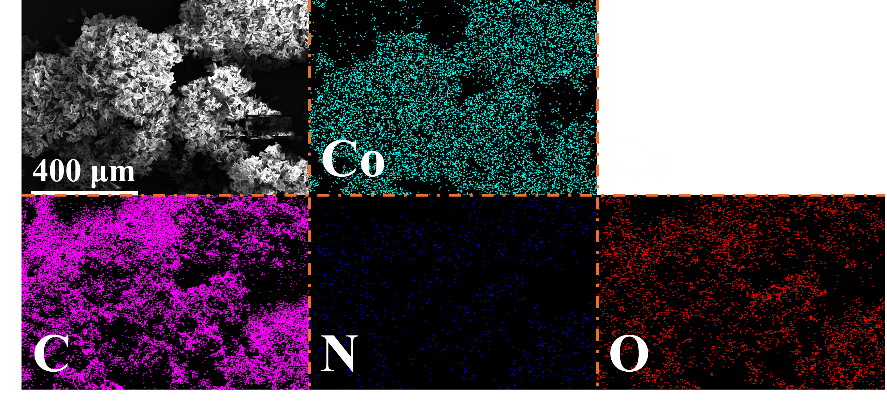


Figure S7. EDS elemental mapping images of Co, C, N, and O in CoMOF.


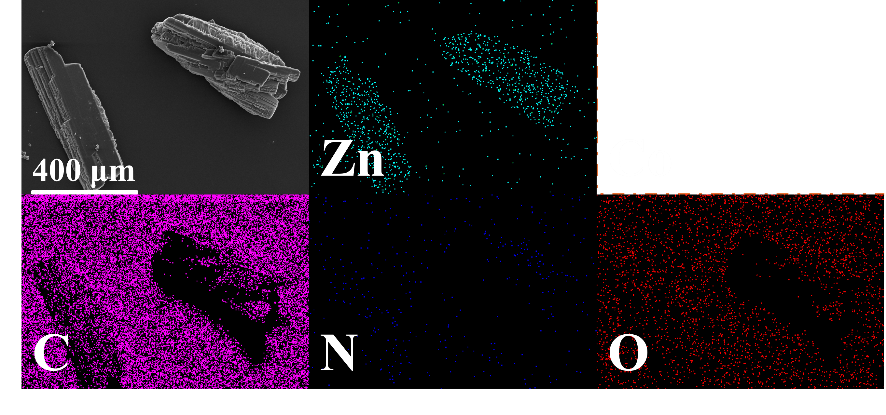


Figure S8. EDS elemental mapping images of Zn, C, N, and O in ZnMOF.


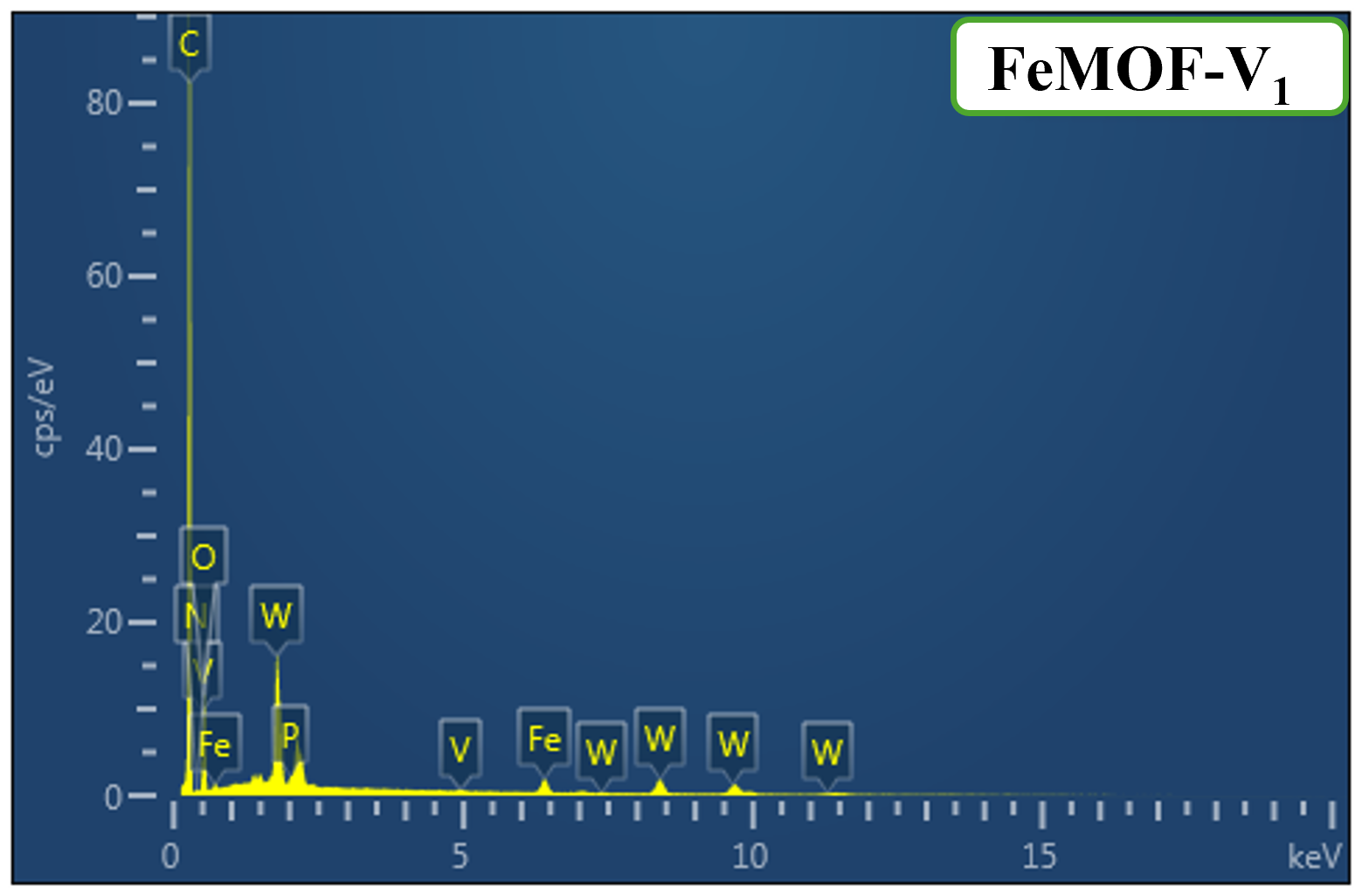


Figure S9. EDS spectrum for FeMOF-V_1_.


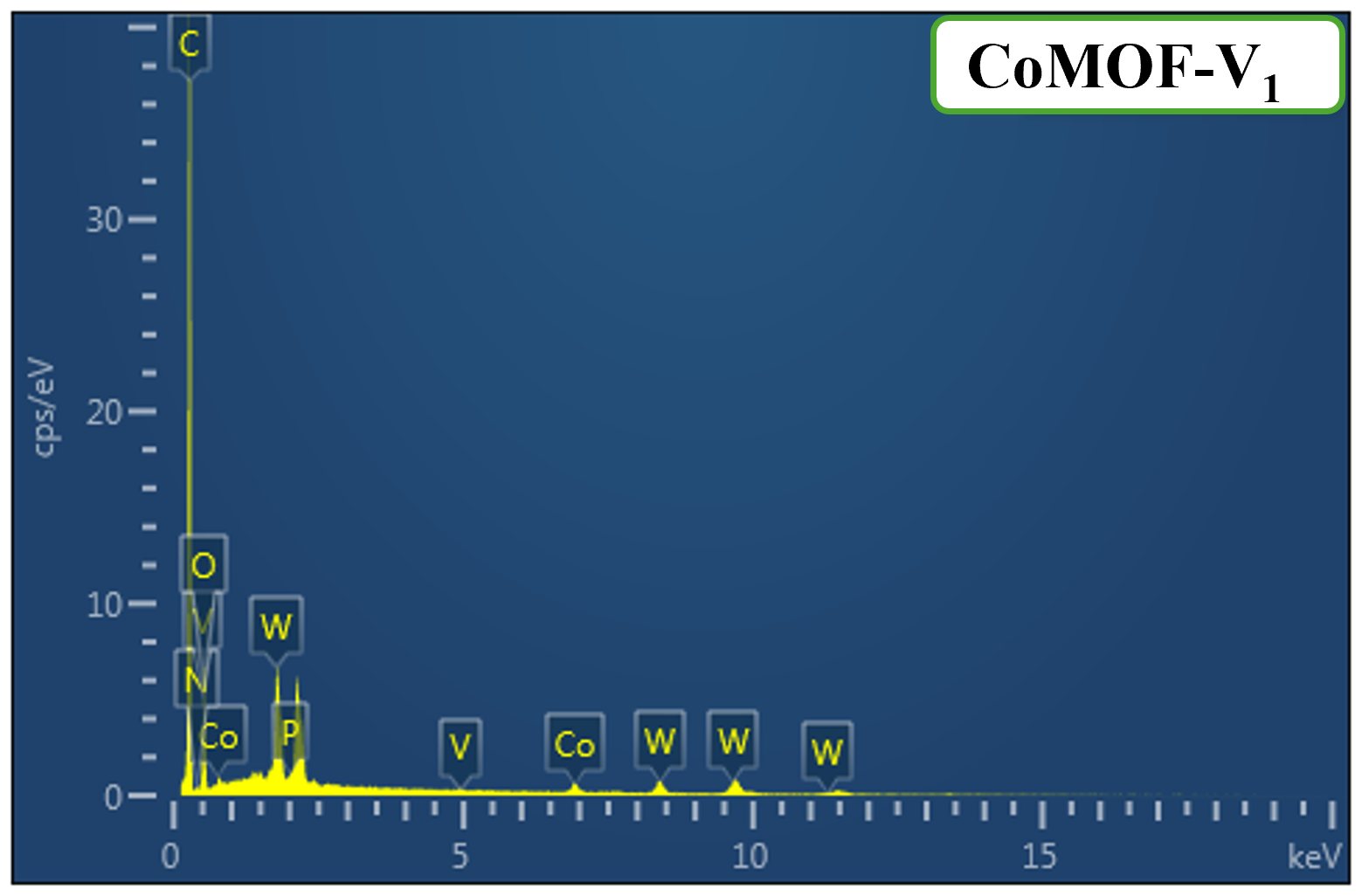


Figure S10. EDS spectrum for CoMOF-V_1_.


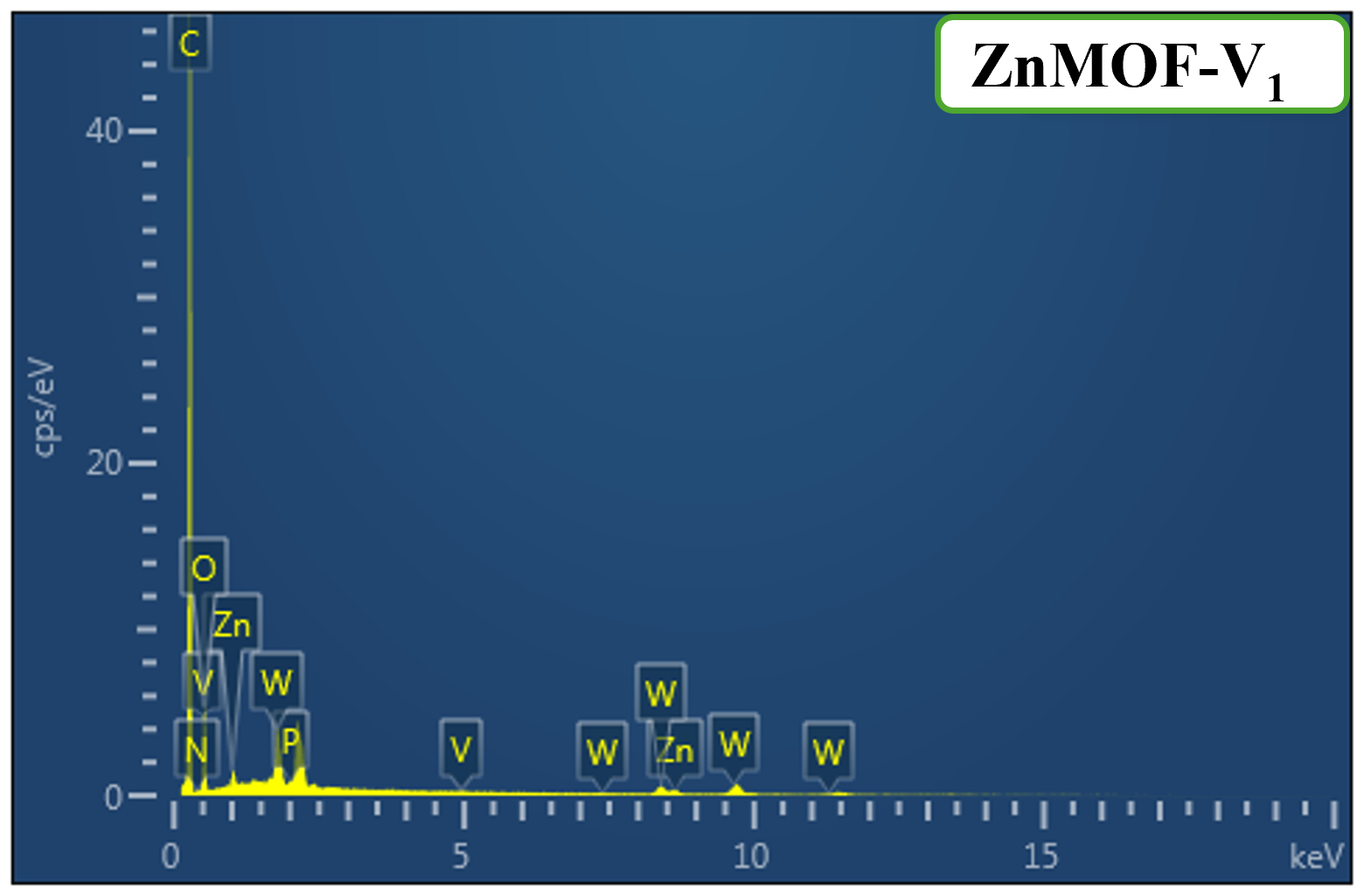


Figure S11. EDS spectrum for ZnMOF-V_1_.


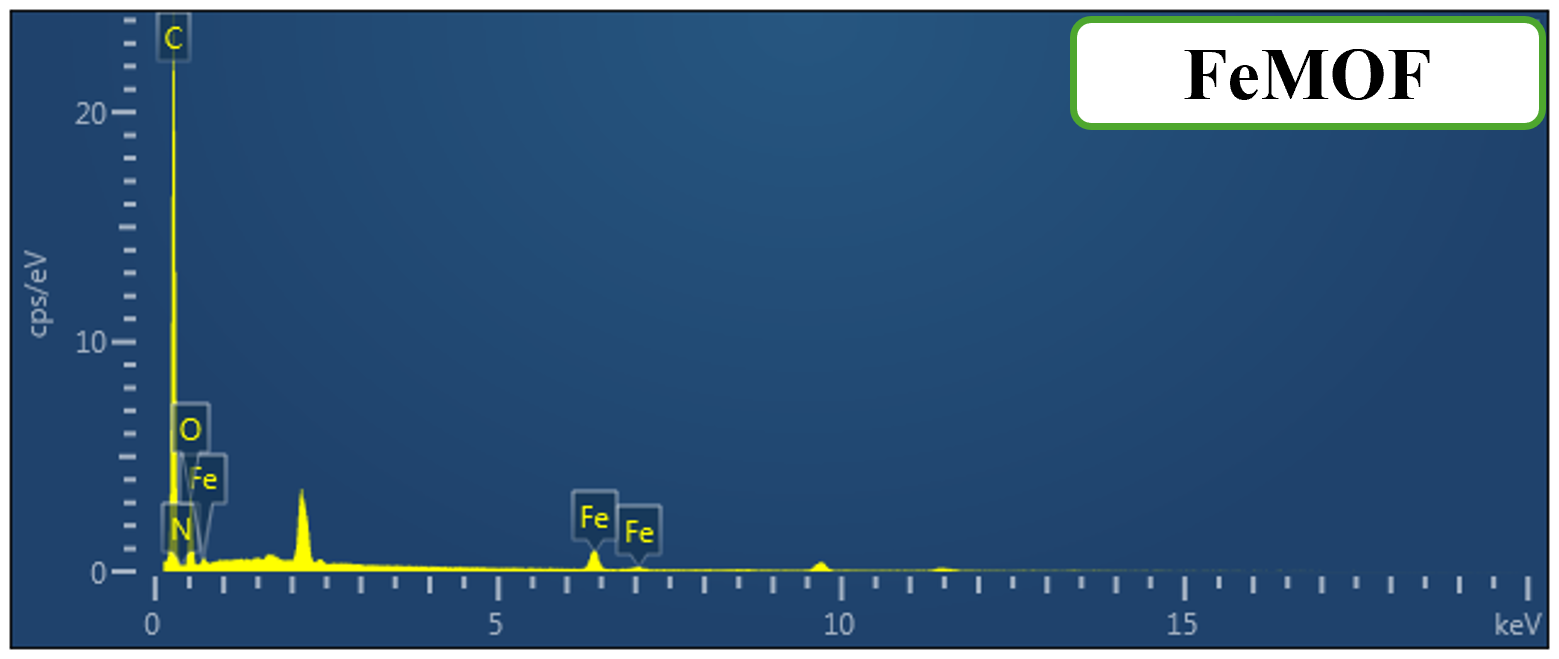


Figure S12. EDS spectrum for FeMOF.


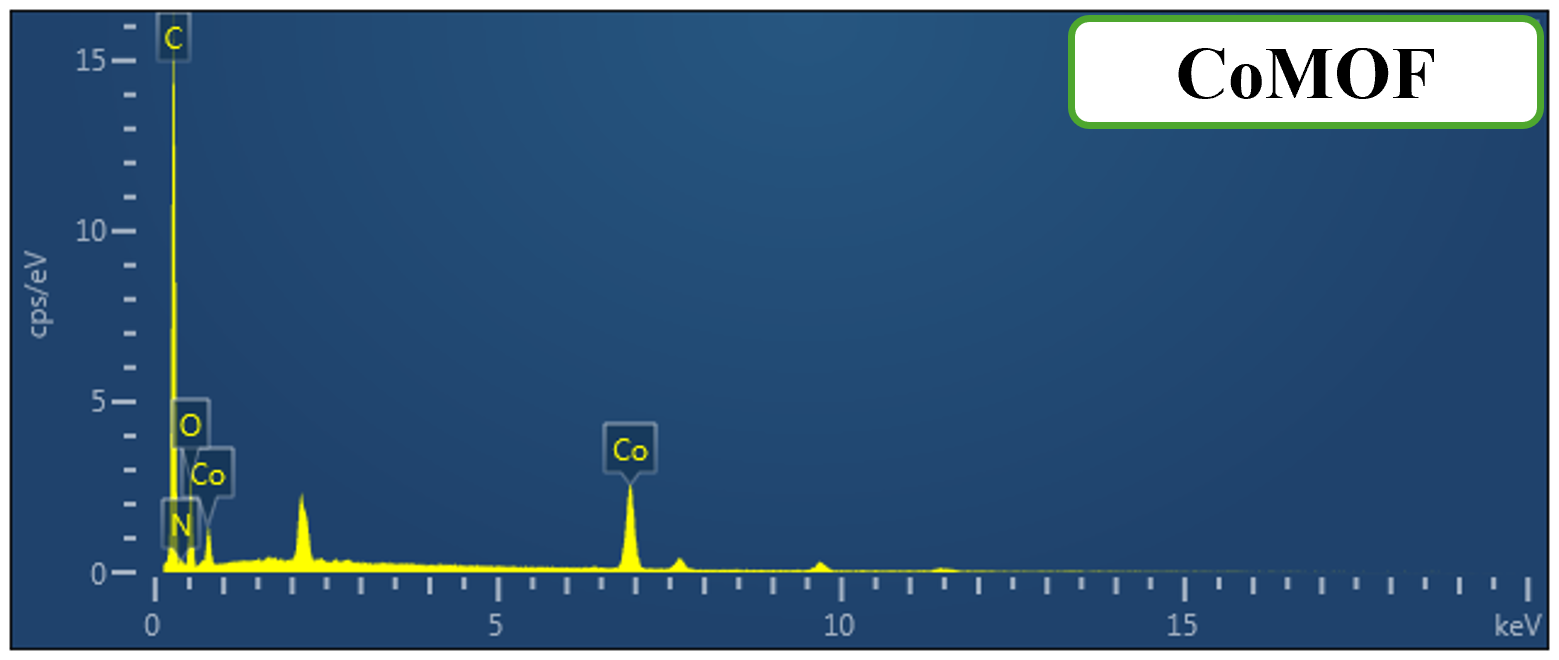


Figure S13. EDS spectrum for CoMOF.


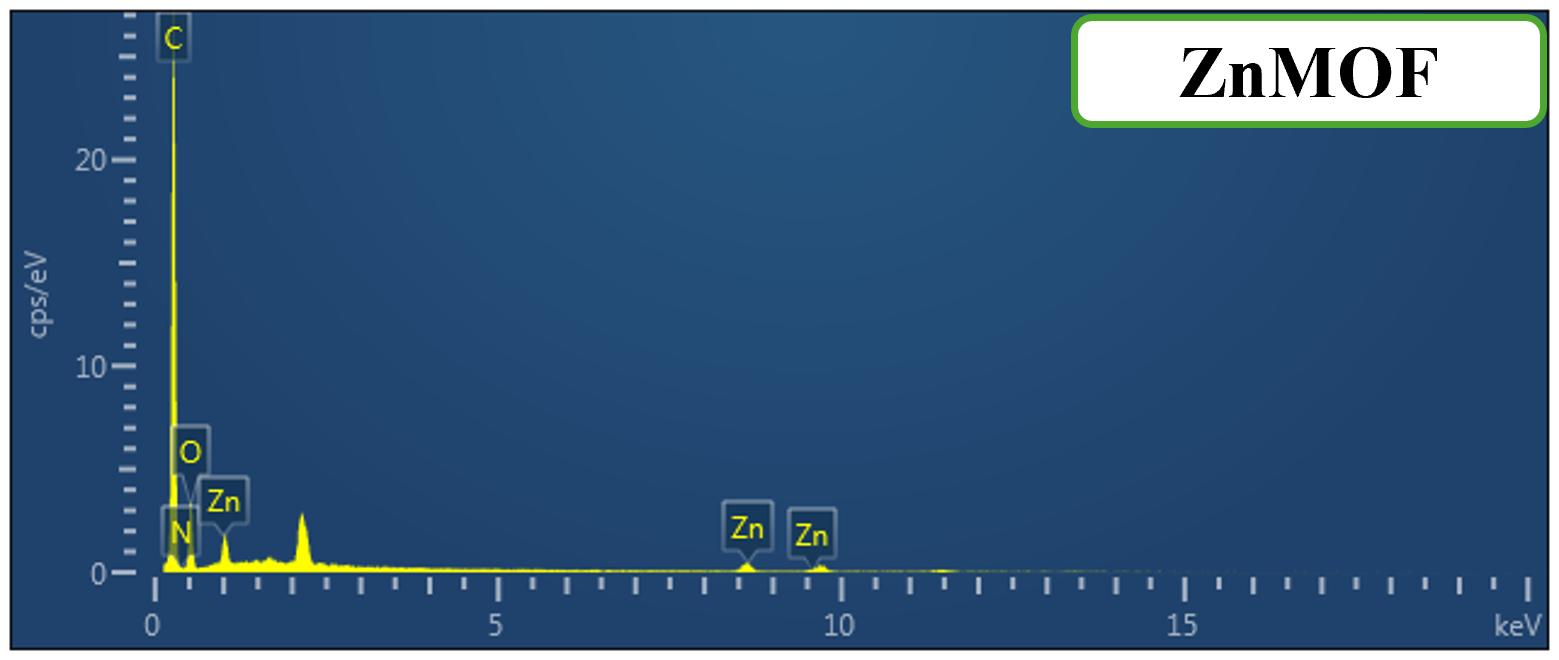


Figure S14. EDS spectrum for ZnMOF.


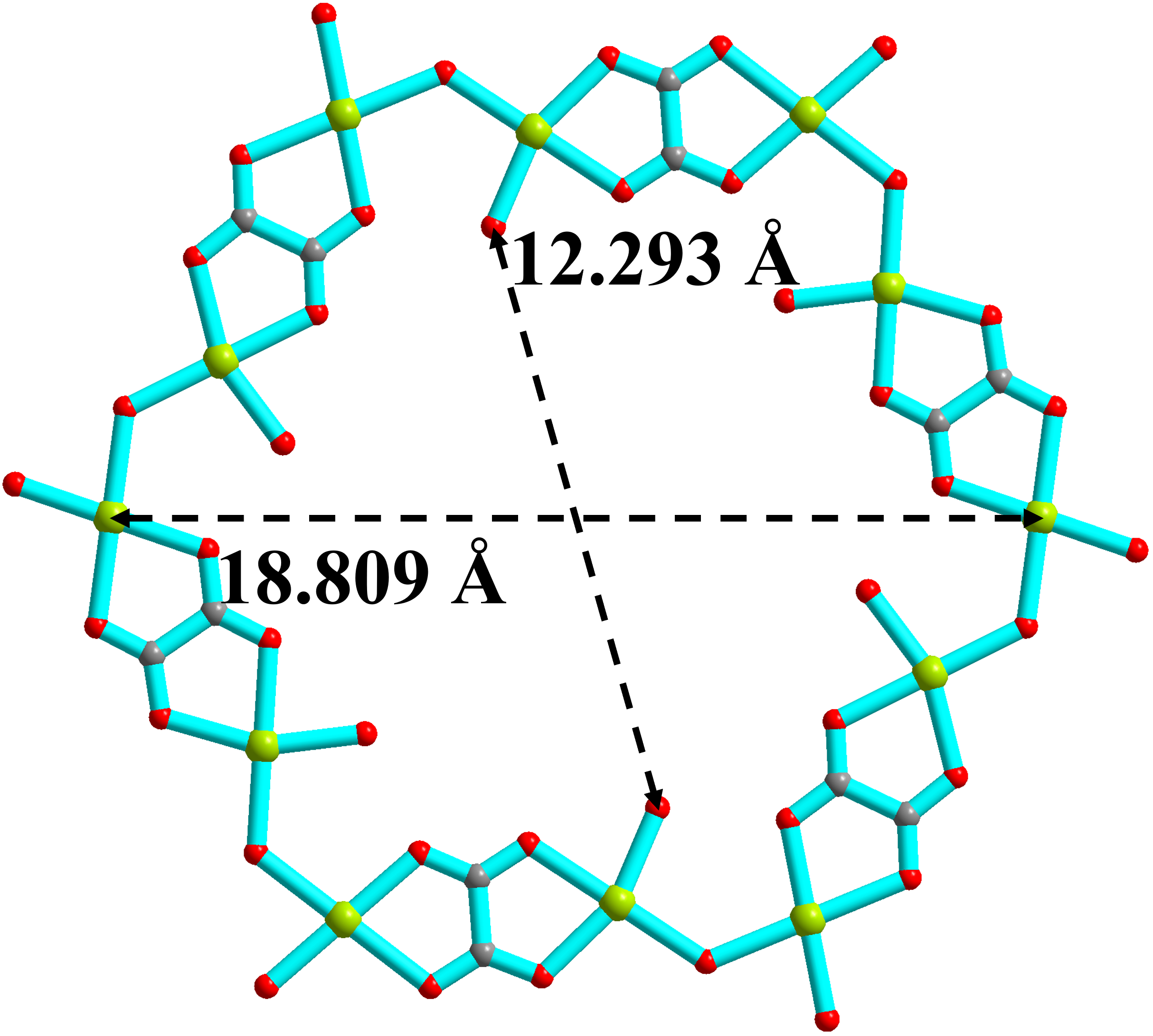


Figure S15. Fe/Co/Zn and DPYB constructed a twelve-membered ring with an 18.809 × 18.809 Å hole


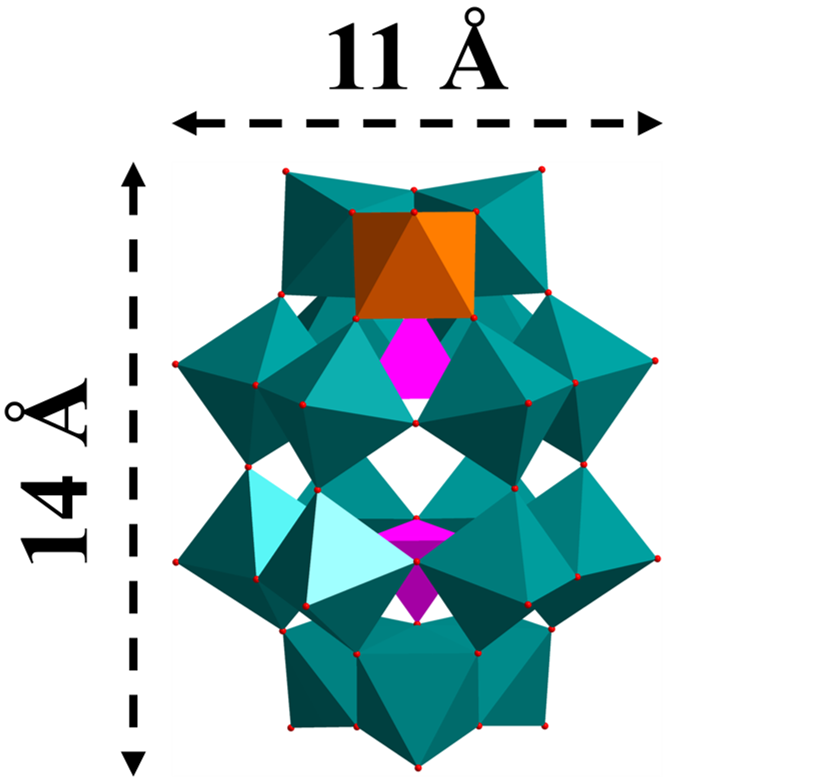


Figure S16. The dimension of P_2_W_17_V_1_ (14 × 11 Å)


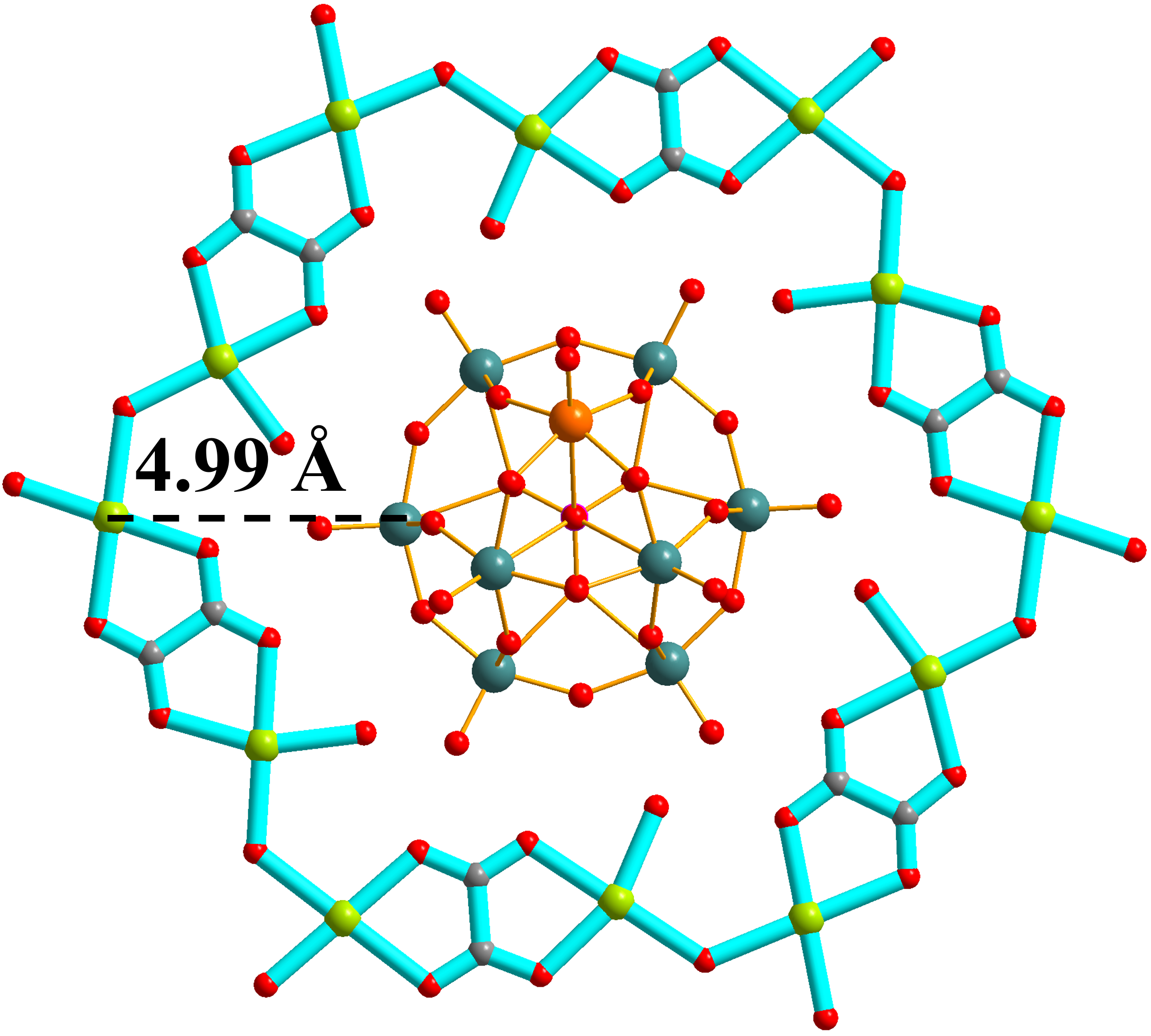


Figure S17. Distance from P_2_W_17_V_1_ to the Fe metal atoms


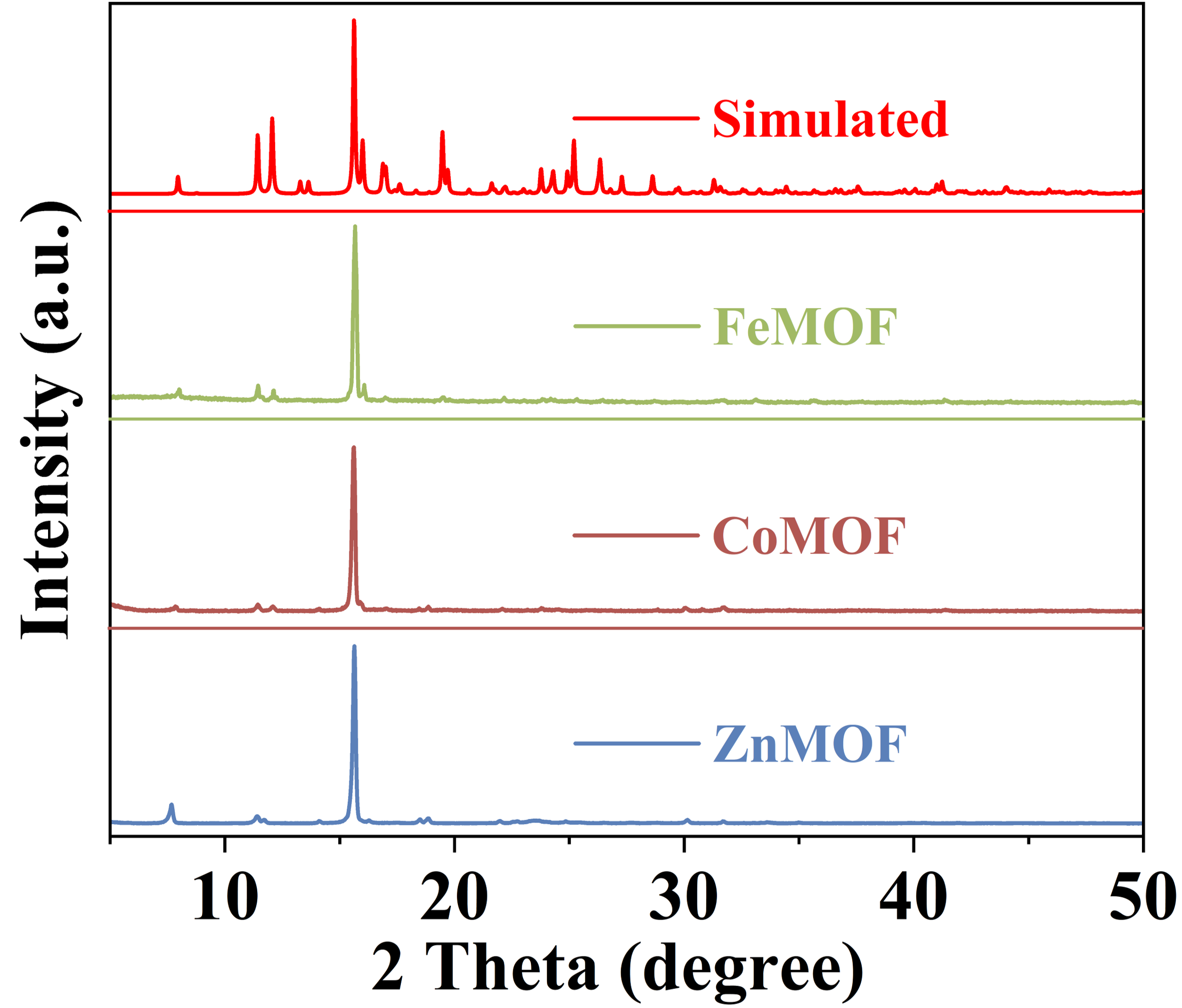


Figure S18. Simulated (top) and experimental measurement (bottom) PXRD of XMOF.


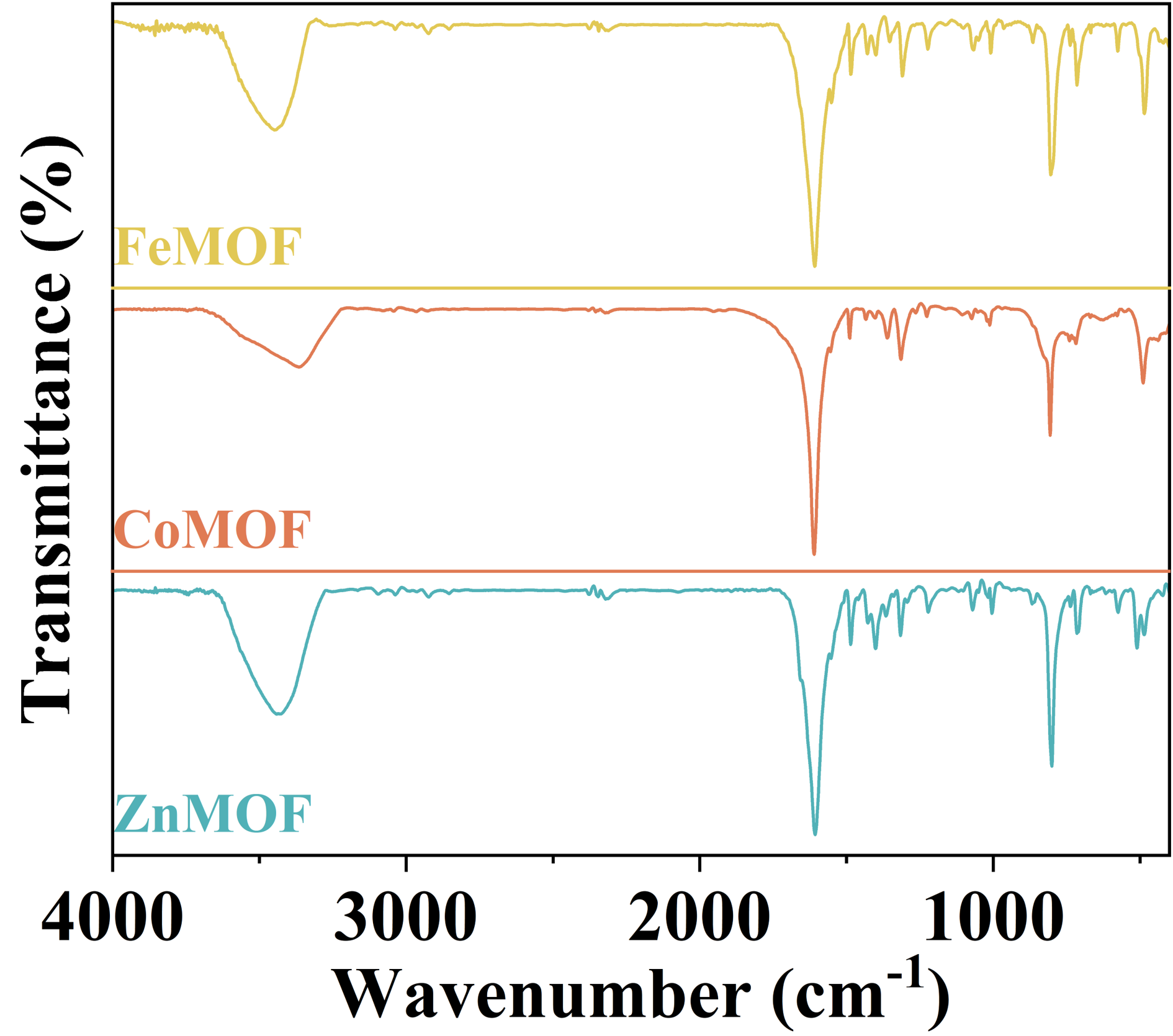


Figure S19. FTIR of XMOF.


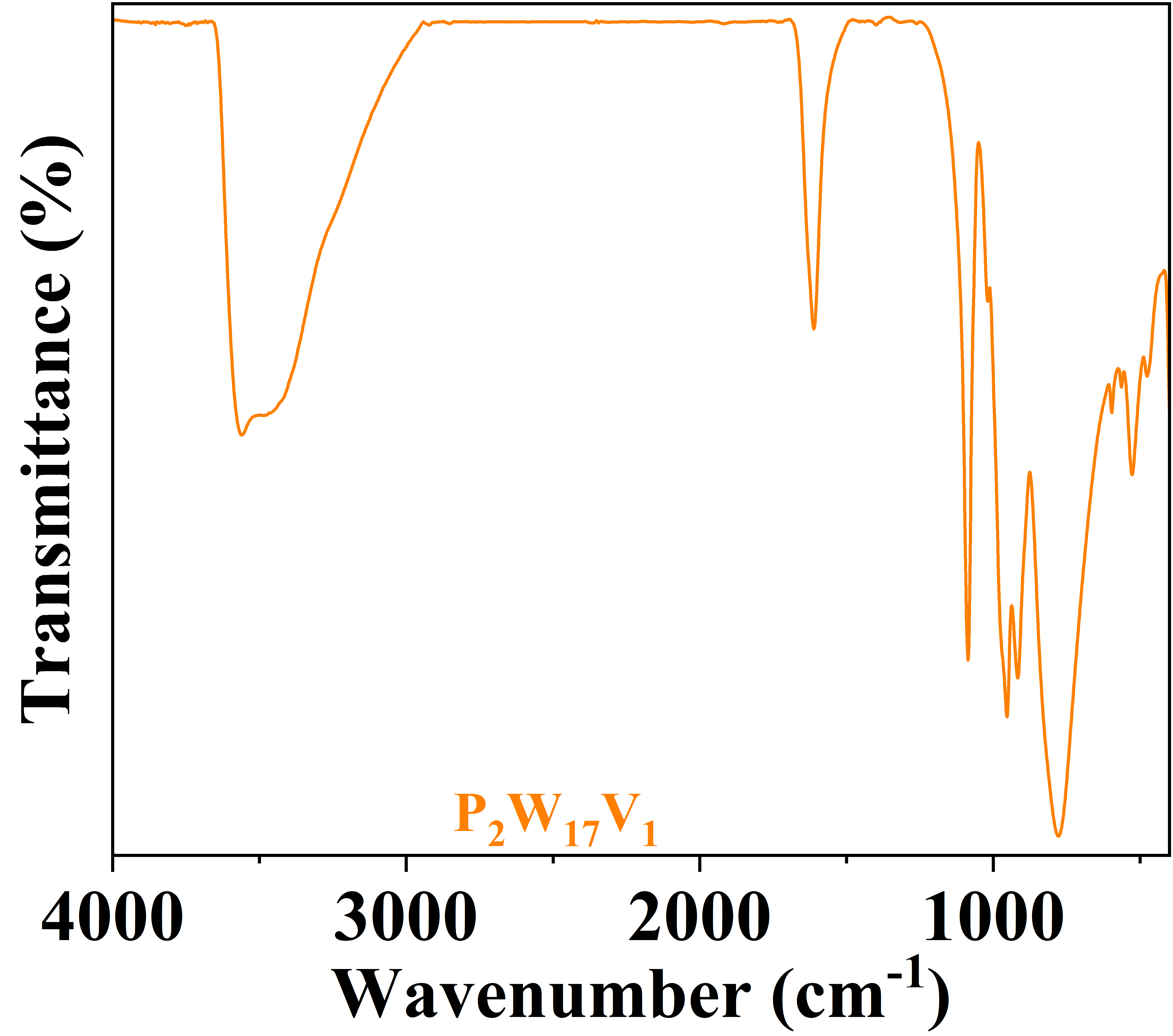


Figure S20. FTIR of P_2_W_17_V_1_.


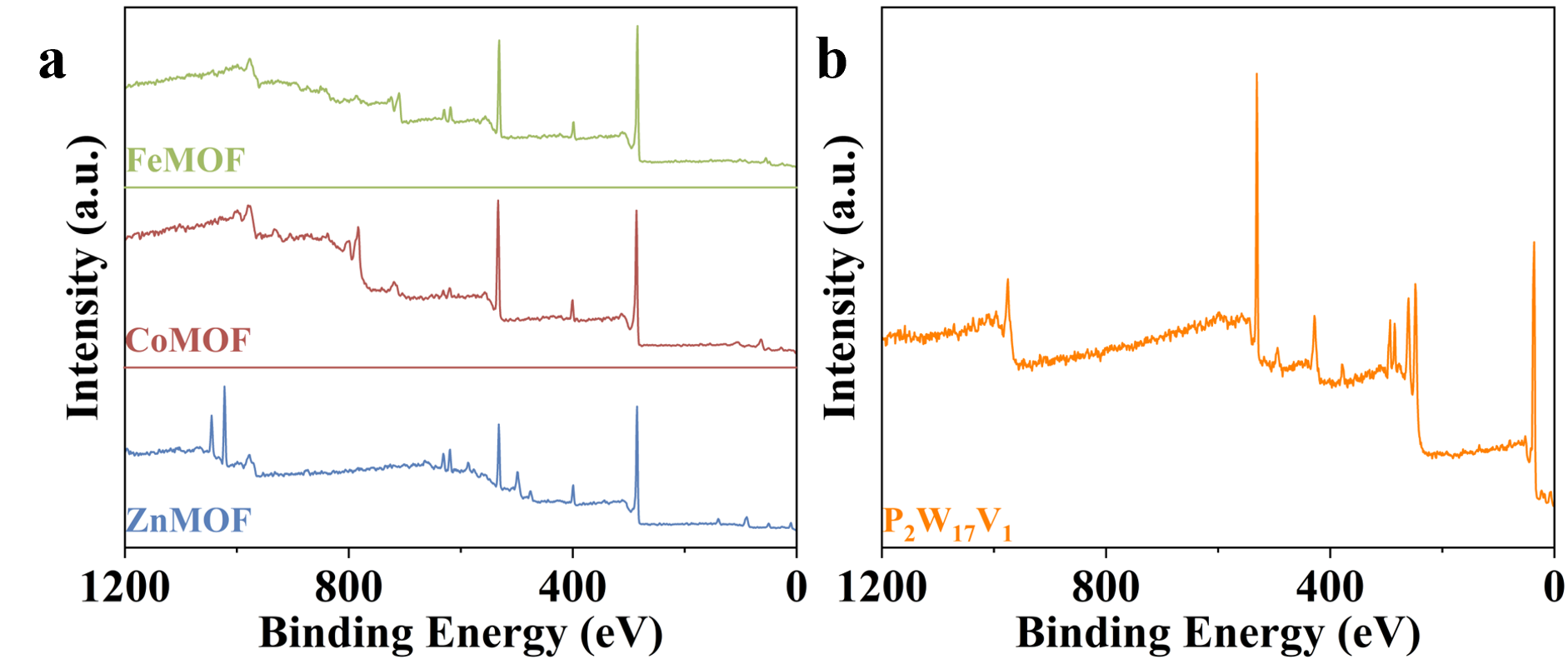


Figure S21. Survey XPS spectra of (a) XMOF and (b) P_2_W_17_V_1_


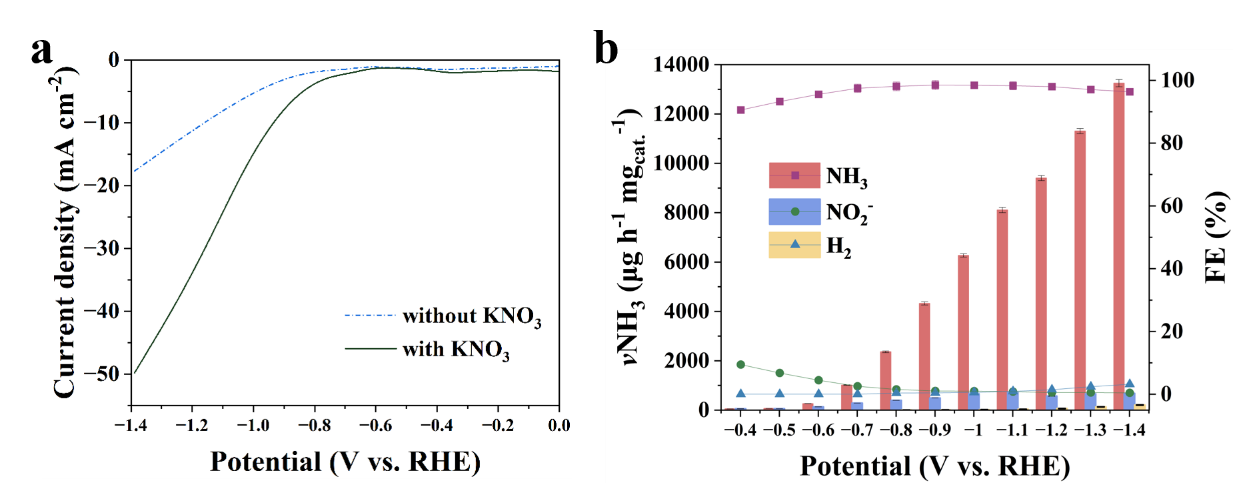


Figure S22. Electrocatalytic performances of FeMOF-V_1_: (a) LSV curves in 0.05 mol L^–1^ Na_2_SO_4_ with and without the addition of 0.1 mol L^–1^ KNO_3_. (b) Obtained NH_3_ yield rates and FEs by indophenol blue method.


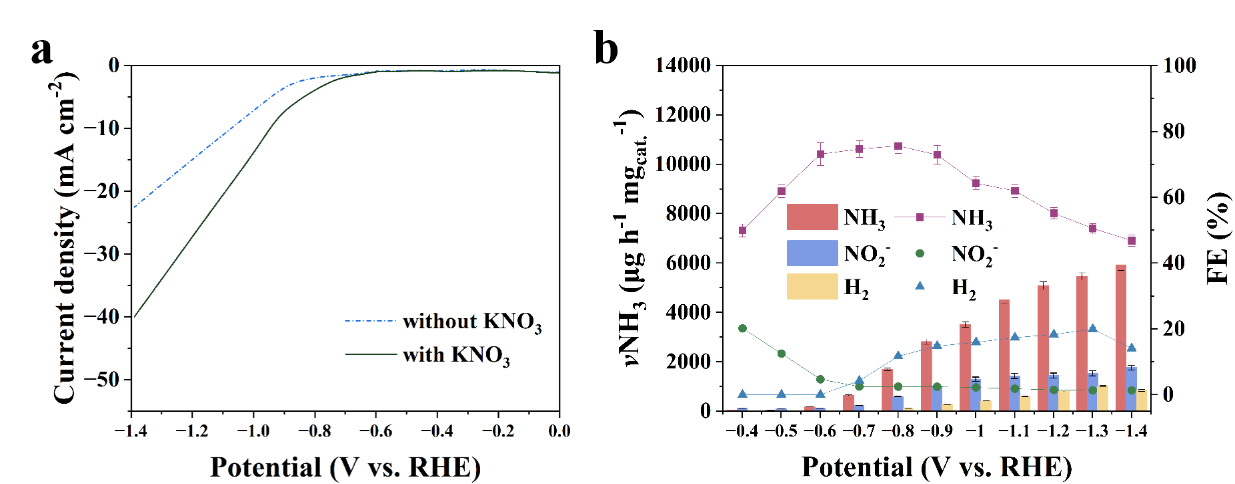
Figure S23. Electrocatalytic performances of CoMOF-V_1_: (a) LSV curves in 0.05 mol L^–1^ Na_2_SO_4_ with and without the addition of 0.1 mol L^–1^ KNO_3_. (b) Obtained NH_3_ yield rates and FEs by indophenol blue method.


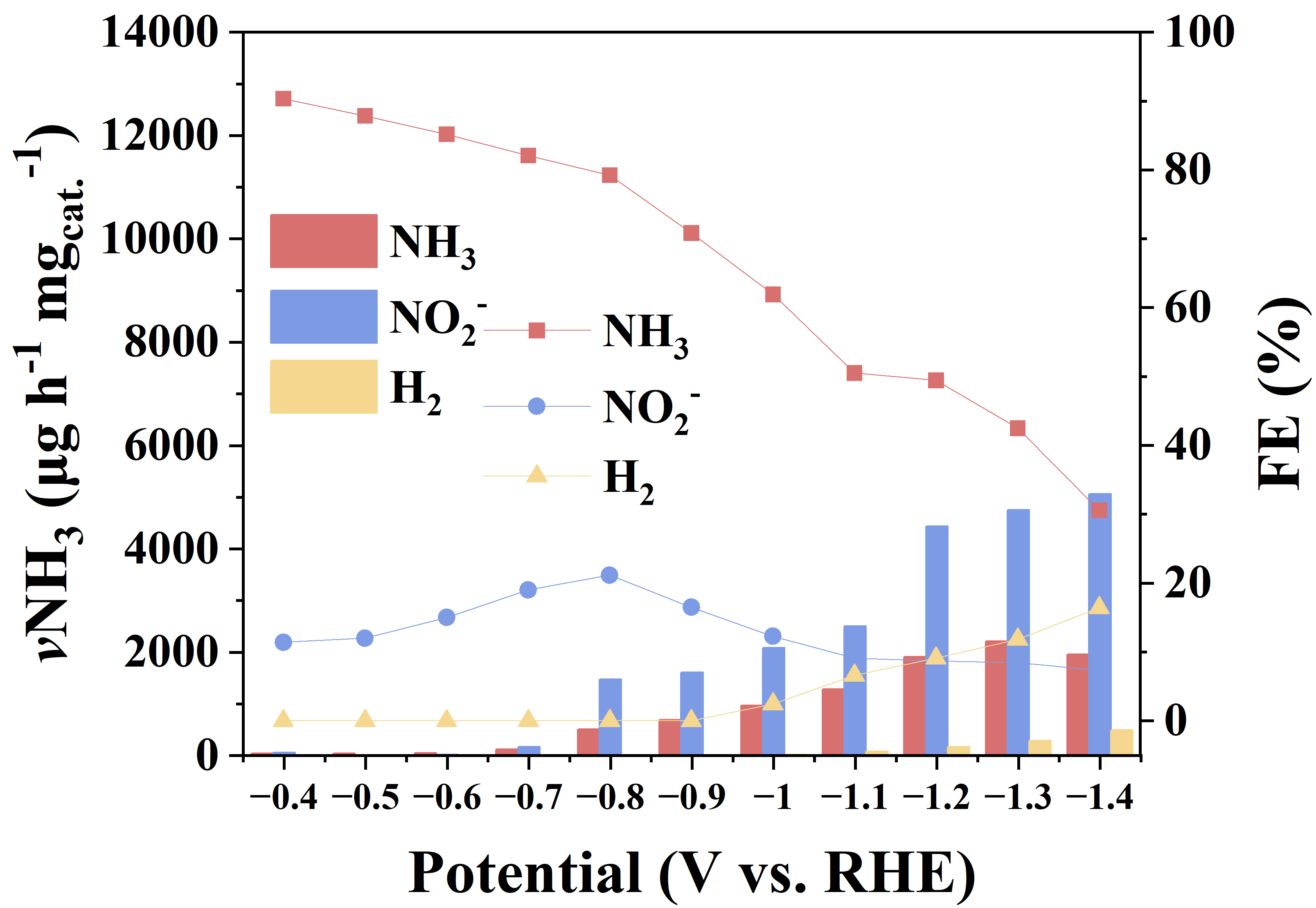

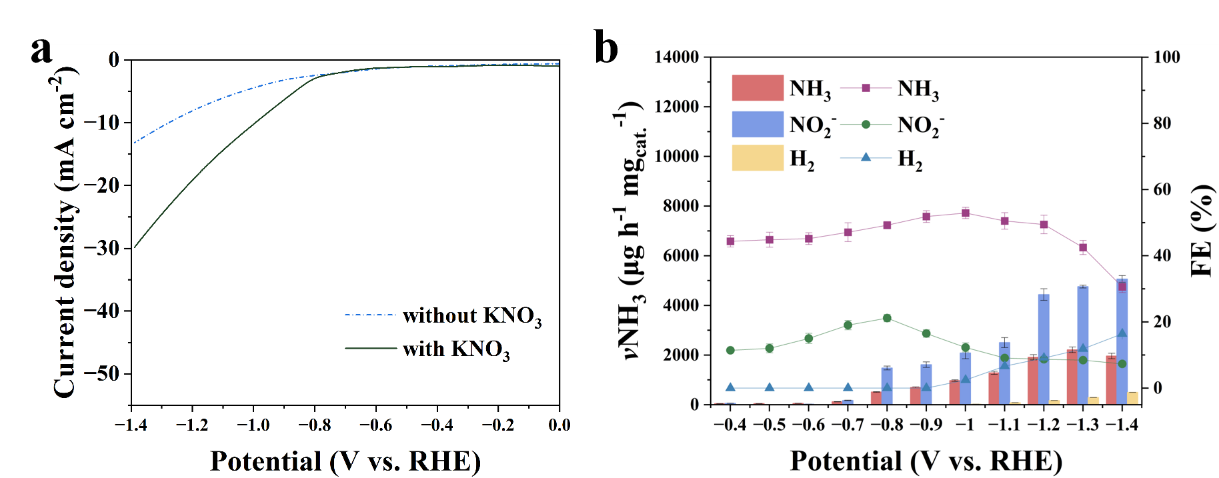


Figure S24. Electrocatalytic performances of ZnMOF-V_1_: (a) LSV curves in 0.05 mol L^–1^ Na_2_SO_4_ with and without the addition of 0.1 mol L^–1^ KNO_3_. (b) Obtained NH_3_ yield rates and FEs by indophenol blue method.


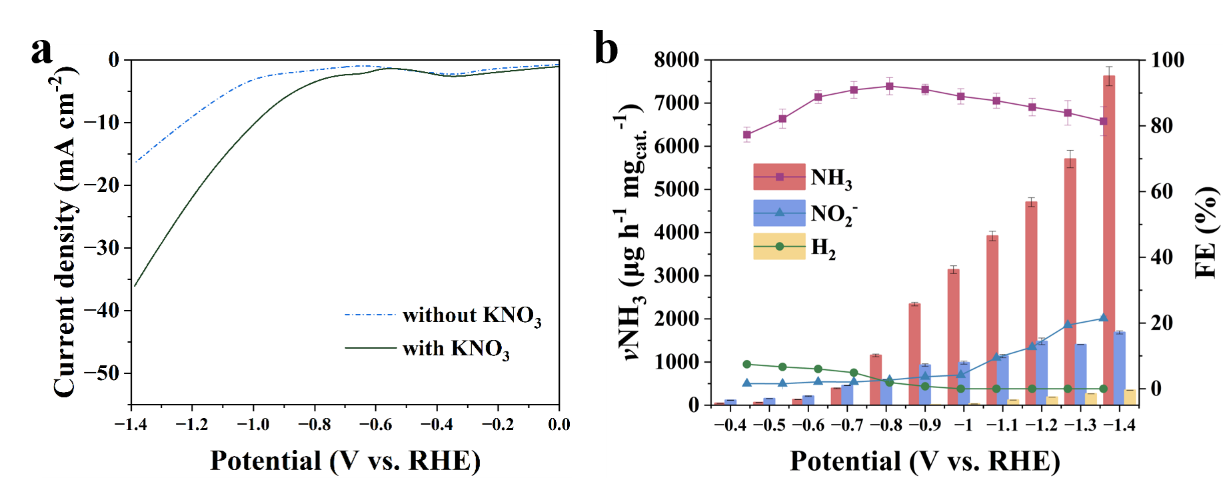


Figure S25. Electrocatalytic performances of FeMOF: (a) LSV curves in 0.05 mol L^–1^ Na_2_SO_4_ with and without the addition of 0.1 mol L^–1^ KNO_3_. (b) Obtained NH_3_ yield rates and FEs by indophenol blue method.


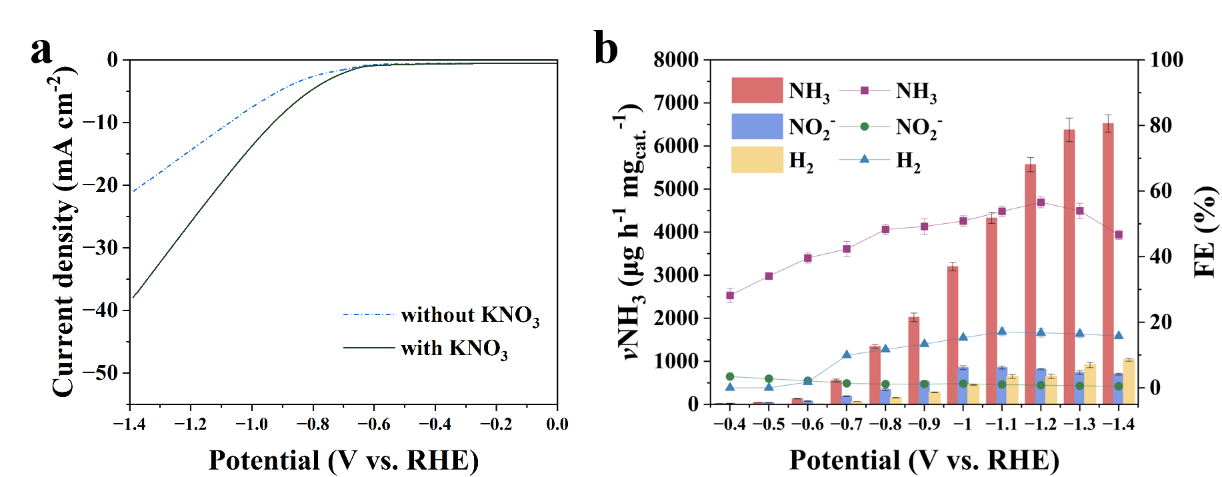


Figure S26. Electrocatalytic performances of CoMOF: (a) LSV curves in 0.05 mol L^–1^ Na_2_SO_4_ with and without the addition of 0.1 mol L^–1^ KNO_3_. (b) Obtained NH_3_ yield rates and FEs by indophenol blue method.


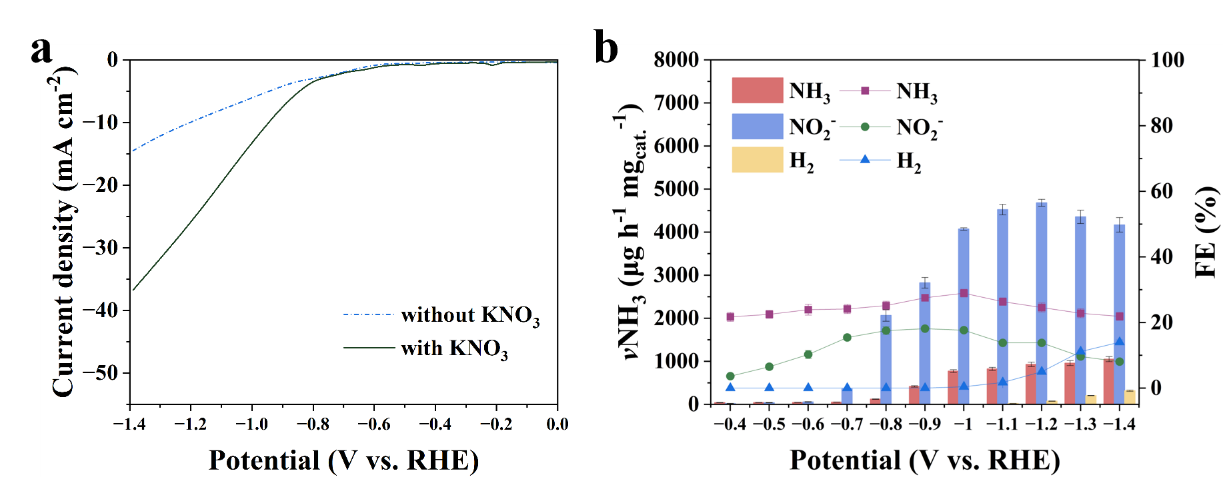


Figure S27. Electrocatalytic performances of ZnMOF: (a) LSV curves in 0.05 mol L^–1^ Na_2_SO_4_ with and without the addition of 0.1 mol L^–1^ KNO_3_. (b) Obtained NH_3_ yield rates and FEs by indophenol blue method.


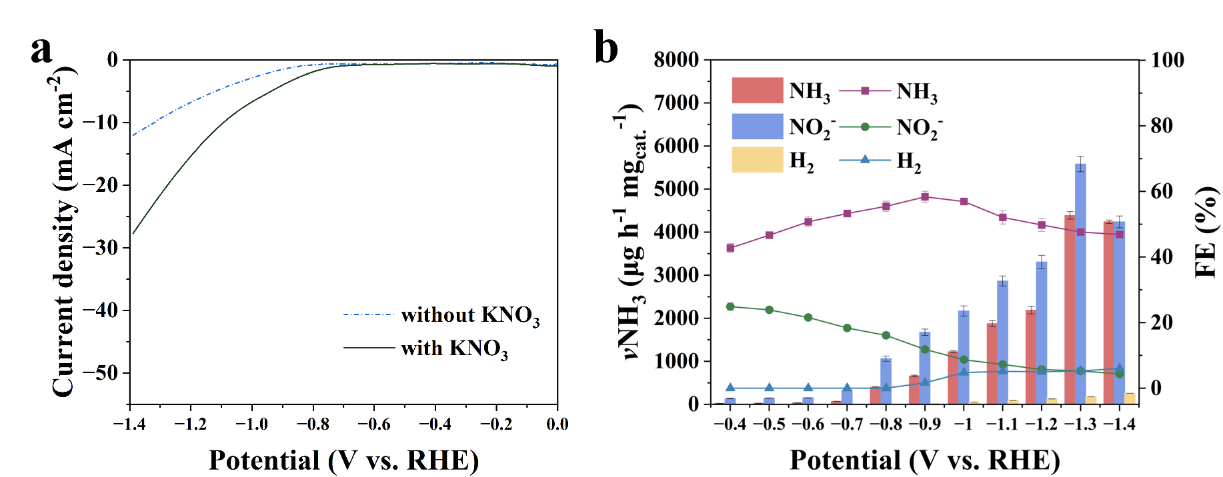


Figure S28. Electrocatalytic performances of P_2_W_17_V_1_: (a) LSV curves in 0.05 mol L^–1^ Na_2_SO_4_ with and without the addition of 0.1 mol L^–1^ KNO_3_. (b) Obtained NH_3_ yield rates and FEs by indophenol blue method.


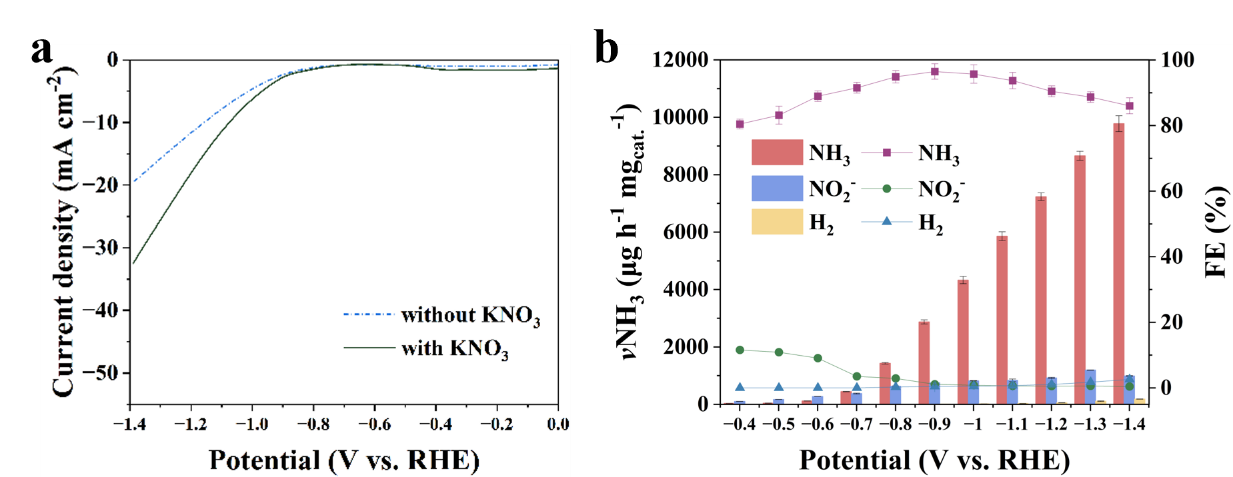


Figure S29. Electrocatalytic performances of FeMOF+P_2_W_17_V_1_: (a) LSV curves in 0.05 mol L^–1^ Na_2_SO_4_ with the addition of 0.1 mol L^–1^ KNO_3_ and without KNO_3_. (b) Obtained NH_3_ yield rates and FEs by indophenol blue method.


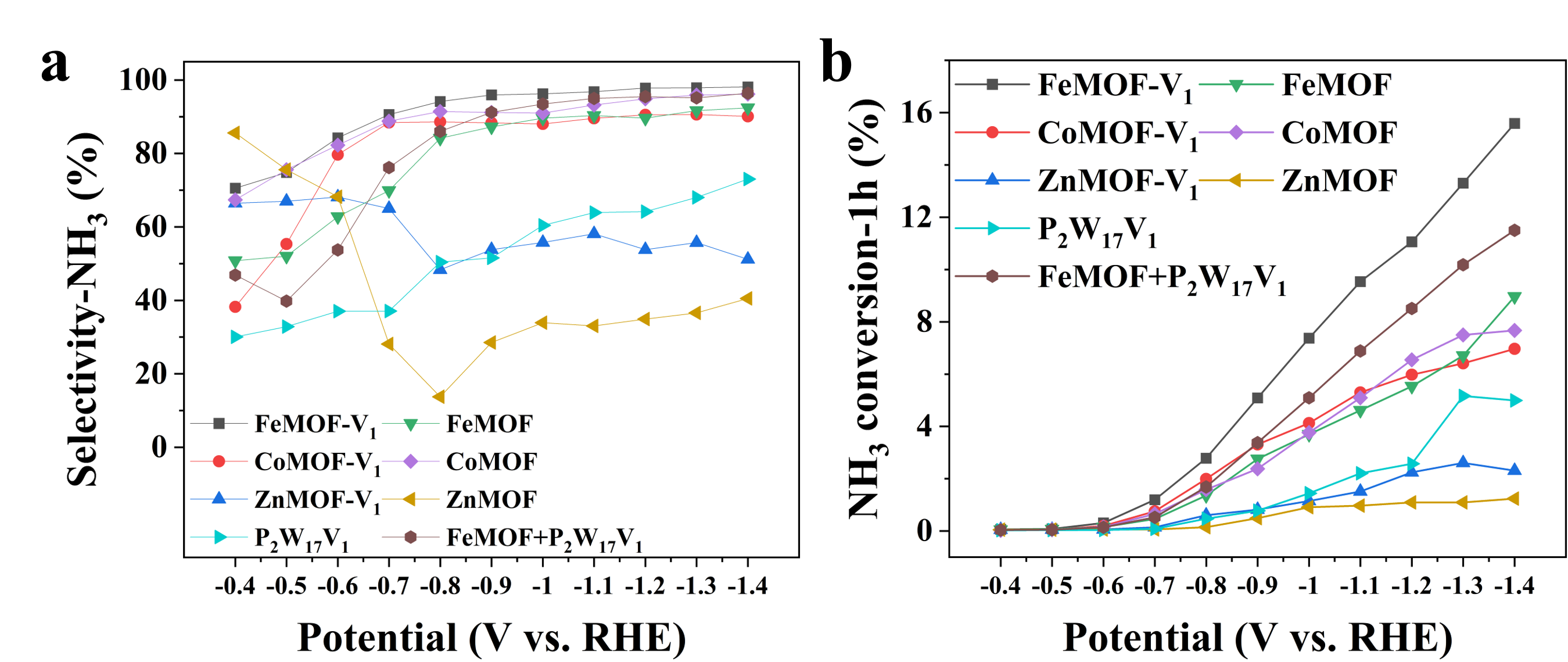


Figure S30. (a) The Selectivity-NH_3_ of XMOF-V_1_, (b) the corresponding NH_3_ conversion of XMOF-V_1_.


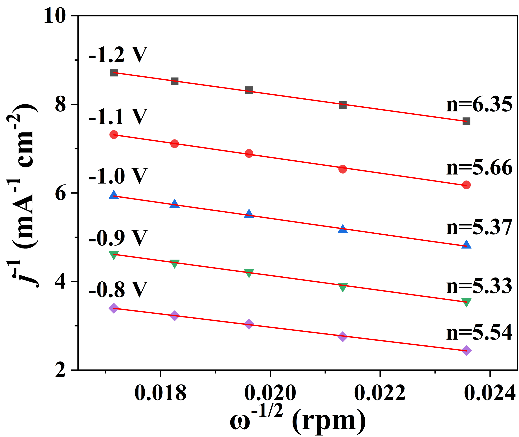


Figure S31. K–L plot at various potentials of the RDE study of FeMOF-V_1_.


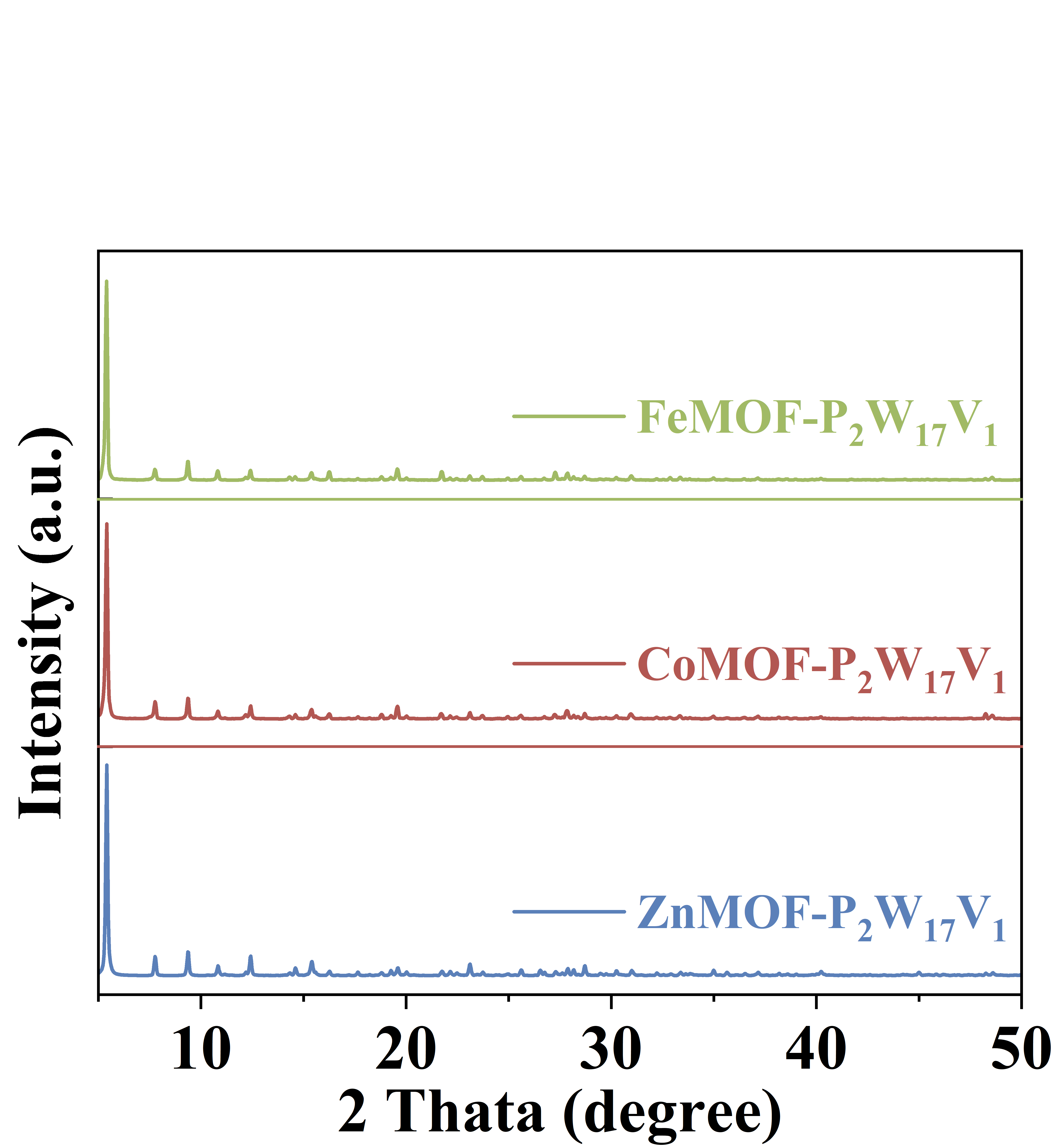


Figure S32. PXRD of XMOF-V_1_ after immersion in 0.05 M Na_2_SO_4_ (pH = 7) containing 0.1 M KNO_3_ for over 48 h.


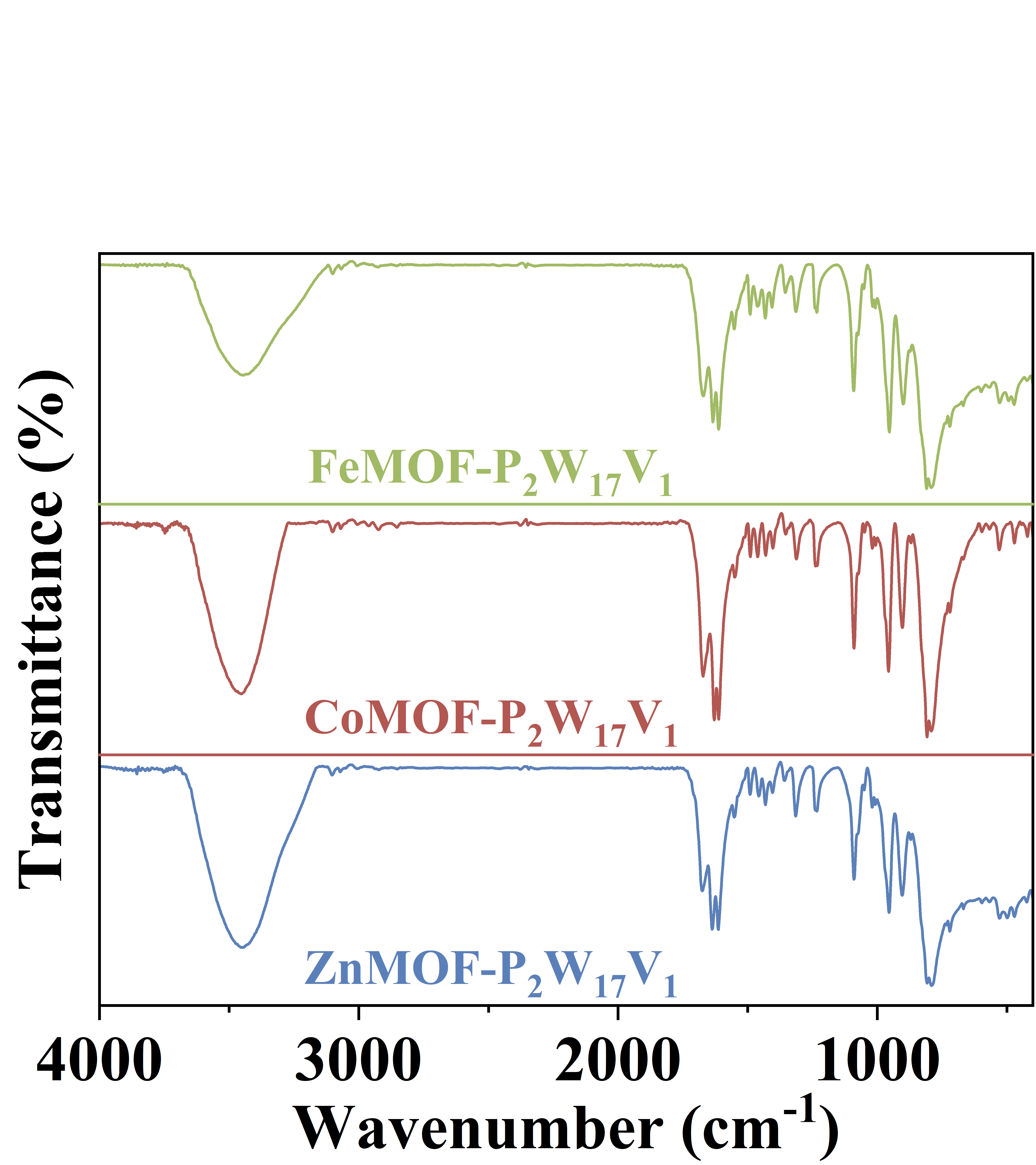


Figure S33. FTIR of XMOF-P_2_W_17_V_1_ after immersion in 0.05 M Na_2_SO_4_ (pH = 7) containing 0.1 M KNO_3_ for over 48 h.


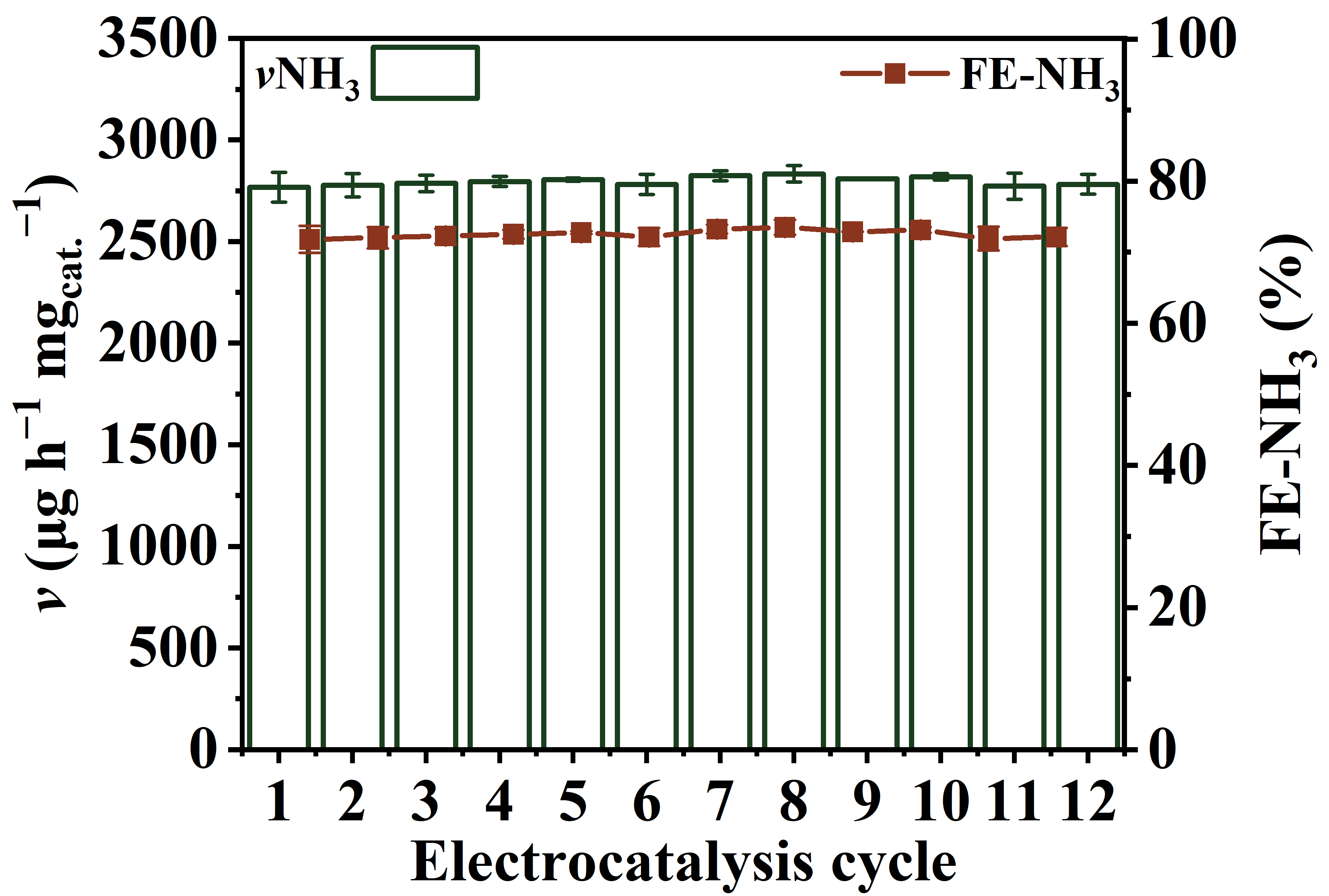


Figure S34. Chronoamperometry tests at a constant potential of −0.9 V vs. RHE for 12 cycles of CoMOF-V1.


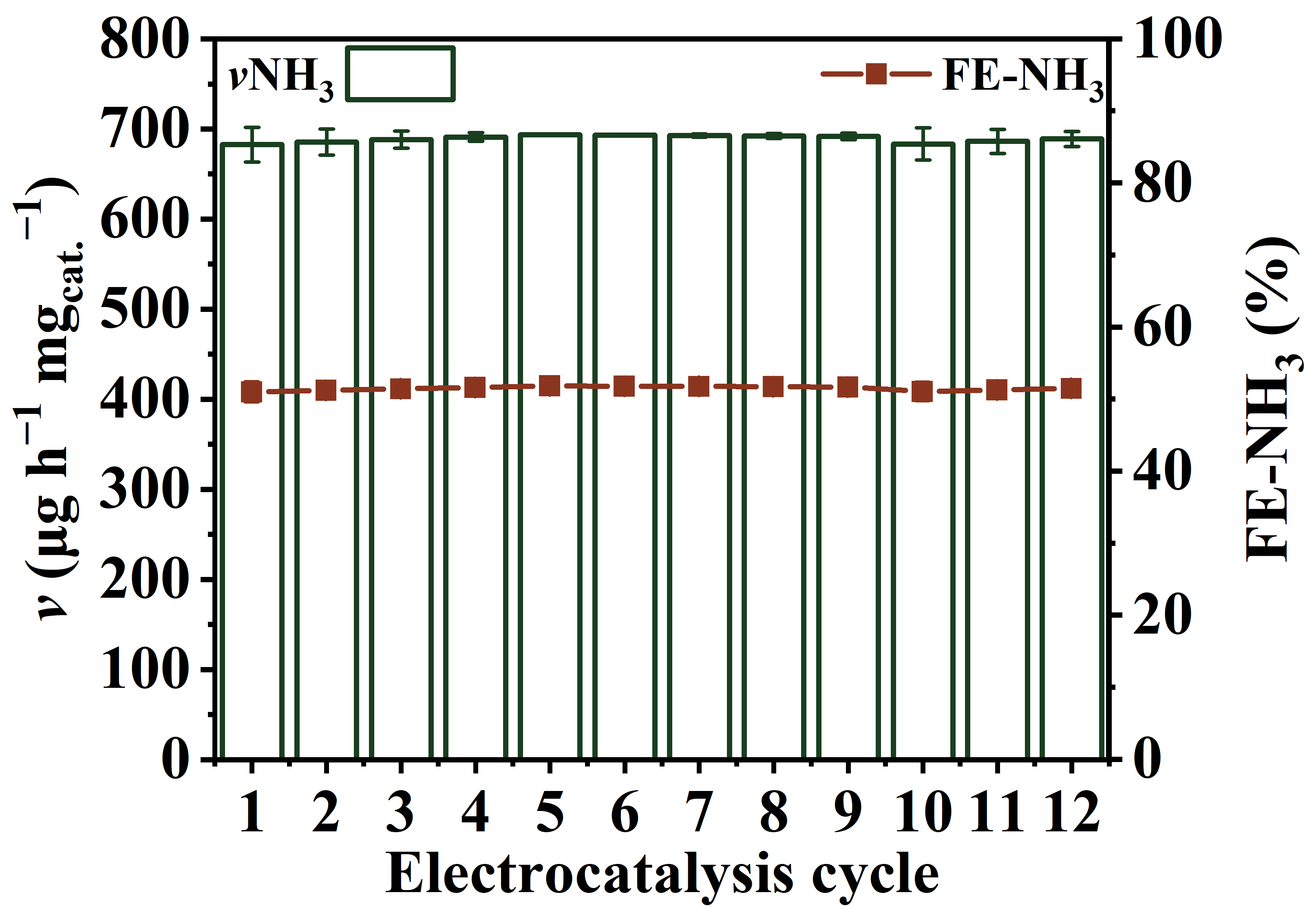


Figure S35. Chronoamperometry tests at a constant potential of −0.9 V vs. RHE for 12 cycles of ZnMOF-V1.


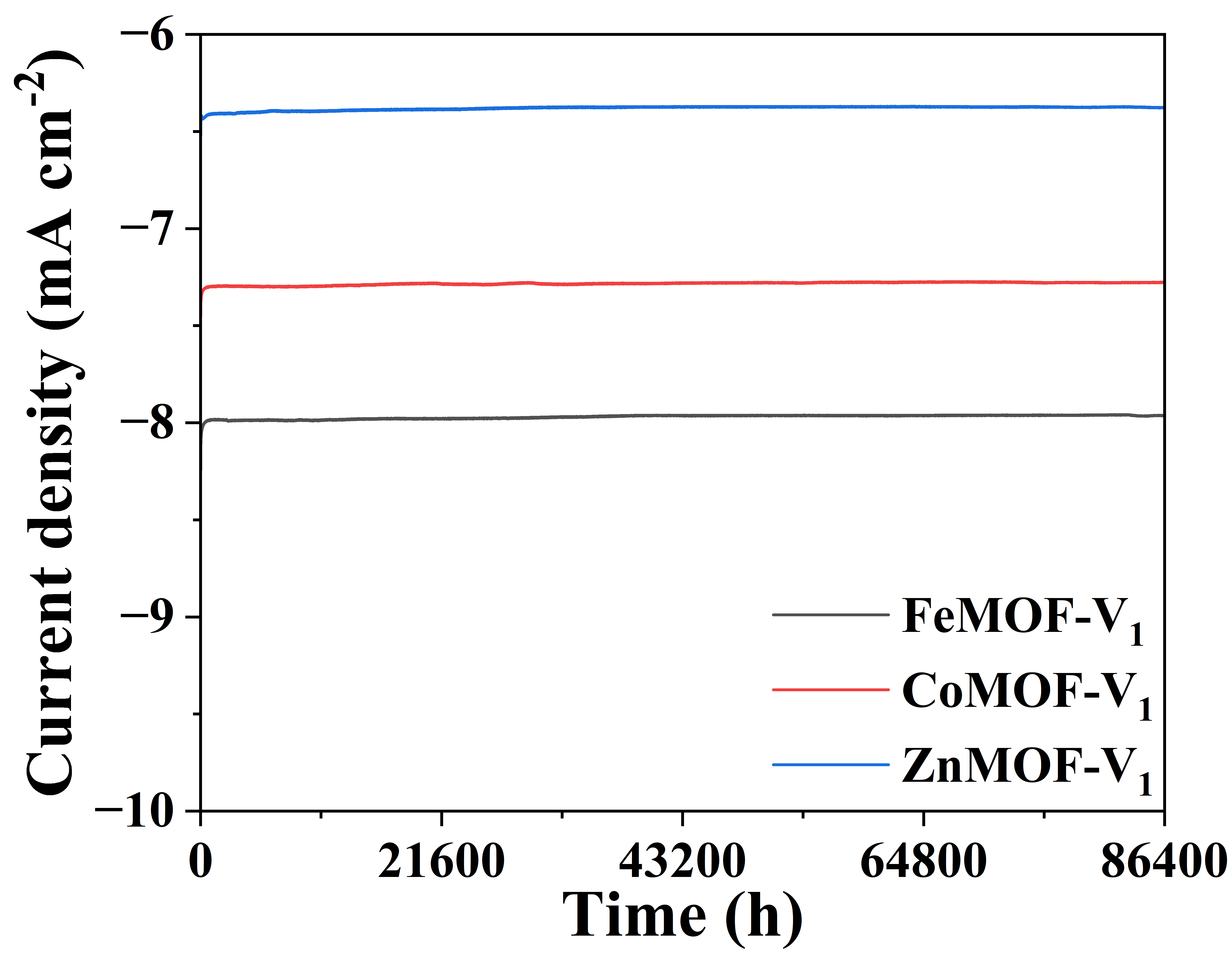


Figure S36. *j*–t curve for 24 h of continuous e-NO_3_RA process


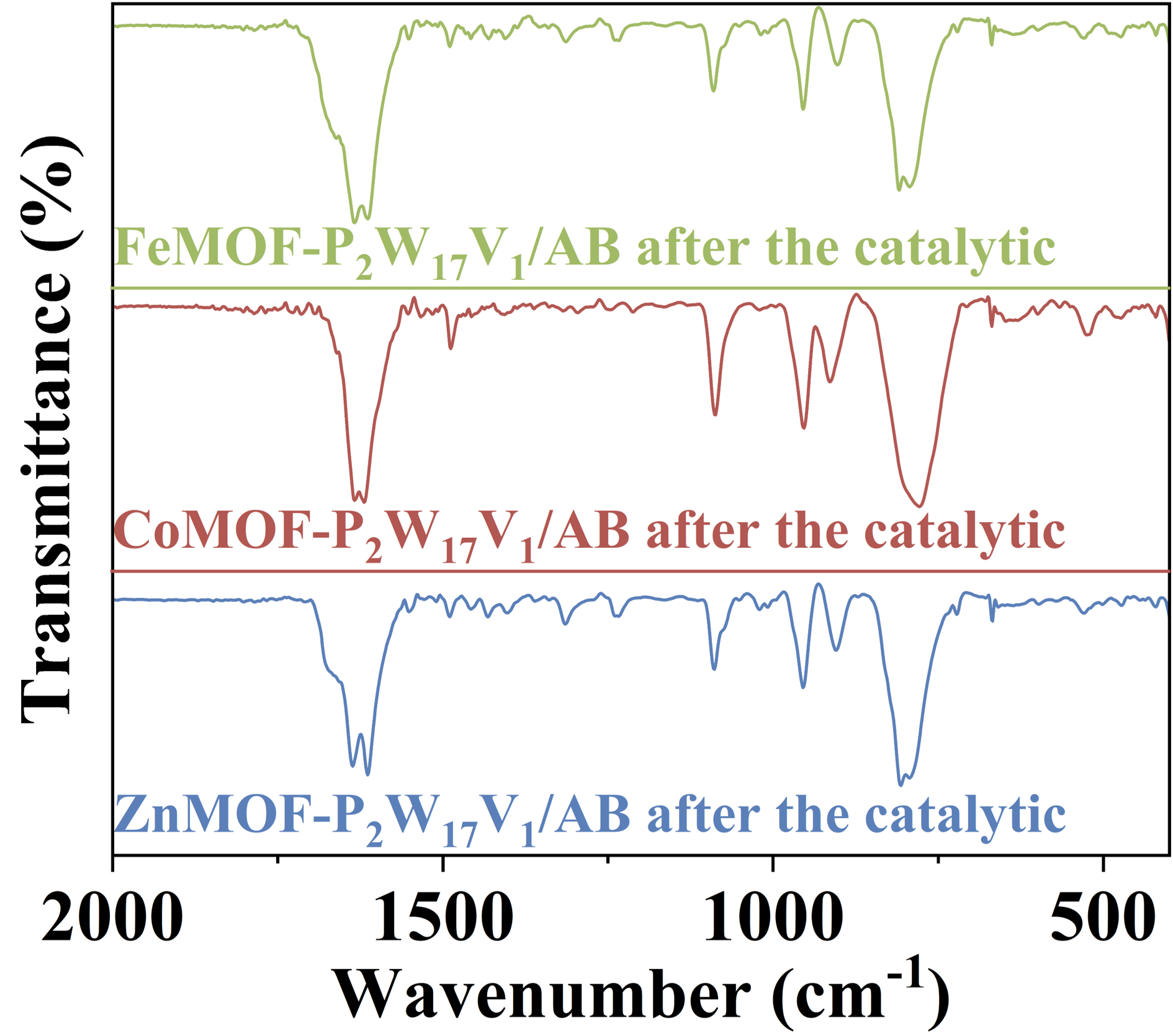


Figure S37. FTIR of XMOF-P_2_W_17_V_1_/AB after catalytic reaction.


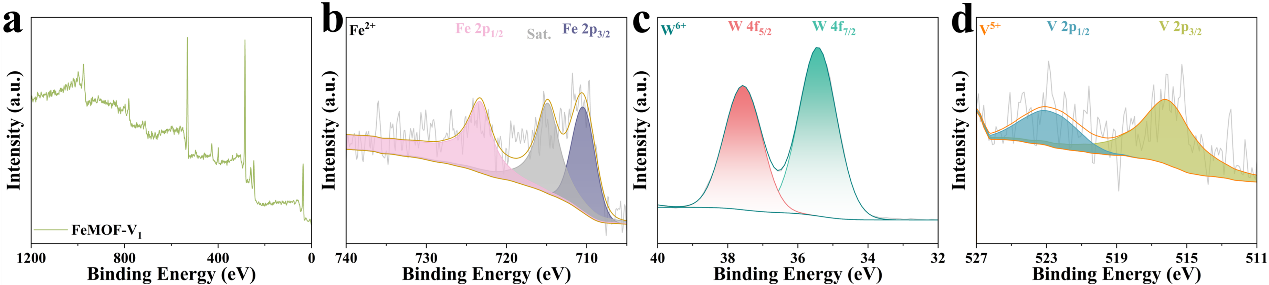


Figure S38. XPS of FeMOF-V_1_ after catalytic reaction, (a) survey, (b) Fe 2p, (c) W 4f, (d) V 2p.


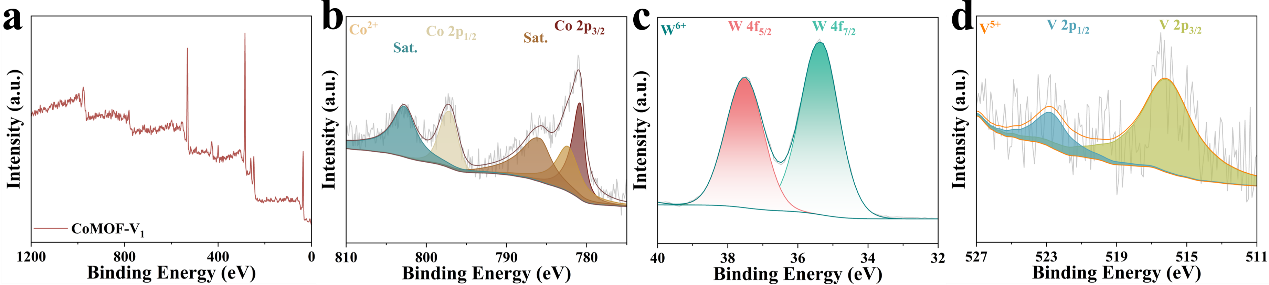


Figure S39. XPS of CoMOF-V_1_ after catalytic reaction. (a) survey, (b) Co 2p, (c) W 4f, (d) V 2p.


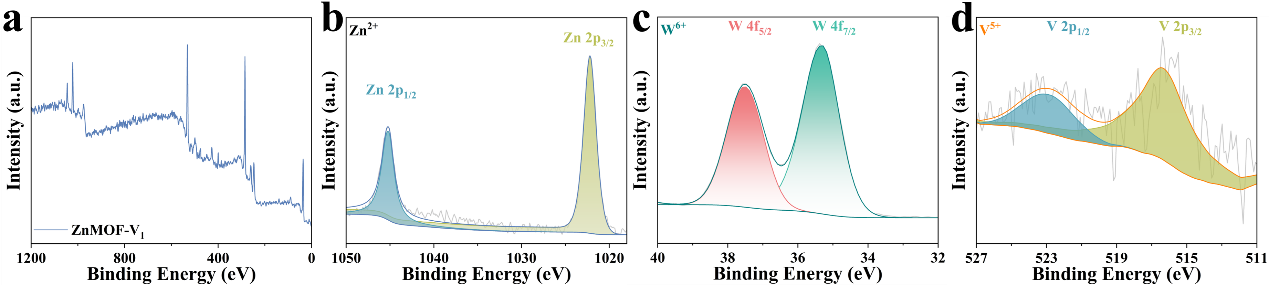


Figure S40. XPS of ZnMOF-V_1_ after catalytic reaction. (a) survey, (b) Zn 2p, (c) W 4f, (d) V 2p.


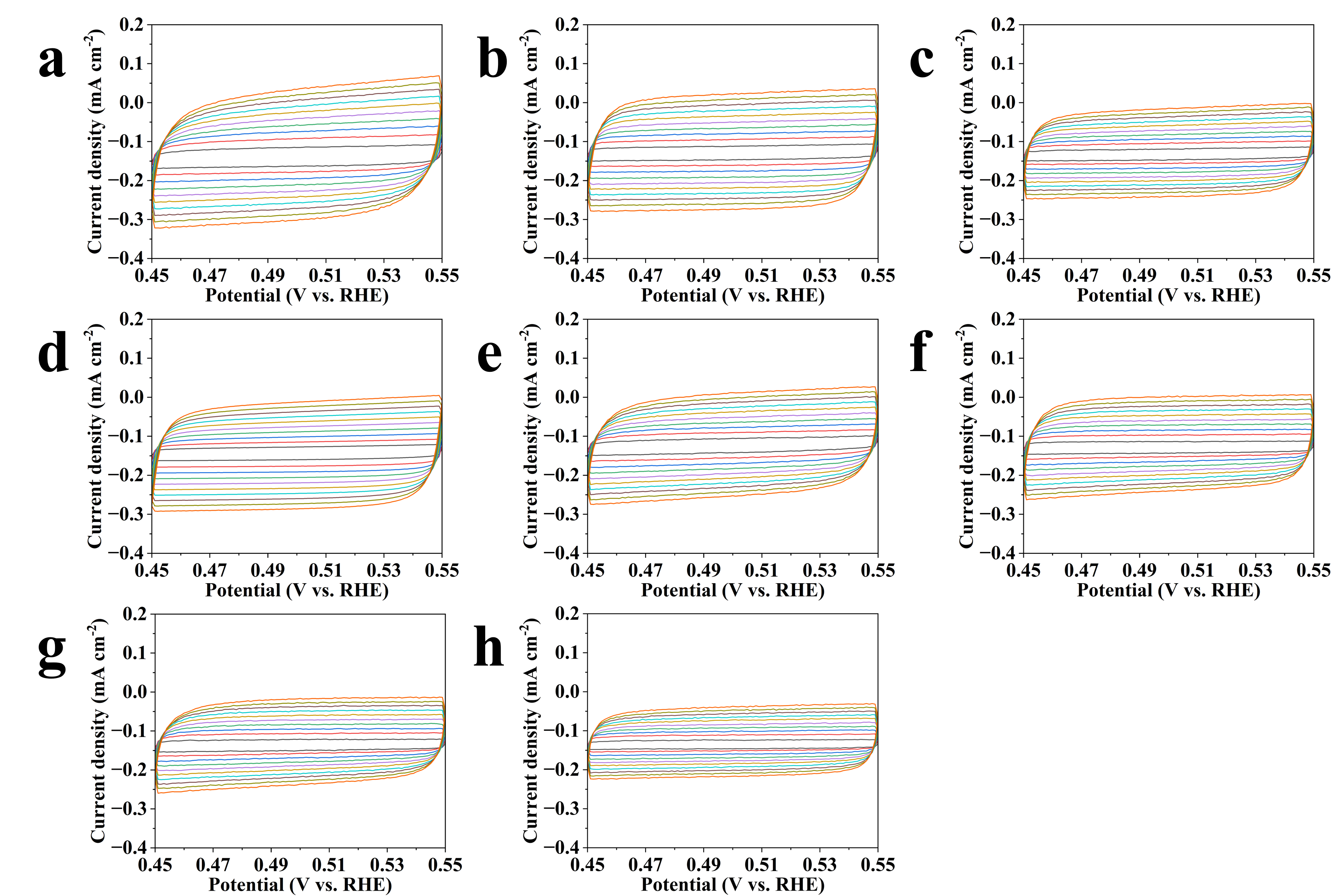


Figure S41. CV curves at non-faradaic potentials of (a) FeMOF-V_1_, (b) CoMOF-V_1_,

(c) ZnMOF-V_1_, (d) FeMOF, (e) CoMOF, (f) ZnMOF, (g) P_2_W_17_V_1_, and (h) FeMOF+P_2_W_17_V_1_.


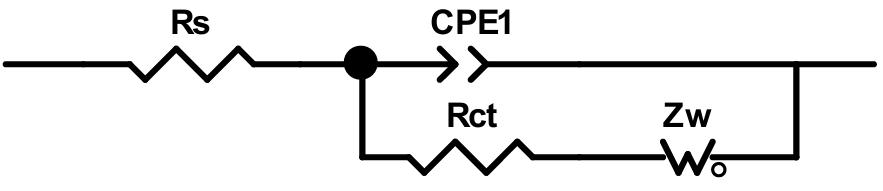


Figure S42. Electrochemical impendence spectra fitted circuit diagram of all the studied samples.


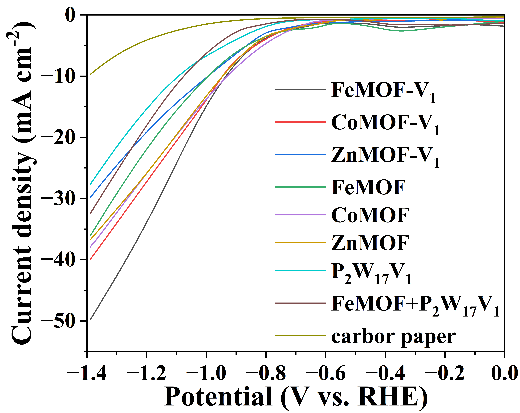


Figure S43. LSV of XMOF-V_1_, XMOF, P_2_W_17_V_1_, FeMOF+P_2_W_17_V_1_, and carbor paper.


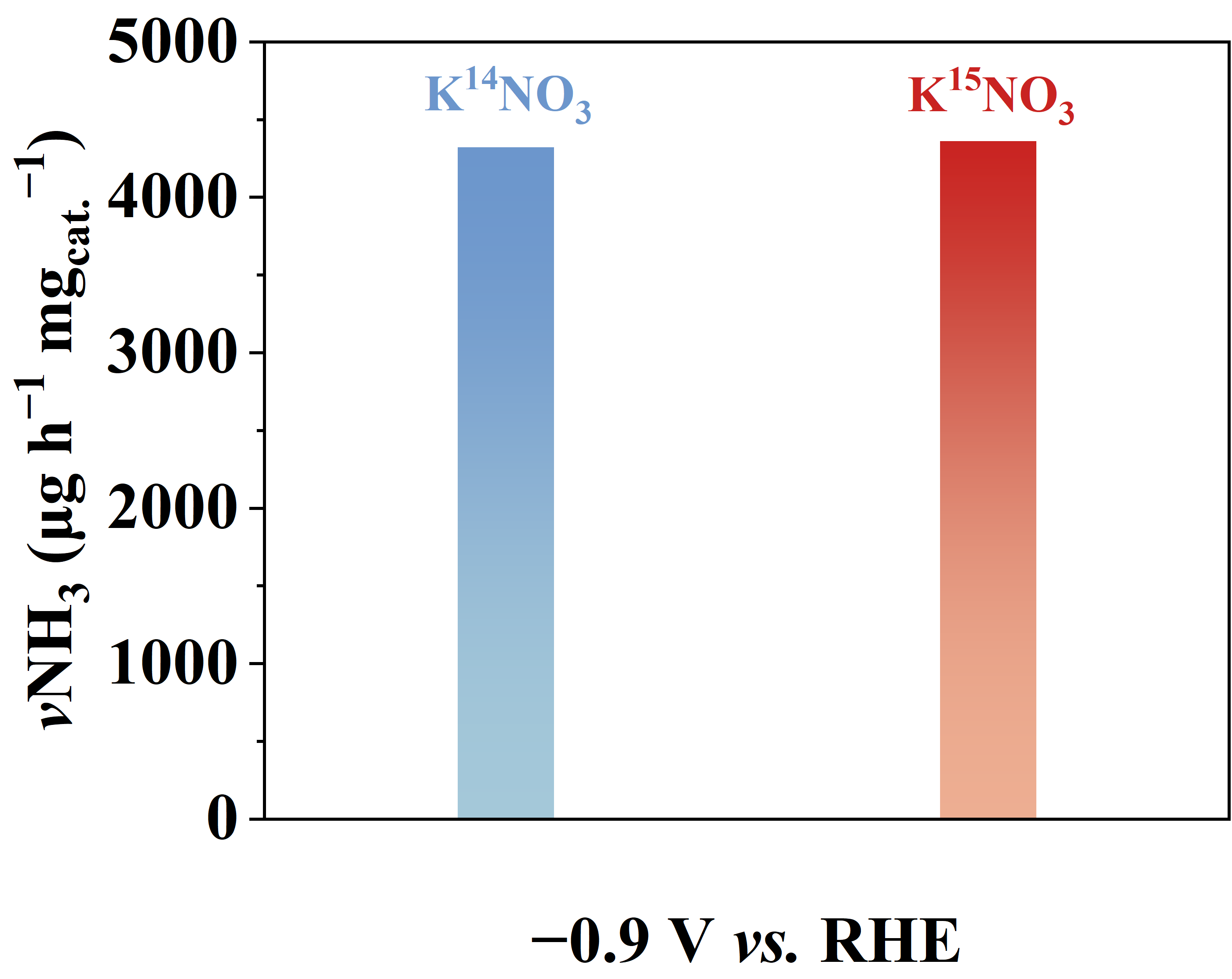


Figure S44. NH_3_ yield rates obtained for FeMOF-V_1_ using K^14^NO_3_ and K^15^NO_3_ as electrolytes.


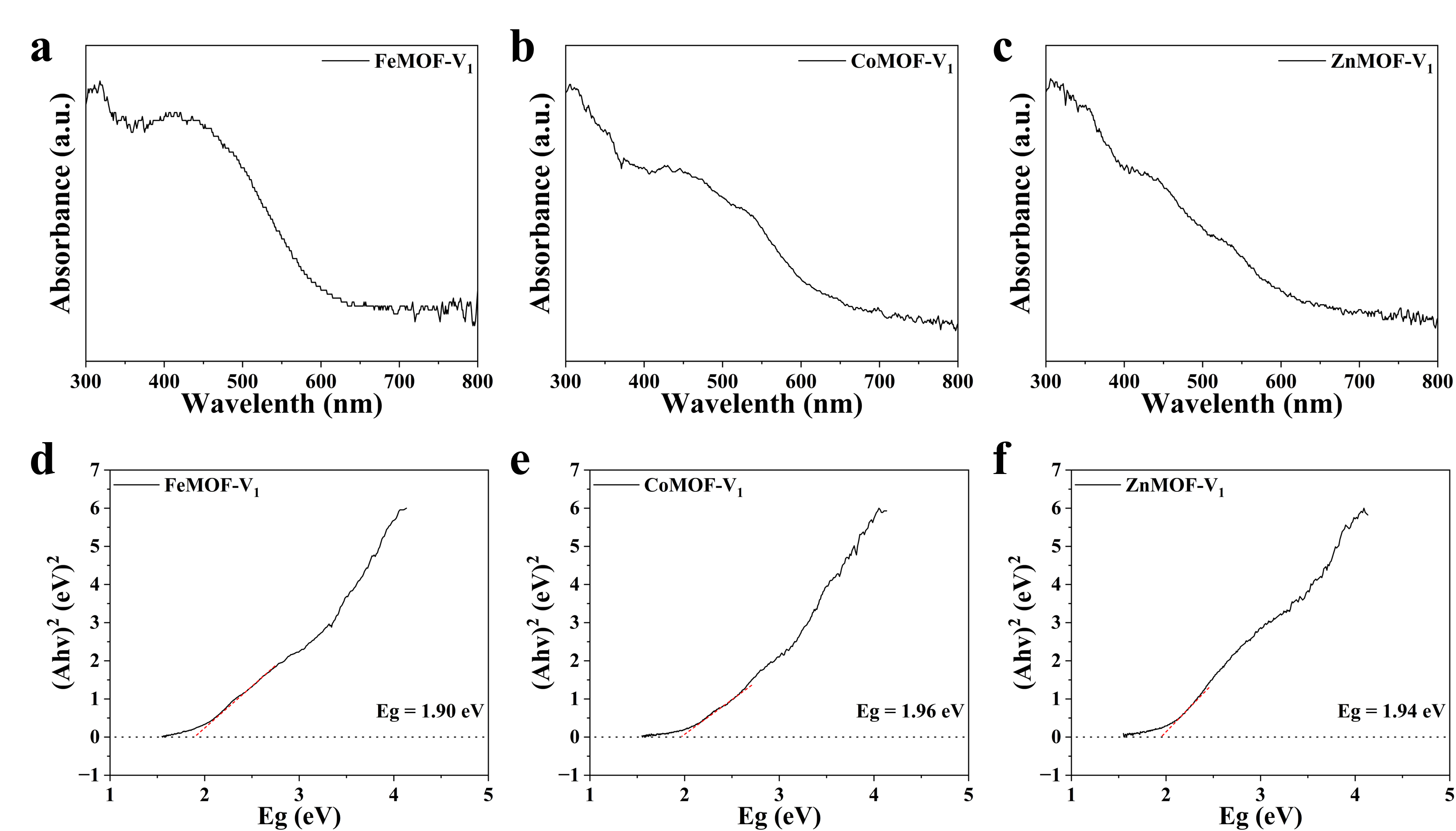


Figure S45. UV-Visible absorption spectra of (a) FeMOF-V_1_, (b) CoMOF-V_1_, and (c) ZnMOF-V_1_. Tauc plot of (d) FeMOF-V_1_, (e) CoMOF-V_1_, and (f) ZnMOF-V_1_.


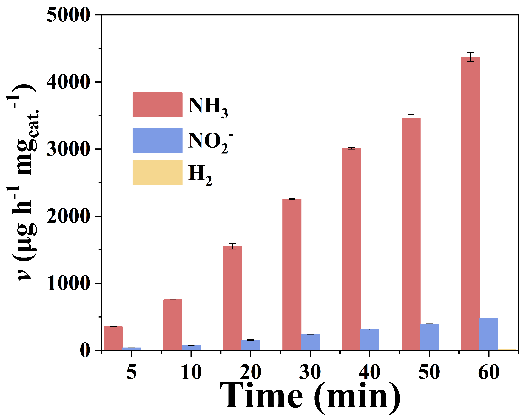


Figure S46. Electrocatalytic performances of FeMOF-V_1_ at −0.9 V vs. RHE with different time. Obtained NH_3_ yield rates by indophenol blue method.


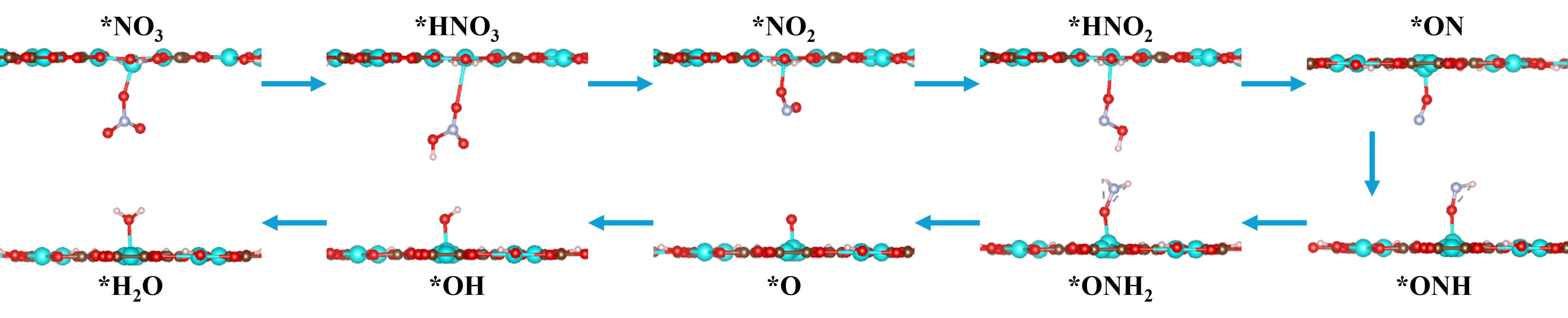


Figure S47. The optimized geometric structures of intermediates corresponding to the O-end pathways followed by the NO_3_^−^ conversion in FeMOF-V_1_. Color scheme for balls: Fe (blue), C (brown), N (wathet), O (red) , H (pink) respectively.


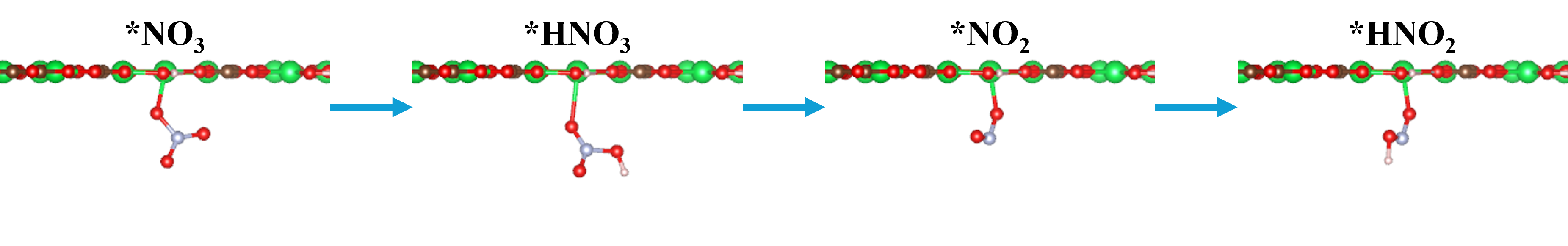


Figure S48. The optimized geometric structures of intermediates corresponding to the pathways followed by the NO_3_^−^ conversion in CoMOF-V_1_. Color scheme for balls: Co (green), C (brown), N (wathet), O (red) , H (pink) respectively.


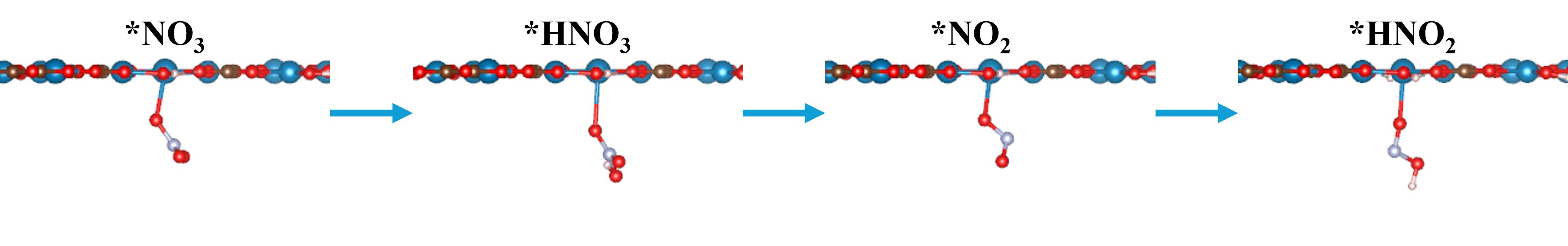


Figure S49. The optimized geometric structures of intermediates corresponding to the pathways followed by the NO_3_^−^ conversion in ZnMOF-V_1_. Color scheme for balls: Zn (deep blue), C (brown), N (wathet), O (red) , H (pink) respectively.


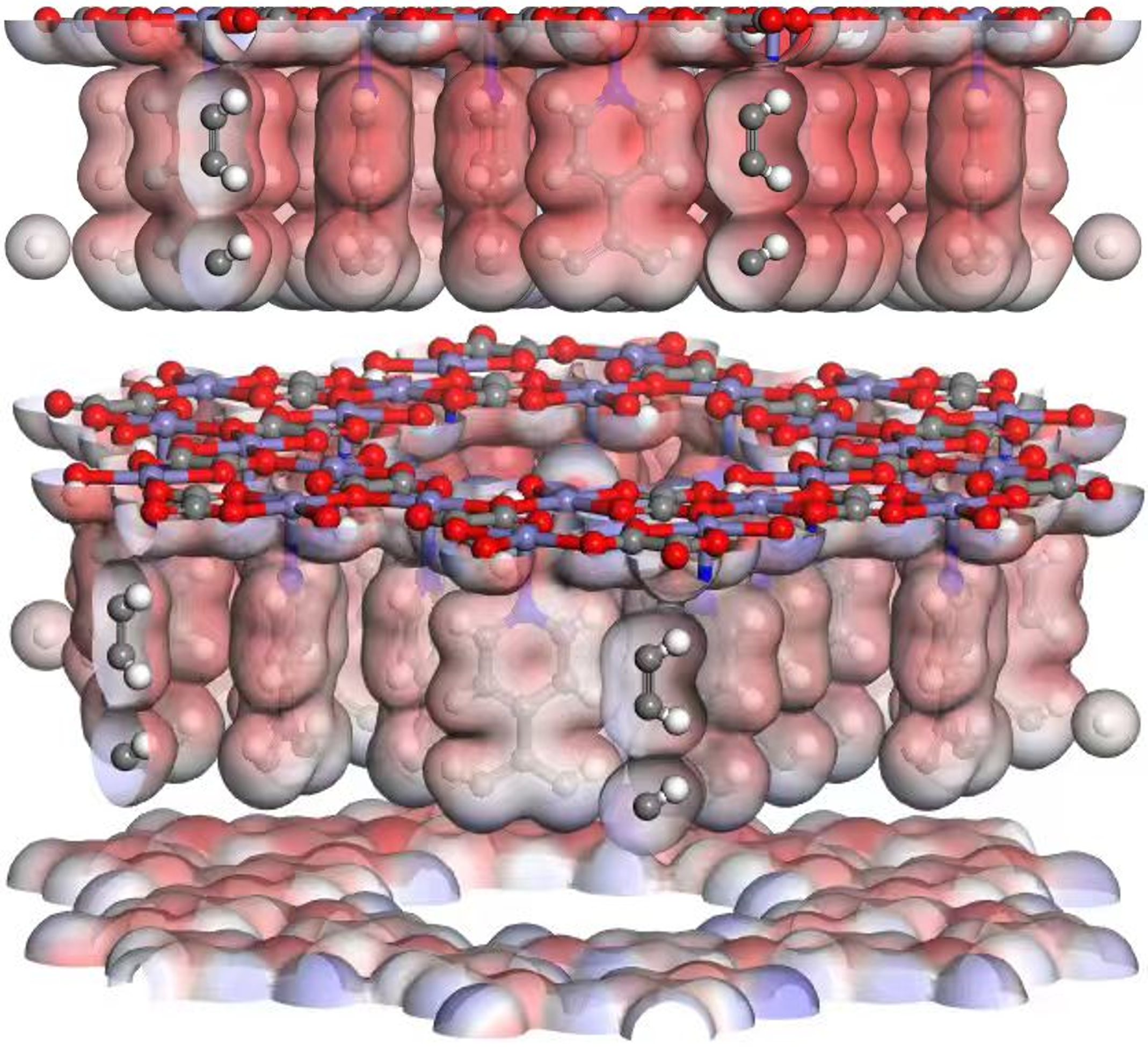


Figure S50. Electrostatic potential (ESP) surface of the FeMOF-V_1_ model.

Table S1. V:W ratios (in atomic %) in the different samples found by EDS and XPS analysis.

| **Sample** | **Synthetic V:W ratio** | **EDS V:W** | **XPS V:W** |
| --- | --- | --- | --- |
| FeMOF-V_1_ | 1 : 17 | 1 : 17.33 | 1 : 16.77 |
| CoMOF-V_1_ | 1 : 17 | 1 : 16.80 | 1 : 17.29 |
| ZnMOF-V_1_ | 1 : 17 | 1 : 17.56 | 1 : 16.84 |

Table S2. Fitted peak area for the V and W XPS spectra in XMOF-V1.

| **Sample** | **V area** | **W area** |
| --- | --- | --- |
| FeMOF-V_1_ | 613.14 | 10548.69 |
| CoMOF-V_1_ | 594.18 | 10349.80 |
| ZnMOF-V_1_ | 807.80 | 13580.85 |

Table S3. Comparison of e-NO_3_RA activity of our catalysts with other electrocatalysts

| **Electrocatalyst** | **Potential**  **V νs. RHE** | **NH_3_ yield rate** | **FE**  **%** | **Reference** |
| --- | --- | --- | --- | --- |
| FeMOF-V_1_ | –1.4 | 13.25 ^a^ | 96.36 | **This**  **work** |
| CoMOF-V_1_ | –1.4 | 5.922 ^a^ | 46.80 |  |
| ZnMOF-V_1_ | –1.4 | 1.959 ^a^ | 30.55 |  |
| FeMOF | –1.4 | 7.622 ^a^ | 77.34 |  |
| CoMOF | –1.4 | 6.520 ^a^ | 46.66 |  |
| ZnMOF | –1.4 | 1.049 ^a^ | 21.87 |  |
| P_2_W_17_V_1_ | –1.4 | 4.240 ^a^ | 46.85 |  |
| FeMOF+P_2_W_17_V_1_ | –1.4 | 9.976 ^a^ | 86.00 |  |
| Fe_2_Fe-MOF | –1.1 | 2.915 ^a^ | 93.16 | [5] |
| Fe_2_Co-MOF | –1.1 | 3.463 ^a^ | 90.55 | [5] |
| Fe_2_Ni-MOF | –1.1 | 2.396 ^a^ | 86.85 | [5] |
| Fe_2_Zn-MOF | –1.1 | 2.478 ^a^ | 83.66 | [5] |
| GeMo-CoNi | –1.3 | 15.69 ^a^ | 87.76 | [6] |
| GeMo-Co | –1.4 | 14.14 ^a^ | 85.15 | [6] |
| GeMo-Ni | –1.4 | 13.67 ^a^ | 86.15 | [6] |
| Co-MOF/FeMo_6_ | –0.8 | 9.23 ^a^ | 86.1 | [7] |
| Co-MOF/CoMo_6_ | –0.8 | 8.64 ^a^ | 96.25 | [7] |
| Co-MOF/NiMo_6_ | –0.8 | 12.34 ^a^ | 98.2 | [7] |
| Co(OH)F/CF | –1.3 | 3.65 ^a^ | 94.17 | [8] |
| CoPB-C8 | –1.54 | 2.74 ^a^ | 93.2 | [9] |
| Co-P/TP | –0.6 | 0.41 ^a^ | 93.6 | [10] |
| Co/Co_3_O_4_ | –1.7 | 8.32 ^a^ | 67 | [11] |
| Fe-Co_3_O_4_ | –0.7 | 0.624 ^a^ | 95.5 | [12] |
| Fe-pyNDI | –1.1 | 14.677 ^a^ | 87 | [13] |
| Fe_2_N@NC | –0.5 | 10.5 ^a^ | 96.11 | [14] |
| MnO_2_-2D Ti_3_C_2_T_x_ | –1.2 | 14.06 ^a^ | 85.23 | [15] |
| Co-bpta-btc | −1.0 | 10.3 ^a^ | 83.3 | [16] |
| FeCu-NC | −1.0 | 6.13 ^a^ | 95 | [17] |
| Fe@Fe_3_O_4_/FF | −1.6 | 4.76 ^a^ | 95.7 | [18] |
| Co75Si15Fe10 | −0.585 | 0.073 ^a^ | 58.7 | [19] |
| Co_7_Cu_3_@PM | −0.7 | 7.43 ^a^ | 95.9 | [20] |
| MnO_2−x_ | −1.0 | 3.34 ^a^ | 92.4 | [21] |
| Co–POM | -0.1 | 7.48 ^b^ | 98 | [22] |
| Mox@SnO_2_ | -0.2 | 2.55 ^b^ | 94 | [23] |
| a: mg h^−1^ mg_cat._^−1^, b : mg h^−1^ cm^−2^. | | | | |

Table S4. Nitrate conversion rate and selectivity of XMOF-V_1_

|  | Conversion rate of NH_3_  % | Selectivity of NH_3_  % |
| --- | --- | --- |
| FeMOF-V_1_ | 15.59 | 98.13 |
| CoMOF-V_1_ | 6.97 | 90.09 |
| ZnMOF-V_1_ | 2.31 | 51.78 |
| FeMOF | 8.97 | 92.44 |
| CoMOF | 7.76 | 96.18 |
| ZnMOF | 1.23 | 40.51 |
| P_2_W_17_V_1_ | 4.99 | 73.03 |
| FeMOF+P_2_W_17_V_1_ | 11.50 | 96.40 |

Table S5. ICP analysis for XMOF-V_1_

| **Compounds** | Element Concentration (μg/L) | |
| --- | --- | --- |
|  | W | V |
| FeMOF-V_1_ | 5.68×10^-5^ | 0.71×10^-5^ |
| CoMOF-V_1_ | 2.18×10^-5^ | 1.16×10^-5^ |
| ZnMOF-V_1_ | 6.82×10^-5^ | 2.44×10^-5^ |

Table S6. Measurements of electrochemical impedance spectroscopy (EIS).

| **Compounds** | **Rs^a^** | **Rct^b^** |
| --- | --- | --- |
| FeMOF-V_1_ | 12.03 | 1.305 |
| CoMOF-V_1_ | 12.05 | 1.732 |
| ZnMOF-V_1_ | 12.07 | 3.529 |
| FeMOF | 12.5 | 6.25 |
| CoMOF | 12.62 | 7.283 |
| ZnMOF | 12.86 | 2.872 |
| P_2_W_17_V_1_ | 11.74 | 7.248 |
| FeMOF+P_2_W_17_V_1_ | 12.75 | 3.244 |
| ^a^Rs reflects the resistance of electrolyte; ^b^Rct reflects the charge-transfer process | | |

**Supplementary References**

[1] M. Abbessi, R. Contant, R. Thouvenot, G. Hervé, *Inorg. Chem.* (United States) **1991**, *30*.

[2] P. E. Blöchl, *Phys. Rev. B* **1994**, *50*, 17953–17979.

[3] J. P. Perdew, K. Burke, M. Ernzerhof, *Phys. Rev. Lett.* **1996**, *77*, 3865–3868.

[4] G. Kresse a, J. Furthmüller, *Set. Comput. Mater. Sci.* **1996**, *6*, 15−50.

[5] Y. Lv, S. Ke, Y. Gu, B. Tian, L. Tang, P. Ran, Y. Zhao, J. Ma, J. Zuo, M. Ding, *Angew. Chem. Int. Ed.* **2023**, *62*, e202305246.

[6] J. Li, M.Yang, A. Tian, X. Cao, J. Ying, X. Wang, *Chem. Eng. J.* **2025**, *521*, 166862.

[7] Q. Zhou, X. Wang, S. Rong, G. Li, Q. Jiang, H. Pang, H. Ma, *Inorg. Chem.* **2025**, *64*, 5291–5301.

[8] Y. Ye, O. Guo, L. Wang, Z. Li, Z. Song, J. Chen, Z. Zhang, S. Xiang, O. Chen, *J. Am. Chem. Soc.* **2017**, *139*, 15604−15607.

[9] F. Zhang, L. Dong, O. Qin, W. Guan, J. Liu, S. Li, M. Lu, Y. Lan, Z. Su, H. Zhou, *J. Am. Chem. Soc.* **2017**, *139*, 6183−6189.

[10] X. Wang, Q. Hong, L. Shao, Q. Zhai, Y. Jiang, X. Ai, Y. Chen, S. Li, *Adv. Funct. Mater.* **2024**, *34*, 2408834.

[11] L. An, M. Narouz, P. Smith, P. Torre, C. Chang, *Angew. Chem. Int. Ed.* **2023**, *62*, e202305719.

[12] P. Wei, J. Liang, Q. Liu, L. Xie, Xin Tong, Yuchun Ren, T. Li, Y. Luo, N. Li, B. Tang, A. Asiri, M. Hamdy, Q. Kong, Z. Wang, X. Sun, *Journal of Colloid Science* **2022**, *615*, 636−642.

[13] Z. Xue, M. Yao, K. Otake, Y. Nishiyama, Y. Aoyama, J. Zheng, S. Zhang, T. Kajiwara, S. Horike, S. Kitagawa, *Angew. Chem. Int. Ed.* **2024**, *63*, e202401005.

[14] Y. Chen, T. Rao, J. Zhan, L. Zhang, F. Yu, *Chem. Commun.* **2025**, *61*, 7684−7687.

[15] T. Phu, T. Pham, A. Nguyen, T. Tran, T. Tran, Ng. Le, P. Nguyen, T. Phung, *iScience* **2025**, *28*, 112729.

[16] L. Qin, Q. Liu, Y. Wang, J. An, M. Zhang, C. Shi, *Inorg. Chem.* **2025**, *64*, 27, 14012−14019.

[17] X. Meng, K. Wang, Z. Zhao, K. Li, W. Sun, Y. Lin, *Small* **2025**, *21*, 2407216.

[18] Y. Liu, Y. Zhu, W. Duan, Y. Yang, H. Tuo, C. Feng, *Chin. Chem. Lett.* **2025**, *36*, 110347.

[19] I. Kuznetsova, D. Kultin, O. Lebedeva, S. Nesterenko, L.Fishgoit, A. Leonov, L. Kustov, *Electrocatalysis* **2025**, *16*, 1011−1019.

[20] R. Guo, Z. Cui, T. Yu, J. Li, W. Peng, J. Liu, *AIChE J.* **2025**, *71*, e18628.

[21] G. Wang, P. Shen, Y. Luo, X. Li, X. Li, K. Chu, *Dalton Trans.* **2022**, *51*, 9206−9212.

[22] D Garg, P Kumar, L Mallick, K Samanta, B Chakraborty. ChemSusChem 2025, 18, e202501665.

[23] L Mallick, H V.Annadata, B Chakraborty. ACS Appl. Mater. Interfaces 2024, 16, 32385−32393.
